# Platelet Count and Survival after Cancer

**DOI:** 10.3390/cancers14030549

**Published:** 2022-01-21

**Authors:** Vasily Giannakeas, Joanne Kotsopoulos, Jennifer D. Brooks, Matthew C. Cheung, Laura Rosella, Lorraine Lipscombe, Mohammad R. Akbari, Peter C. Austin, Steven A. Narod

**Affiliations:** 1 Women’s College Research Institute, Women’s College Hospital, Toronto, ON M5S 1B2, Canada; vasily.giannakeas@wchospital.ca (V.G.); joanne.kotsopoulos@wchospital.ca (J.K.); lorraine.lipscombe@wchospital.ca (L.L.); mohammad.akbari@utoronto.ca (M.R.A.); 2 Dalla Lana School of Public Health, University of Toronto, Toronto, ON M5T 3M7, Canada; jennifer.brooks@utoronto.ca (J.D.B.); laura.rosella@utoronto.ca (L.R.); 3 ICES, Toronto, ON M4N 3M5, Canada; matthew.cheung@sunnybrook.ca (M.C.C.); peter.austin@ices.on.ca (P.C.A.); 4 Division of Medical Oncology and Hematology, Odette Cancer Centre, Sunnybrook Health Sciences Centre, Toronto, ON M4N 3M5, Canada; 5 Department of Medicine, University of Toronto, Toronto, ON M5S 1A8, Canada; 6 Division of Endocrinology, Women’s College Hospital, Toronto, ON M5S 1B2, Canada; 7 Institute of Medical Science, University of Toronto, Toronto, ON M5S 1A8, Canada; 8 Institute of Health Policy Management and Evaluation, University of Toronto Ontario Canada, Toronto, ON M5T 3M6, Canada

**Keywords:** platelet count, platelets, thrombocytosis, cancer survival, marginal structural model

## Abstract

**Simple Summary:**

Platelets are cellular fragments circulating in the blood that are responsible for clotting. Previous research has shown that cancer patients with an abnormally high platelet count (thrombocytosis) have elevated rates of death from cancer. We aimed to investigate to what extent platelet counts are associated with survival after cancer. We followed a large provincial cohort of cancer patients with a platelet count recorded at the time of their diagnosis. We categorized patients according to platelet count (low, medium, high). Cancer patients in the ‘high’ platelet count category had the highest rate of cancer death, and cancer patients in the ‘low’ platelet count category had the lowest rate of cancer death. Platelet count may be used to predict survival in cancer patients.

**Abstract:**

Thrombocytosis is associated with cancer progression and death for many cancer types. It is unclear if platelet count is also associated with cancer survival. We conducted a cohort study of 112,231 adults in Ontario with a diagnosis of cancer between January 2007 and December 2016. We included patients who had a complete blood count (CBC) completed in the 30 days prior to their cancer diagnosis. Subjects were assigned to one of three categories according to platelet count: low (≤25th percentile), medium (>25 to <75th percentile), and high (≥75th percentile). Study subjects were followed from the date of their cancer diagnosis for cancer-specific death. Of the 112,231 eligible cancer patients in the cohort study, 40,329 (35.9%) died from their cancer in the follow-up period. Relative to those with a medium platelet count, the rate of cancer-specific death was higher among individuals with a high platelet count (HR 1.52; 95%CI 1.48–1.55) and was lower among individuals with a low platelet count (HR 0.91; 95%CI 0.88–0.93). A high platelet count was associated with poor survival for many cancer types. Platelet count could potentially be used as a risk stratification measure for cancer patients.

## 1. Introduction

Platelets play a key role over the natural history of many malignant neoplasms and contribute to local tumor growth, dissemination, and metastasis [1,2,3]. At the site of a tumor, activated platelets release cytokines that facilitate tumor growth and angiogenesis [4,5,6]. Platelets may also bind to circulating tumor cells in the bloodstream, thereby shielding tumor cells from a potential attack from immune cells [1,3,7]. Platelets adhere to the endothelial cells in the blood vessels and mediate the extravasation of tumor cells from the bloodstream to metastatic sites [3]. Platelet count may also be a marker of disease progression among cancer patients. Platelet production homeostasis can be disrupted by the inflammatory cytokine milieu (e.g., disseminated intravascular coagulation) [8] and/or metastasis within bone marrow [9] which are associated with several malignancies.

For many cancer types, thrombocytosis (an abnormally high platelet count) has been shown to be associated with poor prognosis. Among colon cancer patients, pre-operative thrombocytosis is correlated with both cancer stage and survival [10,11,12]. Similar findings have been reported for lung cancer [13,14,15], gastric cancer [16,17], renal cancer [18,19], breast cancer [20], melanoma [21], and ovarian cancer [22,23]. It is unclear to what extent platelet counts across a wide range (including both high and low counts) are associated with cancer survival. It is not known whether platelet counts are a marker of advanced stage disease or if the association of platelet count and survival is independent of stage. 

We identified a cohort of adults in Ontario, newly diagnosed with cancer, that had a complete blood count (CBC) assayed in the 30-day period prior to their diagnosis. We followed the patients for up to 10 years to determine whether platelet count was associated with survival.

## 2. Materials and Methods

### 2.1. Study Design, Population, and Data

Our patient population consisted of Ontario residents diagnosed with cancer between 1 January 2007 and 31 December 2016. For each subject, we retrieved all platelet counts from the CBC test records that were made available to us through the provincial laboratory database. The dates of death and causes of death were captured up to 31 December 2017. Data analysis was performed in February 2021.

The province of Ontario, Canada provides universal healthcare coverage for all 14.5 million residents through the Ministry of Health. Provincial health coverage includes access to primary care services, emergency department visits, inpatient hospitalizations, and medication use (among adults aged 65 and older). Administrative health data dating back to 1988 has been centralized and linked for research purposes. ICES (formerly known as the Institute for Clinical and Evaluative Sciences) is a non-profit organization that provides researchers with analytic services to conduct large population-level studies using administrative health data. ICES is a prescribed entity under section 45 of Ontario’s Personal Health Information Protection Act.

The ICES data repository includes over 100 datasets that are linked using a unique encoded personal identifier for each Ontario resident. Recently, ICES expanded their repository to include laboratory test data dating back to 2007. The Ontario Laboratory Information System (OLIS) dataset includes over 85 million CBC tests among 9.5 million Ontario residents reported between 2007 and 2017. Incident cancer and treatment data are available through multiple sources. The Ontario Cancer Registry (OCR) includes cancer information for incident cancers diagnosed in Ontario from January 1964 [24]. Cancer treatment, including chemotherapy and radiotherapy, was obtained from the Discharge Abstract Database (DAD), the Same Day Surgery (SDS) database, the National Ambulatory Care and Reporting System (NACRS), the Ontario Health Insurance Plan (OHIP) claims database, the Cancer Activity Level Reporting (ALR) database, and the New Drug Funding Program (NDFP) database. Descriptions of each database and their application in this research is available in Table S1.

### 2.2. Construction of the Cohort

We identified a cohort of Ontario residents that had a first primary cancer diagnosis reported in the OCR database between 1 January 2007 and 31 December 2016. Subjects were assigned to 1 of 19 cancer sites: colon, lung, breast (female only), ovary, cervix, endometrium, prostate, testicle, thyroid, pancreas, stomach, kidney, bladder, esophagus, other gastrointestinal, brain, melanoma, head and neck, and other solid tumor (definitions provided in Table S2). Liver and hematological cancers were not included in this cohort because these tumor types are biologically associated with platelet production.

We excluded individuals with an unknown date of birth or sex, or if they were under age 18 or over age 100 on the date of their cancer diagnosis. We excluded individuals that were ineligible for provincial health coverage. We excluded individuals with evidence of a prior cancer. Frequencies for inclusion and exclusion criteria are available in Tables S5 and S6. Our final cohort consisted of cancer patients with a platelet count from a CBC record in the 30 days prior to the date of a cancer diagnosis (approximately 25% of all patients diagnosed with cancer in the accrual period) (see Figures S1 and S2).

### 2.3. Exposure Definition

Platelet counts were ascertained for the 30 days prior to the cancer diagnosis up to (and including) the date of the cancer diagnosis. If there were multiple platelet counts recorded in this time period, the earliest record was selected. The distribution of platelet count was determined by sex for each cancer type. Descriptive statistics including the mean, standard deviation, 10th, 25th, 50th, 75th, and 90th percentiles were computed for each platelet count distribution (Table S9). Platelet count was assigned to one of three categories based on the sex- and cancer-specific distribution values: low (≤25th percentile), medium (>25 to <75th percentile), and high (≥75th percentile).

### 2.4. Outcome Definition

The index date was defined as the date of diagnosis of the index cancer. Cancer patients were followed from their index date to the first of either cancer-specific death, the end of provincial health coverage, other cause-of-death, or the end of the observation period (31 December 2017). Cancer-specific death was defined using the attributable cause-of-death from the Office of the Registrar General Deaths (ORGD) dataset (see Table S4). The underlying cause-of-death ICD-9 code was used to assign a death attributable to the index cancer (cancer-specific death) or a non-cancer-specific death (i.e., a competing risk) based on the site of the index cancer.

### 2.5. Baseline Variables

Baseline information was acquired for each patient. Demographic information included age, sex, neighborhood income quintile, and residence location from the Registered Persons Database (RBDB). We obtained medical information prior to the cancer diagnosis, including health services utilization, comorbidities, chronic conditions, and medication use (see Table S3 for definitions). Stage of cancer was available for approximately 66% of all diagnosed cancers but varied significantly by cancer site. The distribution of cancer stage for each site, along with degree of missingness, is available in Table S10.

### 2.6. Baseline Platelet Analysis

We performed a time-to-event analysis to study the association of platelet count and the hazard of cancer-specific death. Analysis was undertaken for the overall cohort and for each cancer site separately. We computed non-parametric cancer-specific death rates for each subgroup by dividing the total number of cancer-specific deaths by the total number of person-years of follow-up. We generated 10-year cancer-specific survival curves for each of the three platelet level categories (low, medium, high). We obtained these survival estimates by computing one minus the cumulative incidence function of cancer-specific death with non-cancer-specific death as a competing risk. We used cause-specific hazard models to estimate the hazard ratio of cancer-specific death for being in the low or a high platelet count category relative to the medium platelet count category (reference) accounting for the competing risk of non-cancer-specific death.

Three models were fitted to assess for potential confounding: model 1 was unadjusted; model 2 adjusted for patient variables which included age at diagnosis, year of diagnosis, and common chronic conditions (asthma, congestive heart failure, chronic obstructive pulmonary disease, hypertension, and diabetes); and model 3 included all patient variables from model 2 as well as cancer stage (subjects with unknown stage were excluded). All statistical analyses were performed using SAS version 9.4 (SAS Institute, Cary, NC, USA).

### 2.7. Time-Dependent Platelet Analysis

As a secondary objective, we were interested in determining whether changes in the platelet count in the follow-up period is associated with cancer survival. We partitioned the observation time into week of follow-up from the index date. CBC records in the follow-up period with a valid platelet count were assigned to a week of follow-up. If multiple platelet count observations occurred in the same week, the observation with the earliest date was selected. If no CBC test occurred within a given follow-up week, we retained the platelet count value from the week prior.

Platelet count as a time-dependent exposure is prone to time-dependent confounding because of the association of platelet count with cancer treatment (i.e., chemotherapy and radiotherapy) (Figure S3). Cancer therapy can temporarily decrease platelet count because of bone marrow suppression by cytotoxic chemotherapy or radiotherapy [25]. Furthermore, cancer therapy is associated with cancer-specific survival, and because a clinically acceptable platelet count is required for future therapy, there is the potential for time-dependent confounding. To address this, we performed additional modeling in our time-dependent analysis, which incorporates Marginal Structural Models (MSM) [26]. First, the data were structured using the weekly exposure definition described above. We then created two binary variables to define an individual as receiving chemotherapy and/or radiotherapy for each week of follow-up. For exposure weights, we used multinomial logistic regression to estimate the probability of being in a platelet count category (low, medium, or high) conditional on an individual’s platelet count status the week prior, age category, index year, common chronic conditions, cancer stage, and chemotherapy treatment and radiotherapy treatment (treatment variables for numerator model only). For censor weights, we conducted a similar approach using binomial logistic regression. Stabilized weights were generated using the probability of treatment and censor estimates from the four model outputs (Figure S4). Stabilized weights were truncated at the 0.5th and 99.5th percentiles based on the weight distribution for each cancer type [27]. Lastly, we applied stabilized weights to a fully adjusted Cox proportional hazards model using robust sandwich variance estimation among within-subject clusters.

In the months preceding a cancer death, patients may experience a rapid drop in platelet count because of cancer progression or metastases within the bone marrow, consumption from disseminated intravascular coagulation, or cachexia. To account for this effect in our time-dependent analysis, we applied a 60-day lag to platelet count observations (along with cancer treatment observations in the MSM analysis) in the follow-up period. Thus, any time-dependent observations that occur within 2 months of a cancer-specific death or censoring event were not included.

We were also interested in transient effects of platelet count in the follow-up period among patients that died from a cancer-specific death. For this analysis, we retained patients that had a cancer-specific death in the follow-up period. For each patient, we captured platelet count in the 2 years preceding their cancer-specific death. We selected a maximum of one platelet count value per patient per week of lookbacks. If multiple platelet count values were available for a given week, the record with the earliest calendar date was selected. For each cancer site, we aggregated the platelet values to report the median platelet count for each week in the 2-year period preceding cancer-specific death. We repeated this procedure on patients that did not die from their index cancer, and on patients that did not die from their index cancer who had a minimum of 5 years of follow-up (measuring platelet count in the first 5 years of follow-up).

### 2.8. Sensitivity Analysis

To assess the robustness of our effect estimates using a more granular exposure definition, we reanalyzed the data based on the following 5-level platelet count definition: very low (≤10th percentile), low (>10 to ≤25th percentile), medium (>25 to <75th percentile), high (≥75 to <90th percentile), and very high (≥90th percentile).

## 3. Results

There were 582,692 Ontario residents diagnosed with a first primary cancer during the accrual period (2007–2016) of whom 112,231 were eligible with a baseline platelet count recorded in the 30-day period prior to their cancer diagnosis (differences between the patients who did and who did not have a platelet count reported are available in Table S7). The median age at diagnosis was 68.0 years (interquartile range 57.8–77.5) and 56,524 (50.4%) were women (Table 1). The most commonly diagnosed cancer sites were lung (18.3%), colon (15.4%), breast (8.8%), and prostate (7.7%). Patients were followed for an average of 2.6 years (range 0–10 years) and 40,329 (35.9%) patients died from their index cancer in the follow-up period.

### 3.1. Baseline Platelet Count Analysis

The median time between the CBC test result and cancer diagnosis was 12 days (interquartile range 4–21). The median baseline platelet count was 256 × 10^9^ platelets/L (interquartile range 208–320). Of the 112,231 patients in the cohort, 6730 (6%) had a platelet count above 450 × 10^9^ platelets/L (the conventional cutoff for thrombocytosis). The median age at death among patients who died from their index cancer was 73.0 years. A detailed descriptive table by platelet count category is presented in Table S8.

**Figure 1:**
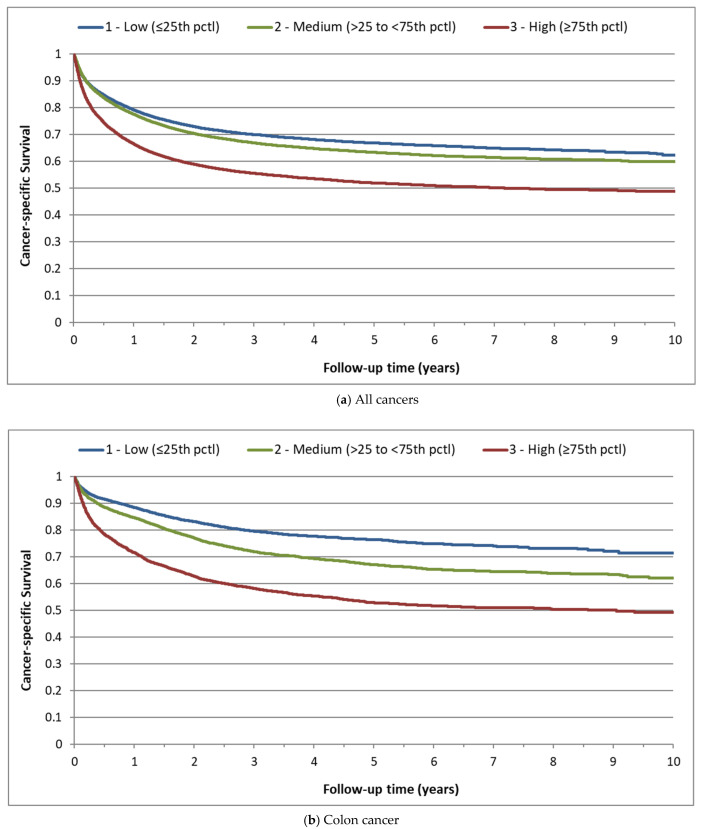

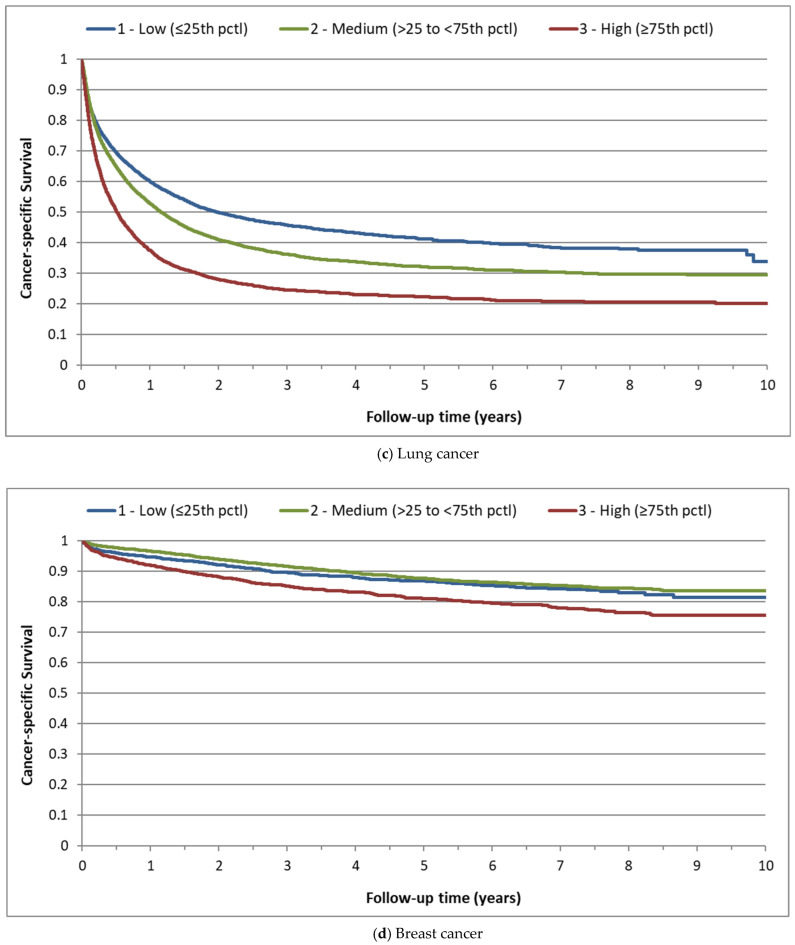

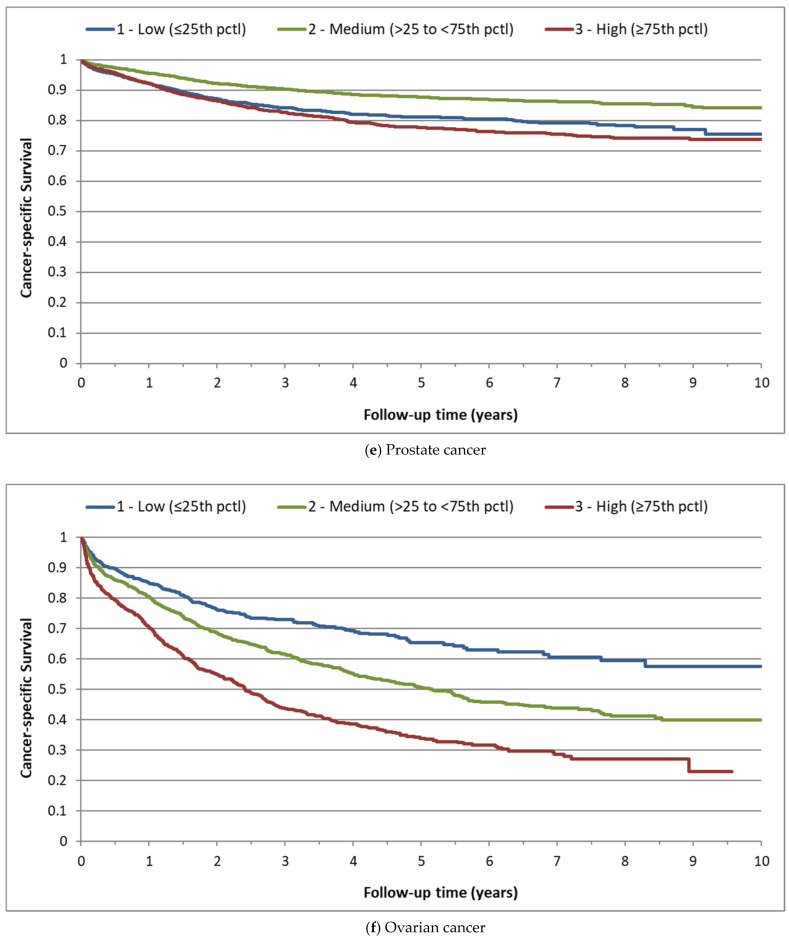

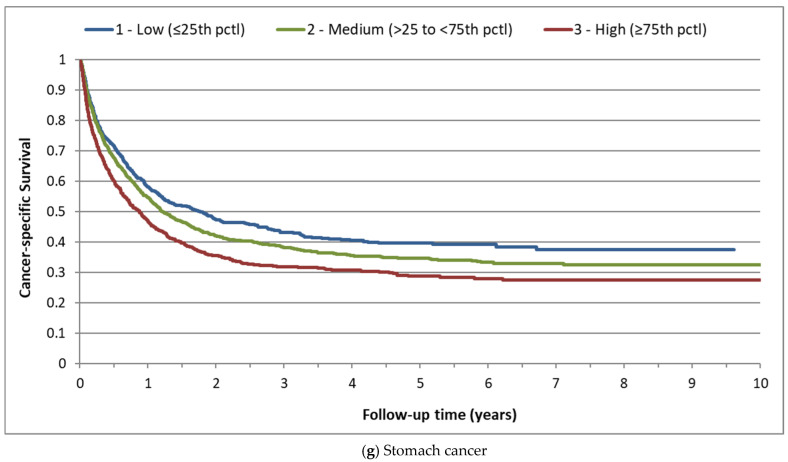
Cancer-specific survival by baseline platelet count category, for select cancers. (**a**) All cancers (*n* = 112,231); (**b**) colon (*n* = 17,259); (**c**) lung (*n* = 20,583); (**d**) breast (*n* = 9857); (**e**) prostate (*n* = 8587); (**f**) ovary (*n* = 3085); (**g**) stomach (*n* = 3365).

The association between baseline platelet count and the hazard of cancer-specific death was significant for all cancers combined and for several individual cancer sites. Cancer-specific survival curves by platelet category are presented in Figure 1a–g and the corresponding hazard ratios are presented in Table 2. Compared to patients with a medium platelet count, colon cancer patients with a high platelet count (≥75th percentile) had a high rate of cancer-specific mortality (HR 1.70; 95%CI 1.60–1.80) whereas those with a low platelet count (≤25th percentile) had a low rate of cancer-specific mortality (HR 0.70; 95%CI 0.65–0.76) (Figure 1b). Similar trends were observed for patients with lung (Figure 1c), ovarian (Figure 1f), and stomach (Figure 1g) cancers.

Among breast and prostate cancer patients, both low and high platelet counts were associated with a relatively high rate of cancer-specific mortality (Figure 1d,e). Among prostate cancer patients, the rate of cancer-specific death was higher both with a low platelet count (HR 1.66; 95%CI 1.45–1.90) and with a high platelet count (HR 1.91; 95%CI 1.68–2.17). Similarly, death rates were high among breast cancer patients with a low platelet count (HR 1.19; 95%CI 1.03–1.37) and with a high platelet count (HR 1.73; 95%CI 1.52–1.97). The results for the other cancer sites are provided in Table S11. For most cancers, findings were similar when platelet count was treated as a 5-level categorical exposure (Table S12, Figure S5).

**Figure 2:**
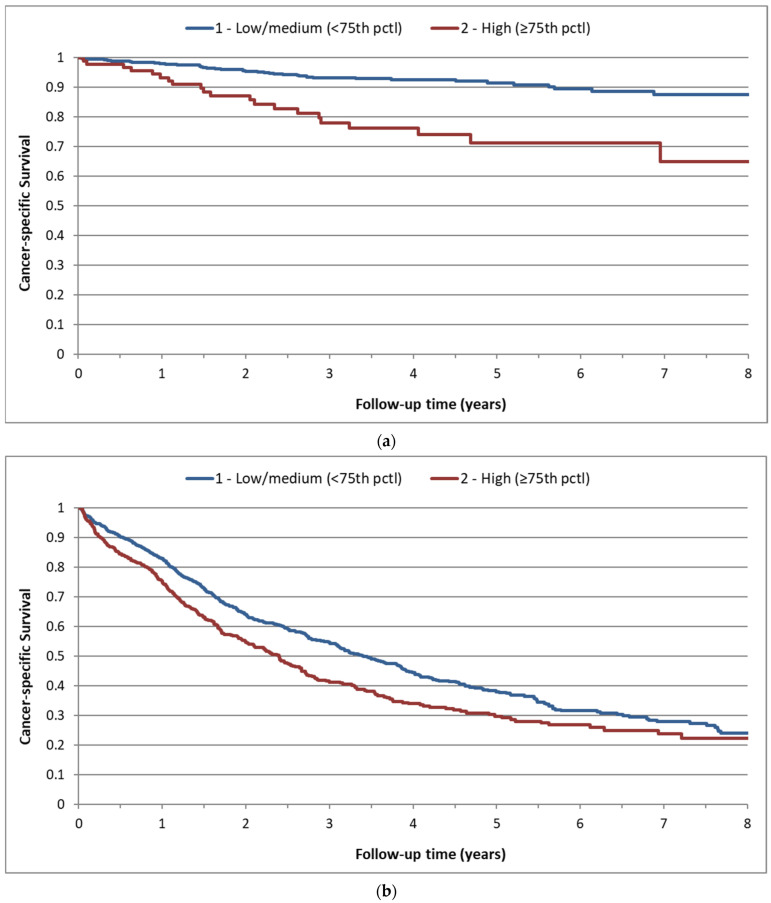
Cancer-specific survival by baseline platelet count category, for ovarian cancer patients. (**a**) Ovary (stage I/II patients) (*n* = 651); (**b**) ovary (stage III/IV patients) (*n* = 1425).

We sought to determine whether platelet counts were associated with cancer survival, independently of stage. After adjusting for patient variables (age, sex, year of diagnosis, common chronic conditions) and cancer stage, the associations of platelet count and cancer survival were attenuated but remained significant (except for stomach cancer) (Table 2). Moreover, platelet counts were associated with mortality among patients with early-stage disease (Table S13). Among stage I/II colon cancer patients, cancer-specific mortality was higher among patients with a high platelet count relative to a medium platelet count (aHR 1.64; 95%CI 1.39–1.93). For stage I/II ovarian cancer patients, cancer-specific mortality was much higher among patients with a high platelet count than patients with a medium platelet count (aHR 3.10; 95%CI 1.74–5.52). Among stage I/II ovarian cancer patients, the 5-year survival for patients with a low/medium platelet count (<75th percentile) and a high platelet count (≥75th percentile) was 91% and 71%, respectively (Figure 2).

### 3.2. Time-Dependent Platelet Count Analysis

In our time-dependent analysis, we examined the association of platelet counts measured during the follow-up period and subsequent cancer survival. The median number of CBC tests performed in the follow-up period was 8 per patient (interquartile range 3–20). The association of time-dependent platelet count and cancer survival was consistent with what was observed with baseline platelet count (Tables S14–S16). As in the primary analysis, a higher platelet count was associated with worse survival among patients with colon, lung, ovarian, and stomach cancers. For breast and prostate cancers, both high and low platelet count categories were associated with worse cancer survival.

**Figure 3:**
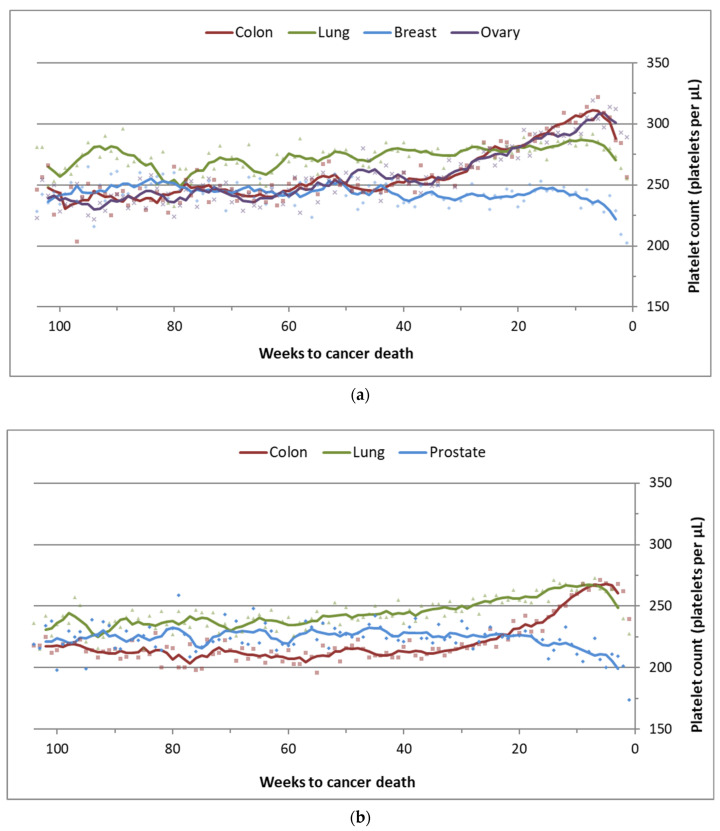
Median platelet count moving average (period of 5) in the 104 weeks (2 years) preceding death, among patients with a cancer-specific death. (**a**) Female patients; (**b**) male patients.

In a non-parametric analysis, we report the moving average for median platelet count in the two years preceding death among patients that died from their index cancer (Figure 3). We observed a temporal increase in median platelet count in colon, lung, and ovarian cancer patients in the 6 months prior to death. Immediately preceding death there was a sharp decline in median platelet count for all patients. Conversely, median platelet count was stable in the two years preceding the end of follow-up among patients who did not die from their index cancer (i.e., were censored or died from another cause) (Figure S6). We also looked at median platelet count for patients who did not die of their cancer and had a minimum follow up of 5 years (Figure S7). Median platelet count dropped in the first year after diagnosis and remained stable between the years 2 and 5.

## 4. Discussion

In this population-level retrospective cohort study, we found an association between platelet count (both at the time of a cancer diagnosis and following diagnosis) and cancer survival across a wide range of solid cancer types. A platelet count in the high category (≥75th percentile) was associated with increased cancer-specific death among patients with colon, lung, ovarian, and stomach cancers. 

Our findings are consistent with previous epidemiologic studies on platelet count and cancer survival [10,11,12,13,14,15,16,17,18,19,20,21,22,23]. Most earlier studies reported on cancer at a single site using thrombocytosis as the exposure. We demonstrated, in our very large series, that three categories of platelet count (low, medium, high) are sufficient to stratify patients by risk for many cancer types. A finer resolution of platelet category into five levels was slightly more discriminating.

Our findings suggest that platelet counts taken following cancer diagnosis continue to be associated with survival for a wide range of cancer types. For colon, lung, and ovarian cancers, platelet counts rose in the 6-month period prior to death. In contrast, for colon, lung, and ovarian cancer patients who did not die from their index cancer, platelet count dropped in the year following diagnosis and remained stable thereafter. These temporal associations suggest that platelet count may be a marker for the presence of cancer, both at the time of diagnosis and in the follow-up period.

We also showed that baseline platelet count is associated with cancer survival for patients diagnosed with early-stage cancer. Among patients with early-stage ovarian cancer (stages I and II) 5-year cancer-specific survival rates for patients with low/medium and high baseline platelet counts were 91% and 71%, respectively (Figure 2). It is possible that a high baseline platelet count is indicative of microscopic residual disease among patients with early-stage ovarian cancer (stage I/II).

Stone et al. performed a prospective cohort study on 619 patients with epithelial ovarian cancer and observed that an elevated platelet count resulted in poor survival [22]. They hypothesized that pro-inflammatory cytokines produced by the tumor resulted in paraneoplastic thrombocytosis, which enhanced tumor growth. It is likely that the inflammatory cytokine milieu, which is associated with several malignancies, promotes thrombopoiesis in cancer patients. Moreover, the release of pro-inflammatory cytokines by cancer cells resulted in the upregulation of thrombopoietin in the liver thereby inducing megakaryopoiesis and increasing platelet count. As such, thrombocytosis may represent a marker for the burden of malignancy. The mechanisms by which platelets and cancer prognosis are related are not completely known, although it is proposed that it may be bidirectional, i.e., cancers may increase platelet production (or decrease clearance) as a paraneoplastic syndrome and activated platelet functions may enhance cancer progression through release of cytokines and chemokines, by aiding extravasation of cancer cells from vessels in the metastatic niche and by putting up an immune defense scaffolding [1,2,3]. In animal models, a reduced platelet count can delay progression in some neoplastic processes [28]. Moreover, it has been suggested that anti-platelet drugs, such as aspirin, may have a therapeutic effect in some solid tumors but progress to date has been limited [29,30,31].

We observed worse cancer-specific survival among breast and prostate cancer patients with a low platelet count. Prostate cancer patients with a low platelet count did as poorly as patients with a high platelet count (Figure 1e). It is unclear why for these cancers there is a U-shaped association with platelet count and cancer-specific death whereas for other cancer sites, the association is linear. Breast and prostate cancer patients are prone to bone metastasis, and this may impact on megakaryocyte production of platelets. If this were to be the case, we would expect positive associations with very low platelet count to be present among other cancer types that tend to metastasize to bone. Alternatively, a very low platelet count could be due to medical conditions which in turn could increase the mortality rate in these patients.

### Strengths and Limitations

To our knowledge, this is the largest and most comprehensive study on the association of platelet count and cancer survival. Our cohort consisted of 112,231 individuals in Ontario diagnosed with a solid tumor between 2007 and 2016 and an available CBC record. This large sample size allowed us to conduct a detailed study on multiple cancer types at various stages and at varying levels of platelet count.

Our study did have limitations. Our cohort consisted of cancer patients with a CBC record in the 30 days prior to diagnosis. Of the 500,386 cancer patients that met all other eligibility criteria, only 112,231 (22.4%) had a baseline platelet count. This is largely because the catchment area of the OLIS dataset grew by calendar year. However, given the high rate of exclusion there is the opportunity for selection bias. Patients with and without a baseline platelet count were similar with respect to their demographic information, health services utilization, comorbidities, and medication use (Table S7). The OLIS dataset began in 2007 and initially included a small catchment area in Ontario, expanding to approximately 61% of the Ontario population by 2017. A study by Iskander et al. reported no meaningful differences in demographic and comorbidity profiles of Ontarians residing in the 2007 versus 2017 catchment areas of OLIS [32]. Thus, cancer patients with a baseline CBC record identified in the early years of the accrual period are likely to be similar to patients in the latter years of the accrual period.

## 5. Conclusions

A higher platelet count is associated with cancer-specific death for many common cancer sites. The results suggest that platelet count might be an indicator of residual disease post-treatment and could potentially be used as a risk stratification measure for intensified treatment. 

## Figures and Tables

**Table 1:** Descriptive table of eligible subjects, measured at the cancer diagnosis date.

Description	Value	Total
Overall		112,231
General demographics		
Calendar year	Mean (SD)	2013.2 (2.4)
	Median (IQR)	2014 (2011–2015)
Sex	Female	56,524 (50.4%)
	Male	55,707 (49.6%)
Age	Mean (SD)	66.9 (14.3)
	Median (IQR)	68.0 (57.8–77.5)
Neighborhood income quintile	1—Low	22,374 (19.9%)
	2	23,340 (20.8%)
	3	22,225 (19.8%)
	4	21,812 (19.4%)
	5—High	22,171 (19.8%)
	Missing	309 (0.3%)
Residence location	Urban	97,523 (86.9%)
	Rural	14,571 (13.0%)
	Missing	137 (0.1%)
Landed immigrant	Non-immigrant	99,372 (88.5%)
	Recent immigrant (≤10 years)	3135 (2.8%)
	Past immigrant (>10 years)	9724 (8.7%)
Time eligible in OHIP (years)	Mean (SD)	21.1 (5.8)
	Median (IQR)	22.9 (19.6–25.0)
Health service utilization		
Core primary care visits to GP/FP (2 years prior)	Mean (SD)	2.9 (3.5)
	Median (IQR)	2 (1–4)
	0	4750 (4.2%)
	1–2	61,661 (54.9%)
	3–4	24,009 (21.4%)
	5–9	16,980 (15.1%)
	10+	4831 (4.3%)
Chronic conditions		
Asthma	Yes	10,397 (9.3%)
Congestive heart failure	Yes	9509 (8.5%)
Inflammatory bowel disease	Yes	435 (0.4%)
Chronic obstructive pulmonary disease	Yes	11,578 (10.3%)
HIV	Yes	171 (0.2%)
Hypertension	Yes	63,988 (57.0%)
Dementia	Yes	4807 (4.3%)
Diabetes	Yes	23,222 (20.7%)
Chronic rheumatoid arthritis	Yes	1464 (1.3%)
Osteoarthritis	Yes	20,042 (17.9%)
Mood disorder	Yes	13,807 (12.3%)
Other mental health disorder	Yes	6629 (5.9%)
Osteoporosis	Yes	1831 (1.6%)
Renal disease	Yes	6303 (5.6%)
Stroke	Yes	2486 (2.2%)
Chronic coronary syndrome	Yes	12,131 (10.8%)
Acute myocardial infarction	Yes	2830 (2.5%)
Medication use (patients aged 66+)		
Concurrent medication use		
Number of concurrent medications	Mean (SD)	4.6 (3.3)
	Median (IQR)	4 (2–7)
Recent medication use		
Antiplatelet		
Nonsteroidal anti-inflammatory (ASA-based)	Yes	2062 (3.3%)
Nonsteroidal anti-inflammatory (non-ASA-based)	Yes	11,481 (18.4%)
Adenosine disphosphonate inhibitor	Yes	4772 (7.7%)
Cardiovascular		
Coronary vasodilator (nitrate)	Yes	5415 (8.7%)
Beta blocker	Yes	18,561 (29.8%)
Calcium channel blocker	Yes	19,725 (31.7%)
ACE inhibitor	Yes	19,838 (31.9%)
Angiotensin receptor agonist	Yes	14,684 (23.6%)
Lipid-lowering		
Statin	Yes	33,080 (53.1%)
Psychotropics		
Tricyclic antidepressant	Yes	2975 (4.8%)
Selective serotonin reuptake inhibitor	Yes	7311 (11.7%)
Incident cancer events (Ontario Cancer Registry)		
Lung	Yes	20,583 (18.3%)
Colon	Yes	17,259 (15.4%)
Breast	Yes	9857 (8.8%)
Prostate	Yes	8587 (7.7%)
Other solid tumor	Yes	8051 (7.2%)
Bladder	Yes	6963 (6.2%)
Thyroid	Yes	5944 (5.3%)
Kidney	Yes	5272 (4.7%)
Pancreas	Yes	5195 (4.6%)
Endometrium	Yes	4500 (4.0%)
Stomach	Yes	3365 (3.0%)
Head and neck	Yes	3160 (2.8%)
Ovary	Yes	3085 (2.7%)
Melanoma	Yes	2815 (2.5%)
Brain	Yes	2563 (2.3%)
Other GI	Yes	1726 (1.5%)
Esophagus	Yes	1483 (1.3%)
Testis	Yes	990 (0.9%)
Cervix	Yes	833 (0.7%)
Complete blood count		
Number of baseline CBC observations	1	83,819 (74.7%)
	2	18,559 (16.5%)
	3+	9853 (8.8%)
Platelet count [10^9^ platelets/L]	Mean (SD)	275.1 (103.6)
	Median (IQR)	256 (208–320)
Number of CBC observations in the follow-up period	Mean (SD)	15.0 (20.3)
	Median (IQR)	8 (3–20)
Follow-up period		
Follow-up time (years)	Mean (SD)	2.6 (2.4)
	Median (IQR)	1.9 (0.7–3.9)
Any death	Yes	51,738 (46.1%)
Any cancer death	Yes	41,968 (37.4%)
Cancer-specific death	Yes	40,329 (35.9%)

**Table 2:** Cancer-specific mortality rates and hazard ratios by baseline platelet count category for select cancers.

Cancer	Platelet Category	Person-Years	Cancer-Specific Deaths	Rate (% per Year)	Unadjusted	*p*-Value	Patient Variable Adjustment *		Patient Variable and Cancer Stage Adjustment **	
					HR (95% CI)		HR (95% CI)	*p*-Value	HR (95% CI)	*p*-Value
All cancers	1—Low (≤25th pctl)	76,425.8	8666	11.3	0.91 (0.88–0.93)	<0.0001	0.84 (0.82–0.86)	<0.0001	0.91 (0.88–0.94)	<0.0001
	2—Medium (>25 to <75th pctl)	153,022.0	18,845	12.3	1.00 (Reference)		1.00 (Reference)		1.00 (Reference)	
	3—High (≥75th pctl)	65,811.5	12,818	19.5	1.52 (1.48–1.55)	<0.0001	1.55 (1.52–1.59)	<0.0001	1.23 (1.20–1.26)	<0.0001
Colon	1—Low (≤25th pctl)	13,656.9	926	6.8	0.70 (0.65–0.76)	<0.0001	0.69 (0.64–0.75)	<0.0001	0.92 (0.84–0.99)	0.0334
	2—Medium (>25 to <75th pctl)	26,021.7	2510	9.6	1.00 (Reference)		1.00 (Reference)		1.00 (Reference)	
	3—High (≥75th pctl)	10,913.0	1884	17.3	1.70 (1.60–1.80)	<0.0001	1.71 (1.61–1.81)	<0.0001	1.32 (1.24–1.40)	<0.0001
Lung	1—Low (≤25th pctl)	8415.3	2798	33.2	0.80 (0.77–0.84)	<0.0001	0.75 (0.72–0.79)	<0.0001	0.93 (0.89–0.97)	0.0024
	2—Medium (>25 to <75th pctl)	14,959.1	6504	43.5	1.00 (Reference)		1.00 (Reference)		1.00 (Reference)	
	3—High (≥75th pctl)	4977.9	3872	77.8	1.55 (1.48–1.61)	<0.0001	1.57 (1.51–1.63)	<0.0001	1.35 (1.29–1.40)	<0.0001
Breast	1—Low (≤25th pctl)	9436.4	298	3.2	1.19 (1.03–1.37)	0.0193	1.05 (0.91–1.21)	0.5042	1.11 (0.96–1.30)	0.1654
	2—Medium (>25 to <75th pctl)	19,744.7	516	2.6	1.00 (Reference)		1.00 (Reference)		1.00 (Reference)	
	3—High (≥75th pctl)	9377.9	428	4.6	1.73 (1.52–1.97)	<0.0001	1.80 (1.58–2.04)	<0.0001	1.23 (1.07–1.41)	0.0028
Prostate	1—Low (≤25th pctl)	7499.3	365	4.9	1.66 (1.45−1.90)	<0.0001	1.39 (1.21–1.60)	<0.0001	1.30 (1.11–1.51)	0.0009
	2—Medium (>25 to <75th pctl)	16,960.5	474	2.8	1.00 (Reference)		1.00 (Reference)		1.00 (Reference)	
	3—High (≥75th pctl)	8034.9	434	5.4	1.91 (1.68–2.17)	<0.0001	1.87 (1.64–2.13)	<0.0001	1.39 (1.21–1.61)	<0.0001
Ovary	1—Low (≤25th pctl)	2161.7	226	10.5	0.65 (0.56–0.76)	<0.0001	0.63 (0.54–0.74)	<0.0001	0.78 (0.64–0.95)	0.0143
	2—Medium (>25 to <75th pctl)	4017.9	650	16.2	1.00 (Reference)		1.00 (Reference)		1.00 (Reference)	
	3—High (≥75th pctl)	1699.2	459	27.0	1.60 (1.42–1.80)	<0.0001	1.60 (1.42–1.81)	<0.0001	1.27 (1.10–1.48)	0.0016
Stomach	1—Low (≤25th pctl)	1441.4	478	33.2	0.88 (0.79–0.99)	0.0265	0.87 (0.78–0.97)	0.0111	1.08 (0.91–1.28)	0.3949
	2—Medium (>25 to <75th pctl)	2745.4	1035	37.7	1.00 (Reference)		1.00 (Reference)		1.00 (Reference)	
	3—High (≥75th pctl)	1191.1	574	48.2	1.25 (1.13–1.38)	<0.0001	1.23 (1.11–1.36)	0.0001	1.03 (0.88–1.21)	0.6992

HR = hazard ratio; pctl = percentile; NA = not applicable. * Adjusted for age at diagnosis (cat.), year of diagnosis (cont.), and common chronic conditions (asthma, congestive heart failure, chronic obstructive pulmonary disease, hypertension, and diabetes). ** Adjusted for age at diagnosis (cat.), year of diagnosis (cont.), common chronic conditions (asthma, congestive heart failure, chronic obstructive pulmonary disease, hypertension, and diabetes), and cancer stage. Only subjects with known cancer stage were included.

## Data Availability

All data generated or analyzed during this study are included in this published article and its supplementary material.

## Supplementary Materials

The following supporting information can be downloaded at: https://www.mdpi.com/article/10.3390/cancers14030549/s1, Figure S1: Study design and inclusion criteria among cancer patients; Figure S2: Proportion of cancer patients with a baseline platelet count (within 30 days of cancer diagnosis), by calendar year; Figure S3: Schematic of time-dependent confounding that could occur between platelet count and cancer treatment; Figure S4: Distribution of stabilized weights among observations included in the marginal structural models (exposure definition 1); Figure S5: Cancer-specific survival by baseline platelet count category (exposure definition 2), for select cancers; Figure S6: Median platelet count moving average (period of 5) in the 104 weeks (2 years) preceding end of follow-up, among patients that did not die from their index cancer; Figure S7: Median platelet count moving average (period of 7) in the 260 weeks (5 years) following a cancer diagnosis, among patients with at least 5 years of follow-up and did not die from their index cancer; Table S1: Description of administrative datasets used; Table S2: Definitions of site-specific cancer events; Table S3: Definitions of descriptive and treatment variables; Table S4: Definitions of cancer-specific death; Table S5: Inclusion table for cancer subjects; Table S6: Exclusion table for study cohort; Table S7: Descriptive table of cancer patients (meeting all exclusion criteria) with versus without a baseline platelet count; Table S8: Descriptive table of cancer patients by baseline platelet count category; Table S9: Sex- and cancer-specific platelet count reference distributions for exposure definition; Table S10: Distribution of aggregate cancer stage by cancer site; Table S11: Cancer-specific mortality rates and hazard ratios by baseline platelet count category (exposure definition 1), for all cancers; Table S12: Cancer-specific mortality rates and hazard ratios by baseline platelet count category (exposure definition 2), for all cancers; Table S13: Cancer-specific mortality rates and hazard ratios by baseline platelet count category, by cancer site and stage; Table S14: Cancer-specific mortality rates and hazard ratios by time-dependent platelet count category, for select cancers; Table S15: Cancer-specific mortality rates and hazard ratios by time-dependent platelet count category (exposure definition 1); Table S16: Cancer-specific mortality rates and hazard ratios by time-dependent platelet count category (exposure definition 2).
