# Supplementary material for: Platelet Count and Survival after Cancer
Source: Cancers (Basel). 2022 Jan 21;14(3):549. doi: 10.3390/cancers14030549 (PMC8833779; doi:10.3390/cancers14030549)
Supplement: Supplementary file 1 [file cancers-14-00549-s001.zip › cancers-1547094-supplementary.pdf]

cancers

MDPI

Supplementary Material

Platelet Count and Survival after Cancer

Vasily Giannakeas, Joanne Kotsopoulos, Jennifer D. Brooks, Matthew C. Cheung, Laura Rosella, Lorraine Lipscombe, Mohammad R. Akbari, Peter C. Austin and Steven A. Narod

Table S1. Description of administrative datasets used.

Datasets	Years used	Description of use
	Population and demographics	
Client Agency Program Enrollment (CAPE)	1999–2016	The CAPE dataset indicates an Ontario resident's enrollment to a primary care/ family doctor along with a start and end date of the enrollment.<br><br>The CAPE dataset was used to determine what individuals in the cohort were rostered to a primary care physician on the index date.
Immigration, Refugees and Citizenship Canada Permanent Resident Database (CIC)	1985–2016	The CIC dataset includes immigration application records for individuals in Ontario.<br><br>The CIC dataset was used to capture immigration status among individuals in the cohort. The landing date was used to further classify an individual as a recent immigrant (≤ 10 years from index date) or past immigrant (>10 years from index date).
Office of the Registrar General – Deaths (ORGD)	2007–2017	The ORGD dataset contains cause of death information for Ontario residents.<br><br>The ORGD dataset was used to derive cancer-specific or non-specific death for individuals in the cohort that had a death date in the follow-up period. The underlying cause of death variable, along with ICD-9 codes and the index cancer type were used to derive this variable.
Surname-based Ethnicity Group Database (ETHNIC)	1990–2016	The ETHNIC database is a surname-based algorithm which classifies Ontario residents as either General Population, Chinese, or South Asian[1].
Registered Persons Database (RPDB)	1991–2017	The RPDB dataset provides basic demographic information on all Ontario residents registered with provincial health coverage. Demographic variables in the RPDB include date of birth, death date, sex, and postal code (updated annually). Start and end dates of provincial health coverage enrollment are also provided.<br><br>The RPDB dataset was used to get an individual's age (using date of birth) and sex. Neighborhood income quintile and residence location were derived from postal code information. Start and end dates for provincial health coverage (OHIP) and death date were used to determine eligibility.
	Health services	
Discharge Abstract Database (DAD)	2002–2017	The DAD dataset includes patient-level data for acute care hospitalizations. The data includes clinical details for each hospitalization such as clinical diagnosis (ICD-10) and health-related interventions (CCI).

		Data from DAD was used to capture urgent inpatient hospitalization episodes, Charlson comorbidities, and historic documentation of diseases. Data from DAD was also used to identify chemotherapy or radiotherapy that occurs in an inpatient setting.
National Ambulatory Care Reporting System Database (NACRS)	2002–2017	The NACRS dataset includes visits to an emergency department in Ontario. The data includes details on clinical diagnosis (ICD-10) and health-related interventions (CCI).<br><br>Data from NACRS was used to capture unscheduled emergency department visits. The NACRS dataset also contains cancer clinic visits, which were used to identify chemotherapy and radiotherapy use.
Ontario Drug Benefit Claims Database (ODB)	2005–2016	The ODB dataset includes dispensed medication claims among individuals enrolled in the ODB program in Ontario. All Ontario residents 65 and older are eligible for the ODB program.<br><br>Data from the ODB was used to determine what proportion of individuals are active in the program (i.e. have more than one claim in the year prior), capture low-income seniors, as well as capture concurrent and historic medication use.
Ontario Health Insurance Plan Claims (OHIP)	1997–2017	The OHIP dataset includes physician billings to the province of Ontario among residents with provincial health coverage. Each billing includes a data element on the physician ID, the physician specialty, the patient ID, a diagnostic code (ICD-9), a fee code, and the service date.<br><br>Data from the OHIP dataset was used to obtain variables for primary care visits, specialist visits, cancer screening, cancer treatment, comorbidities, and chronic conditions.
Ontario Laboratories Information System (OLIS)	2007–2017	The OLIS dataset includes laboratory test results among tests being conducted in Ontario.<br><br>The data was used to acquire a dataset of complete blood count (CBC) tests among Ontario residents in the accrual period. These CBC tests were used for exclusions and the exposure definition.
Same Day Surgery (SDS)	2002–2017	The SDS dataset contains patient-level information on same day surgeries, with a similar format to the DAD data.<br><br>Data from the SDS data was used to assign Charlson comorbidities, chemotherapy and radiotherapy.
	Registries	
Cancer Activity Level Reporting (ALR)	2005–2017	The ALR dataset includes patient-level activity among cancer patients.<br><br>The dataset was used to determine chemotherapy and radiotherapy treatment among eligible subjects.
New Drug Funding Program (NDFP)	2005–2017	The NDFP data contains dispensation records of new oncologic medications that are typically not funded under standard provincial health coverage.<br><br>The dataset was used to determine chemotherapy and radiotherapy treatment among eligible subjects.
Ontario Breast Screening Program (OBSP)	2005–2016	The OBSP data contains breast screening data among women enrolled in the OBSP program. The data was used to determine if a woman was up to date on her breast cancer screening.

Ontario Cancer Registry (OCR)	1964–2016	The OCR dataset includes all reportable incident cancer diagnosis in Ontario between January 1964 and September 2019[2]. Each incident cancer diagnosis contains information on the cancer site/topology (ICD-10) and cancer morphology (ICD-O-3).<br><br>The OCR data was used to create our inclusion cohort; individuals diagnosed with a cancer in the accrual period. The data was also used to capture cancer-specific variables such as cancer topology, histology and stage.
ICES derived cohorts		
Ontario Asthma Dataset (ASTHMA)	1993–2016	A population-level cohort of Ontario residents with asthma.<br><br>A validated algorithm using DAD and OHIP data[3]:<br>Sensitivity = 80.6%<br>Specificity = 81.4%<br>PPV = 72.5%<br>NVP = 87.3%
Congestive Heart Failure (CHF)	1991–2016	A population-level cohort of Ontario residents with congestive heart failure.<br><br>A validated algorithm using DAD, NACRS and OHIP data[4]:<br>Sensitivity = 84.8%<br>Specificity = 97.0%<br>PPV = 55.6%
Chronic Obstructive Pulmonary Disease (COPD)	1991–2016	A population-level cohort of Ontario residents with chronic obstructive pulmonary disease.<br><br>Validated algorithm using DAD and OHIP data[5]:<br>Sensitivity = 85.0%<br>Specificity = 78.4%
Ontario Dementia Database (DEMENTIA)	1996–2016	A population-level cohort of Ontario residents with dementia.<br><br>Validated algorithm using DAD, SDS, ODB and OHIP data[6]:<br>Sensitivity = 79.3%<br>Specificity = 99.1%
Ontario HIV Database (HIV)	1991–2016	A population-level cohort of Ontario residents with HIV.<br><br>Validated algorithm using OHIP data[7]:<br>Sensitivity = 96.2%<br>Specificity = 99.6%
Ontario Hypertension dataset (HYPER)	1991–2016	A population-level cohort of Ontario residents with hypertension.<br><br>Validated algorithm using DAD and OHIP data[8]:<br>Sensitivity = 72%<br>Specificity = 95%<br>PPV = 87%<br>NVP = 88%
Ontario Mother-Baby Linked Dataset (MOMBABY)	1988–2016	The MOMBABY dataset includes infant deliveries in an inpatient setting beginning in 1988. The MOMBABY dataset was used to capture the number of previous infant deliveries and/or any recent infant deliveries (in the 2 years prior) among female individuals.
Ontario Crohn's and Colitis Cohort Dataset (OCCC)	1991–2016	A population-level cohort of Ontario residents with Crohn's and Colitis.<br><br>Validated algorithm using DAD, NACRS and OHIP data[9]:<br>Sensitivity = 59.3%

		Specificity = 99.0%<br>PPV = 71.1%<br>NVP = 98.3%
Ontario Diabetes Dataset (ODD)	1991–2016	A population-level cohort of Ontario residents with diabetes.<br><br>Validated algorithm using DAD and ODB data[10]:<br>Sensitivity = 90.0%<br>Specificity = 97.7%<br>PPV = 92.6%
Ontario Rheumatoid Arthritis Dataset (ORAD)	1993–2016	A population-level cohort of Ontario residents with rheumatoid arthritis.<br><br>Validated algorithm using DAD and OHIP data[11]:<br>Sensitivity = 78%<br>Specificity = 100%<br>PPV = 78%<br>NVP = 100%
Other datasets		
Drug List Dataset (DIN)	1990–2020	The DIN dataset includes drug information unique by the drug DIN. Drug data includes drug name, drug class, strength, and route administered.<br><br>Unique drug name was used to determine the concurrent number of drugs being used. Drug class was used to identify historic use of drugs that affect platelet function.

ICD = International Classification of Disease; CCI = Canadian Classification of Health Interventions; PPV = Positive Predictive Value; NPV = Negative Predictive Value; ODB = Ontario Drug Benefits Database.

Table S2. Definitions of site-specific cancer events.

Definition	Sub-definition	Data source(s)	Code type	Codes	Conditions/Notes
Incident cancer diagnosis	Any malignant neoplasm	OCR	ICD-10	C00-C97	
*Malignant solid tumour ** *	Any primary solid tumour	OCR	ICD-10	C00-C97	
	Colon	OCR	ICD-10	C18 – Colon<br>C19 – Rectosigmoid junction<br>C20 – Rectum	
	Lung	OCR	ICD-10	C34 – Bronchus or lung	
	Breast	OCR	ICD-10	C50 – Breast	Female sex
	Ovary	OCR	ICD-10	C56 – Ovary<br>C570 – Fallopian tube	Female sex
	Cervical	OCR	ICD-10	C53 – Cervix	Female sex
	Endometrium	OCR	ICD-10	C54 – Corpus uteri	Female sex
	Prostate	OCR	ICD-10	C61 – Prostate	Male sex
	Testicle	OCR	ICD-10	C62 – Testis	Male sex
	Thyroid	OCR	ICD-10	C73 – Thyroid gland	
	Pancreas	OCR	ICD-10	C25 – Pancreas	
	Stomach	OCR	ICD-10	C16 – Stomach	
	Kidney	OCR	ICD-10	C64 – Kidney<br>C65 – Renal Pelvis	
	Bladder	OCR	ICD-10	C67 – Bladder	
	Liver	OCR	ICD-10	C22 – Liver and intrahepatic bile ducts	
	Esophagus	OCR	ICD-10	C15 – Esophagus	
	Other gastrointestinal tract	OCR	ICD-10	C17 – Small intestine<br>C21 – Anus and anal canal<br>C23 – Gallbladder	
	Brain	OCR	ICD-10	C71 – Brain	
	Melanoma	OCR	ICD-10	C00-C97, **and one of**	
			ICD-O-3	8720/3 – Malignant Melanoma, NOS<br>8743/3 – Superficial Spreading Melanoma<br>8721/3 – Nodular Melanoma<br>8742/3 – Lentigo Maligna Melanoma<br>8744/3 – Acral Lentiginous Melanoma<br>8772/3 – Spindle Cell Melanoma, NOS	Note: All ICD-O-3 melanoma codes were derived from the cancer registry and are in order from most frequent to least frequent among melanoma pathologies.

			8745/3 – Desmoplastic Melanoma<br>8770/3 – Mixed Epithelioid and Spindle Cell Melanoma<br>8771/3 – Epithelioid Cell Melanoma<br>8730/3 – Amelanotic Melanoma<br>8723/3 – Malignant Melanoma, Regressing<br>8774/3 – Spindle Cell Melanoma, Type B<br>8761/3 – Malignant Melanoma in a Giant Pigmented Nevus<br>8740/3 – Malignant Melanoma in Junctional Nevus<br>8722/3 – Balloon Cell Melanoma<br>8773/3 – Spindle Cell Melanoma, type A<br>8746/3 – Mucosal Lentiginous Melanoma<br>8741/3 – Malignant Melanoma in Precancerous Melanosis	
Head and neck	OCR	ICD-10	C00-C09 – Lip, oral cavity<br>C10 – Oropharynx<br>C11 – Nasopharynx<br>C13 – Hypopharynx<br>C12 – Piriform sinus<br>C14 – Lip, oral cavity and pharynx (ill-defined)<br>C30 – Nasal cavity and middle ear<br>C31 – Accessory sinuses<br>C32 – Larynx	
Other solid tumour	OCR	ICD-10	C00-C97	Cancer event met solid tumour definition, but none of the site-specific definitions described above.

** Solid tumours are morphologic codes that are not in hematological, lymphoid and related tissue definitions.

Table S3. Definitions of descriptive and treatment variables.

Variable Class	Definition	Data source(s)	Code type	Codes	Conditions/Notes
Health services utilization	Core primary care visit	OHIP	Fee code	A001 - MINOR ASSESS.-F.P./G.P.<br>A002 - Family Practice & Practice in General - Enhanced 18 month well baby visit<br>A003 - GEN. ASSESS. -F.P./G.P.<br>A004 - GEN.RE-ASSESS-F.P./G.P.<br>A005 - CONSULTATION -F.P./G.P.<br>A007 - INTERMED.ASSESS/WELL BABY CARE-F.P./G.P./PAED.<br>A008 - MINI ASSESSMENT-F.P./G.P.<br>A903 - GEN/FAM PRACT-PRE-DENTAL/OPER.ASSESS LIMIT 2 PER YEAR/PT<br>E075 - GERIATRIC GENERAL ASSESSMENT PREMIUM<br>G212 - D./T. PROC.-ALLERGY-HYPOSENSITIZATION INJECTION PLUS BASIC<br>G271 - D./T. PROC.-CARDIOV.-ANTICOAGULANT SUPERVISION<br>G372 - D./T. PROC.-INJECTIONS-INTRADERMAL/MUSCULAR ETC. EA. ADD.<br>G373 - D./T. PROC.-INJ. INTRADERMAL/MUSC. BASIC FEE (SHICK TEST)<br>G365 - D./T. PROC.-GYNAECOLOGY-PAPANICOLAOU SMEAR<br>G538 - D&T IMMUNIZATION-WITH VISIT, EACH INJECT.<br>G539 - Injection of unspecified agent - sole reason (first injection)<br>G590 - INFLUENZA AGENT +VISIT<br>G591 - Injection of influenza agent - sole reason<br>K005 - INDIVIDUAL CARE PER 1/2 HR<br>K013 - COUNSELLING-ONE OR MORE PEOPLE-PER 1/2HR.<br>K017 - ANNUAL HEALTH EXAM-CHILD AFT. 2ND BIRTHDAY.<br>P004 - OBS.-PRENATAL CARE-MINOR PRENATAL ASSESS.-SUBSEQ.PRENAT.VIS.<br>K130 - Periodic health visit - adolescent<br>K131 - Periodic health visit - adult aged 18 to 64 inclusive<br>K132 - Periodic health visit - adult 65 years of age and older<br>K030 - DIABETIC MANAGEMENT FEE	Note: All core primary care visits were restricted to OHIP billings from primary care providers (GP and community medicine specialties). A maximum of one core visit per patient-day-physician was counted.
	Specialist visit	OHIP	Specialty code	02 – Dermatologist<br>03 – General surgeon<br>06 – Orthopedic surgeon	

			07 – Geriatrician<br>13 – Internal medicine specialist<br>15 – Endocrinologist<br>16 – Nephrologist<br>18 – Neurologist<br>19 – Psychiatrist<br>20 – OBGYN<br>22 – Geneticist<br>24 – ENT<br>35 – Urologist<br>41 – Gastroenterologist<br>44 – Oncologist<br>47 – Respirologist<br>48 – Rheumatologist<br>60 – Cardiologist<br>61 – Hematologist
Breast cancer screen	OHIP	Fee code	X172 – Mammogram - No Signs or Symptoms - unilateral<br>X178 – Mammogram - No Signs or Symptoms - bilateral<br>X185 – DIAGNOSTIC RADIOLOGY-MAMMOGRAM-BILATERAL
	OBSP		Screened variable in (“2” “3”)
Cervical cancer screen	OHIP	Fee code	E430 – D/T PROC-PAP SMEAR PERFORMED OUTSIDE OF HOSP-ADD<br>G365 – D./T. PROC.-GYNAECOLOGY-PAPANICOLAOU SMEAR<br>E431 – when papanicolaou smear is performed outside of hospital, to G394<br>G394 – ADD'L PAP SMEAR FOR FOLLOWUP OF ABNORM OR INADEQ SMEARS<br>L713 – LAB.MED.-ANAT PATH,HIST,CYT-CYTOL-GYNAECOLOGICAL SPECIMEN<br>L733 – CERVICOVAGINAL SPECIMEN (MONOLAYER CELL METHODOLOGY)<br>L812 – LAB MED.CYTOLOGY-CERVICOVAG SPEC<br>Q678 – GYNAECOLOGY - PAPANICOLAOU SMEAR- PERIODIC - N.P.
Colon cancer screen			G004 – D./T. PROC-LAB.MED.-OCCULT.BLOOD.<br>L181 – LAB. MED.-BIOCHEM.-OCCULT BLOOD<br>L179 – TRACKING CODE-COLON CANCER CHECK<br>Q152 – FOBT COMPLETION FEE

				Q043 – NEW PATIENT FEE FOBT POSITIVE/CRC INCREASED RISK<br><br>Z535 – INTESTINES-ENDOSCOPY-SIGMOIDOSCOPY W/WITHOUT ANOSCOPY<br>Z555 – INTESTINES-ENDOSCOPY-COLONOSCOPY INTO DESCENDING COLON<br>Z580 – INTESTINE-ENDOSCOPY-USING 60C.M. FLEXIBLE ENDOSCOPE.<br><br>E740 – INTESTINE ENDO SIGMOID TO SPLENIC FLEXURE ADD<br>E741 – INTESTINE END SIGMOID TO HEPATIC FLEXURE ADD<br>E747 – INTESTINE-ENDOSCOPY-SIGMOID.TO CAECUM ADD TO Z512/Z555<br>E705 – DIGEST.SYST.INTEST.ENDOSC.INTO TERMINAL ILEUM,ADD.<br>Z492 – Five year follow up of normal colonoscopy Z499, absence of intervening signs or symptoms<br>Z493 – Ten year follow up of normal colonoscopy (Z497), absence of intervening signs or symptoms<br>Z497 – Confirmatory colonoscopy - sigmoid to descending colon<br>Z498 – Surveillance colonoscopy - sigmoid to descending colon<br>Z499 – Colonoscopy - Absence of signs or symptoms, family history of colon cancer in a first degree relative
Cancer treatment	Chemotherapy	OHIP	Fee code	G261 – D./T. PROC.INJ/INF-INTRAVEN-CHEMOTHERAPY-EA.ADD.INJ.TO G381<br>G339 – Chemotherapy - Single agent intravenous chemotherapy i.e. doxorubicin, daunorubicin, epirubicin, mitoxintrone, cisplatin or bleomycin (greater than 10 units per metre square)<br>G345 – D&T MULT.AGENTS CHEMOTHER.GREATER THAN 10 UNITS PER MET.SQ.<br>G359 – D&T SING.AGENT CHEMOTHER.(GREATER THAN 2G/M2 OR 1G/M2.)<br>G381 – D./T. PROC. INJECT/INFUS. INTRAVENOUS CHEMOTHERAPY-1ST INJ<br>G382 – D./T. PROC.-INJ./INFUSION-SUPERVISION CHEMOTHERAPY BY PHONE<br>G388 – Management of special oral chemotherapy, for malignant disease

	DAD/SDS/NACRSICD-10		Z511 – Chemotherapy session for neoplasm<br>Z512 – Other chemotherapy
	NDFP		Any treatment records
	ALR (systemic)		Any treatment records
Radiotherapy	OHIP	Fee code	*External beam radiotherapy*<br>X302 – THERA. RADIOL.-TELERADIOTHERAPY-X-RAY,RADIUM,COBALT ETC.<br>X304 – THERA. RADIOL.-TELERADIOTHERAPY-MINOR<br>X310 – TREATMENT PLANNING LEVEL 1<br>X311 – TREATMENT PLANNING LEVEL 2<br>X312 – TREATMENT PLANNING LEVEL 3<br>X313 – TREATMENT PLANNING LEVEL 4<br><br>*Brachytherapy*<br>X305 – THER.RADIOL.INTRACAVITORY CONTACT X-RAY THERAPY 1ST APPLIC.<br>X306 – THER.RADIOL.INTRACAVITORY CONTACT X-RAY THERAPY REP.APPLIC.<br>X322 – THERA. RADIOL.-RADIUM-SEALED SOURCE-TREAT. PLANNING<br>X323 – THERAP.RAD.-RADIUM-SEALED SOURCE-INTRACAVITARY-1ST APPL.<br>X324 – THERA. RADIOL.-RADIUM-SEALED SOURCE-INTERSTITIAL APPLICATION<br>X325 – THERA. RADIOL.-RADIUM-SEALED SOURCE-PLAQUE,MOULD<br>X326 – THERA.RADIOL.-RADIOISOTOPES NON SEALED SOURCE-THYROID CA.<br>X329 – THERA.RADIOL.-RADIOISOTOPES-METASTA.CA BONE-NON SEALED SOURC<br>X330 – THERAP.RAD.RADIOISOTOPES-ASCITES/PLEURAL EFFUSIONNOSEALSOURC<br>X336 – THERAPEU.RADIOL.-RADIOISOTOPES-PROSTATE MALIGNANCY

		DAD/SDS/NACRS	Intervention code	*External beam radiotherapy* substring position 4 length 2 = "27"<br><br>*Brachytherapy* substring position 4 length 2 = "26"
		ALR (radiation)		Any treatment records
Chronic conditions*	Osteoarthritis	OHIP	ICD-9	715, 274, 710, 711, 716, 718, 720, 727, 728, 729, 739
		DAD/SDS	ICD-10	M15, M16, M17, M18, M19<br>M00, M01, M02, M03, M07, M10, M11, M12, M13, M14, M20, M21, M22, M23, M24, M25, M30, M31, M32, M33, M34, M35, M36, M65, M66, M67, M68, M69, M7
	Cardiac Arrhythmia	OHIP	ICD-9	427
		DAD/SDS	ICD-10	I480, I481
	Mood disorders	OHIP	ICD-9	296, 300, 309, 311
		DAD/SDS	ICD-10	F30, F31, F32, F33, F341, F348, F349, F38, F39, F40, F41, F42, F431, F432, F438, F44, F450, F451, F452, F48, F530, F680, F930, F99
	Other mental health disorders	OHIP	ICD-9	291, 292, 295, 297, 298, 299, 301, 302, 303, 304, 305, 306, 307, 313, 314, 315, 319
		DAD/SDS	ICD-10	F1, F2, F04, F050, F058, F059, F060, F061, F062, F063, F064, F07, F08, F340, F35, F36, F37, F430, F439, F453, F454, F458, F46, F47, F49, F50, F51, F52, F531, F538, F539, F54, F55, F56, F57, F58, F59, F60, F61, F62, F63, F64, F65, F66, F67, F681, F688, F69, F7, F8, F90, F91, F92, F931, F932, F933, F938, F393, F94, F95, F96, F97, F98
	Osteoporosis	OHIP	ICD-9	733
		DAD/SDS	ICD-10	M81, M82
	Renal disease	OHIP	ICD-9	403, 404, 584, 585, 586, 451
		DAD/SDS	ICD-10	N17, N18, N19, T824, Z492, Z992
	Stroke	OHIP	ICD-9	430, 431, 432, 434, 436
		DAD/SDS	ICD-10	I60, I61, I62, I63, I64

Chronic coronary disease	OHIP	ICD-9	411, 412, 413, 414
	DAD/SDS	ICD-10	I20, I22, I23, I24, I25
Acute myocardial infarction	DAD/SDS	ICD-10	I21

* All chronic conditions were obtained from validated diagnostic codes[12]. Definitions were defined based on either: 1) minimum of 3 OHIP claims, each claim greater than 30 days apart and less than 2 years apart, or 2) minimum of one inpatient hospitalization with any of the diagnostic codes.

Table S4. Definitions of cancer-specific death.

Definition	Data source(s)	Code type	Codes
Colon	ORGD	ICD-9	153 – Malignant neoplasm of colon<br>154 – Malignant neoplasm of rectum rectosigmoid junction and anus<br>159 – Malignant neoplasm of other and ill-defined sites within the digestive organs and peritoneum<br>197 – Secondary malignant neoplasm of respiratory and digestive systems<br>198 – Secondary malignant neoplasm of other specified sites<br>199 – Malignant neoplasm without specification of site
Lung	ORGD	ICD-9	162 – Malignant neoplasm of trachea bronchus and lung<br>197 – Secondary malignant neoplasm of respiratory and digestive systems<br>198 – Secondary malignant neoplasm of other specified sites<br>199 – Malignant neoplasm without specification of site
Breast	ORGD	ICD-9	174 – Malignant neoplasm of female breast<br>197 – Secondary malignant neoplasm of respiratory and digestive systems<br>198 – Secondary malignant neoplasm of other specified sites<br>199 – Malignant neoplasm without specification of site
Ovary	ORGD	ICD-9	183 – Malignant neoplasm of ovary and other uterine adnexa<br>184 – Malignant neoplasm of other and unspecified female genital organs<br>158 – Malignant neoplasm of retroperitoneum and peritoneum<br>159 – Malignant neoplasm of other and ill-defined sites within the digestive organs and peritoneum<br>197 – Secondary malignant neoplasm of respiratory and digestive systems<br>198 – Secondary malignant neoplasm of other specified sites<br>199 – Malignant neoplasm without specification of site
Cervical	ORGD	ICD-9	180 – Malignant neoplasm of cervix uteri<br>179 – Malignant neoplasm of uterus, part unspecified<br>184 – Malignant neoplasm of other and unspecified female genital organs<br>197 – Secondary malignant neoplasm of respiratory and digestive systems<br>198 – Secondary malignant neoplasm of other specified sites<br>199 – Malignant neoplasm without specification of site
Endometrium	ORGD	ICD-9	182 – Malignant neoplasm of body of uterus

			179 – Malignant neoplasm of uterus, part unspecified<br>197 – Secondary malignant neoplasm of respiratory and digestive systems<br>198 – Secondary malignant neoplasm of other specified sites<br>199 – Malignant neoplasm without specification of site
Prostate	ORGD	ICD-9	185 – Malignant neoplasm of prostate<br>197 – Secondary malignant neoplasm of respiratory and digestive systems<br>198 – Secondary malignant neoplasm of other specified sites<br>199 – Malignant neoplasm without specification of site
Testicle	ORGD	ICD-9	186 – Malignant neoplasm of testis<br>197 – Secondary malignant neoplasm of respiratory and digestive systems<br>198 – Secondary malignant neoplasm of other specified sites<br>199 – Malignant neoplasm without specification of site
Thyroid	ORGD	ICD-9	193 – Malignant neoplasm of thyroid gland<br>197 – Secondary malignant neoplasm of respiratory and digestive systems<br>198 – Secondary malignant neoplasm of other specified sites<br>199 – Malignant neoplasm without specification of site
Pancreas	ORGD	ICD-9	157 – Malignant neoplasm of pancreas<br>159 – Malignant neoplasm of other and ill-defined sites within the digestive organs and peritoneum<br>197 – Secondary malignant neoplasm of respiratory and digestive systems<br>198 – Secondary malignant neoplasm of other specified sites<br>199 – Malignant neoplasm without specification of site
Stomach	ORGD	ICD-9	151 – Malignant neoplasm of stomach<br>150 – Malignant neoplasm of esophagus<br>152 – Malignant neoplasm of small intestine including duodenum<br>159 – Malignant neoplasm of other and ill-defined sites within the digestive organs and peritoneum<br>197 – Secondary malignant neoplasm of respiratory and digestive systems<br>198 – Secondary malignant neoplasm of other specified sites<br>199 – Malignant neoplasm without specification of site
Kidney	ORGD	ICD-9	189 – Malignant neoplasm of kidney and other and unspecified urinary organs<br>197 – Secondary malignant neoplasm of respiratory and digestive systems<br>198 – Secondary malignant neoplasm of other specified sites<br>199 – Malignant neoplasm without specification of site
Bladder	ORGD	ICD-9	188 – Malignant neoplasm of bladder<br>189 – Malignant neoplasm of kidney and other and unspecified urinary organs<br>197 – Secondary malignant neoplasm of respiratory and digestive systems<br>198 – Secondary malignant neoplasm of other specified sites<br>199 – Malignant neoplasm without specification of site
Esophagus	ORGD	ICD-9	150 – Malignant neoplasm of esophagus<br>151 – Malignant neoplasm of stomach<br>197 – Secondary malignant neoplasm of respiratory and digestive systems<br>198 – Secondary malignant neoplasm of other specified sites<br>199 – Malignant neoplasm without specification of site

Other gastrointestinal tract	ORGD	ICD-9	150 – Malignant neoplasm of esophagus<br>151 – Malignant neoplasm of stomach<br>152 – Malignant neoplasm of small intestine including duodenum<br>153 – Malignant neoplasm of colon<br>154 – Malignant neoplasm of rectum rectosigmoid junction and anus<br>156 – Malignant neoplasm of gallbladder and extrahepatic bile ducts<br>157 – Malignant neoplasm of pancreas<br>158 – Malignant neoplasm of retroperitoneum and peritoneum<br>159 – Malignant neoplasm of other and ill-defined sites within the digestive organs and peritoneum<br>197 – Secondary malignant neoplasm of respiratory and digestive systems<br>198 – Secondary malignant neoplasm of other specified sites<br>199 – Malignant neoplasm without specification of site
Brain	ORGD	ICD-9	191 – Malignant neoplasm of brain<br>197 – Secondary malignant neoplasm of respiratory and digestive systems<br>237 – Neoplasm of uncertain behavior of endocrine glands and nervous system<br>198 – Secondary malignant neoplasm of other specified sites<br>199 – Malignant neoplasm without specification of site
Melanoma	ORGD	ICD-9	172 – Malignant melanoma of skin<br>173 – Oth and unspec malignant neoplasm of skin<br>197 – Secondary malignant neoplasm of respiratory and digestive systems<br>198 – Secondary malignant neoplasm of other specified sites<br>199 – Malignant neoplasm without specification of site
Head and neck	ORGD	ICD-9	14 – Malignant Neoplasm of Lip, Oral Cavity, and Pharynx<br>176 – Kaposi's sarcoma<br>197 – Secondary malignant neoplasm of respiratory and digestive systems<br>198 – Secondary malignant neoplasm of other specified sites<br>199 – Malignant neoplasm without specification of site
Other solid tumour	ORGD	ICD-9	14 – Malignant Neoplasm of Lip, Oral Cavity, and Pharynx<br>150 – Malignant neoplasm of esophagus<br>151 – Malignant neoplasm of stomach<br>152 – Malignant neoplasm of small intestine including duodenum<br>153 – Malignant neoplasm of colon<br>154 – Malignant neoplasm of rectum rectosigmoid junction and anus<br>156 – Malignant neoplasm of gallbladder and extrahepatic bile ducts<br>157 – Malignant neoplasm of pancreas<br>158 – Malignant neoplasm of retroperitoneum and peritoneum<br>159 – Malignant neoplasm of other and ill-defined sites within the digestive organs and peritoneum<br>16 – Malignant Neoplasm of Respiratory and Intrathoracic Organs<br>17 – Malignant Neoplasm of Bone, Connective Tissue, Skin, and Breast<br>18 – Malignant Neoplasm of Genitourinary Organs<br>19 – Malignant Neoplasm of Other and Unspecified Sites

Table S5. Inclusion table for cancer subjects.

	Frequency	Percent
Total incident cancers diagnosis in accrual period	673,834	
**Exclusion**		
1. Missing topology information	7	0.00
2. Incident liver cancer	9,756	1.45
3. Incident hematological cancer	81,379	12.08
4. No baseline platelet value	449,104	66.65
**Eligible cancer diagnosis for inclusion cohort**	133,588	19.83

Table S6. Exclusion table for study cohort.

	Frequency	Percent
Total incident cancers meeting inclusion criteria	133,588	
Exclusion		
1. Missing date of birth	≤ 5	0
2. Missing sex	≤ 5	0
3. Age <18 on index date	441	0.33
4. Age >100 on index date	73	0.05
5. Death date prior to/on index date	1,918	1.44
6. OHIP ineligible on index date	126	0.09
7. History of cancer in the OCR prior to index date	12,390	9.27
8. History of cancer therapy (chemotherapy or radiotherapy) in the two years prior to index date	5,014	3.75
9. Multiple primary cancers on index date	1,394	1.04
**Eligible cancer subjects**	112,231	84.01

Table S7. Descriptive table of cancer patients (meeting all exclusion criteria) with versus without a baseline platelet count.

Description	Value	Total	Baseline CBC	No baseline CBC	Standardized Difference
Overall		500,386	112,231 (22.4%)	388,155 (77.6%)	
General demographics					
Calendar year (cont.)	Mean (SD)	2011.5 (2.9)	2013.2 (2.4)	2011.1 (2.8)	0.78
	Median (IQR)	2012 (2009–2014)	2014 (2011–2015)	2011 (2009–2013)	
Calendar year (cat.)	2007–2009	147,885 (29.6%)	12,679 (11.3%)	135,206 (34.8%)	0.58
	2010–2012	149,578 (29.9%)	26,849 (23.9%)	122,729 (31.6%)	0.17
	2013–2016	202,923 (40.6%)	72,703 (64.8%)	130,220 (33.5%)	0.66
Sex	Female	252,066 (50.4%)	56,524 (50.4%)	195,542 (50.4%)	0
	Male	248,320 (49.6%)	55,707 (49.6%)	192,613 (49.6%)	
Age (cont.)	Mean (SD)	65.2 (14.1)	66.9 (14.3)	64.7 (14.0)	0.15
	Median (IQR)	66.0 (56.2–75.5)	68.0 (57.8–77.5)	65.5 (55.8–74.8)	
Age (cat.)	18–29	7,071 (1.4%)	1,474 (1.3%)	5,597 (1.4%)	0.01
	30–39	17,438 (3.5%)	3,352 (3.0%)	14,086 (3.6%)	0.04
	40–49	45,381 (9.1%)	8,819 (7.9%)	36,562 (9.4%)	0.06
	50–59	98,746 (19.7%)	19,640 (17.5%)	79,106 (20.4%)	0.07
	60–69	138,191 (27.6%)	29,287 (26.1%)	108,904 (28.1%)	0.04
	70–79	116,358 (23.3%)	27,954 (24.9%)	88,404 (22.8%)	0.05
	80–89	66,601 (13.3%)	18,445 (16.4%)	48,156 (12.4%)	0.11
	90–100	10,600 (2.1%)	3,260 (2.9%)	7,340 (1.9%)	0.07
Neighbourhood income quintile	1 - Low	96,029 (19.2%)	22,374 (19.9%)	73,655 (19.0%)	0.02
	2	100,846 (20.2%)	23,340 (20.8%)	77,506 (20.0%)	0.02
	3	98,596 (19.7%)	22,225 (19.8%)	76,371 (19.7%)	0
	4	100,394 (20.1%)	21,812 (19.4%)	78,582 (20.2%)	0.02
	5 - High	102,822 (20.5%)	22,171 (19.8%)	80,651 (20.8%)	0.03
	Missing	1,699 (0.3%)	309 (0.3%)	1,390 (0.4%)	
Low-income senior (age 66+)	Yes	40,634 (16.2%)	10,204 (16.4%)	30,430 (16.2%)	0.01
Residence location	Urban	432,560 (86.4%)	97,523 (86.9%)	335,037 (86.3%)	0.02
	Rural	67,416 (13.5%)	14,571 (13.0%)	52,845 (13.6%)	0.02
	Missing	410 (0.1%)	137 (0.1%)	273 (0.1%)	
Recently living in Long-Term Care	Yes	8,309 (1.7%)	2,424 (2.2%)	5,885 (1.5%)	0.05
Landed immigrant (cat.)	Non-immigrant	450,600 (90.1%)	99,372 (88.5%)	351,228 (90.5%)	0.06
	Recent immigrant (≤10 years)	14,442 (2.9%)	3,135 (2.8%)	11,307 (2.9%)	0.01
	Past immigrant (>10 years)	35,344 (7.1%)	9,724 (8.7%)	25,620 (6.6%)	0.08
Ethnicity	General Population	475,426 (95.0%)	105,240 (93.8%)	370,186 (95.4%)	0.07

	Chinese	17,044 (3.4%)	4,879 (4.3%)	12,165 (3.1%)	0.06
	South Asian	7,916 (1.6%)	2,112 (1.9%)	5,804 (1.5%)	0.03
Time eligible in OHIP (years)	Mean (SD)	19.8 (5.5)	21.1 (5.8)	19.4 (5.4)	0.31
	Median (IQR)	20.7 (17.9–23.6)	22.9 (19.6–25.0)	20.2 (17.7–23.0)	
Health Services Utilization					
Any OHIP billings in the year prior	Yes	495,345 (99.0%)	111,616 (99.5%)	383,729 (98.9%)	0.06
Any ODB records on/in the year prior (age 66+)	Yes	241,453 (96.5%)	60,465 (97.1%)	180,988 (96.3%)	0.04
Physician Billings (2 years prior)					
Any core primary care visits to GP/FP	Yes	477,551 (95.4%)	107,481 (95.8%)	370,070 (95.3%)	0.02
Core primary care visits to GP/FP (cont.)	Mean (SD)	2.8 (3.3)	2.9 (3.5)	2.8 (3.3)	0.05
	Median (IQR)	1 (1–3)	2 (1–4)	1 (1–3)	
Core primary care visits to GP/FP (cat.)	0	22,835 (4.6%)	4,750 (4.2%)	18,085 (4.7%)	0.02
	1 - 2	283,140 (56.6%)	61,661 (54.9%)	221,479 (57.1%)	0.04
	3 - 4	104,693 (20.9%)	24,009 (21.4%)	80,684 (20.8%)	0.01
	5 - 9	70,557 (14.1%)	16,980 (15.1%)	53,577 (13.8%)	0.04
	10+	19,161 (3.8%)	4,831 (4.3%)	14,330 (3.7%)	0.03
Emergency and inpatient admissions (2 years prior)					
Any unscheduled ED visits	Yes	249,142 (49.8%)	65,094 (58.0%)	184,048 (47.4%)	0.21
Unscheduled ED visits (cont.)	Mean (SD)	1.2 (2.1)	1.5 (2.4)	1.1 (2.1)	0.15
	Median (IQR)	0 (0–2)	1 (0–2)	0 (0–1)	
Any inpatient hospitalization episodes	Yes	70,996 (14.2%)	20,299 (18.1%)	50,697 (13.1%)	0.14
Inpatient hospitalization episodes (cont.)	Mean (SD)	0.2 (0.6)	0.3 (0.7)	0.2 (0.6)	0.12
	Median (IQR)	0 (0–0)	0 (0–0)	0 (0–0)	
Up to date on cancer screening					
Pap smear (females age 18 to 70)	Yes	102,053 (63.2%)	20,003 (61.0%)	82,050 (63.8%)	0.06
Mammogram (females age 50 to 70)	Yes	82,926 (72.8%)	16,220 (67.1%)	66,706 (74.3%)	0.16
FOBT/sigmoidoscopy/colonoscopy (age 50 to 70)	Yes	141,955 (59.9%)	30,057 (61.4%)	111,898 (59.5%)	0.04
Comorbidities and chronic conditions					
Comorbidities					
Charlson score (cont.)	Mean (SD)	0.3 (1.0)	0.4 (1.1)	0.3 (0.9)	0.12
	Median (IQR)	0.0 (0.0–0.0)	0.0 (0.0–0.0)	0.0 (0.0–0.0)	
Charlson comorbidities (cat.)	0	428,584 (85.7%)	92,158 (82.1%)	336,426 (86.7%)	0.13
	1	33,110 (6.6%)	8,578 (7.6%)	24,532 (6.3%)	0.05
	2	17,028 (3.4%)	4,830 (4.3%)	12,198 (3.1%)	0.06
	3+	21,664 (4.3%)	6,665 (5.9%)	14,999 (3.9%)	0.1
Chronic conditions					
Asthma	Yes	43,072 (8.6%)	10,397 (9.3%)	32,675 (8.4%)	0.03

Congestive heart failure	Yes	33,896 (6.8%)	9,509 (8.5%)	24,387 (6.3%)	0.08
Inflammatory bowel disease	Yes	1,588 (0.3%)	435 (0.4%)	1,153 (0.3%)	0.02
Chronic obstructive pulmonary disease	Yes	44,738 (8.9%)	11,578 (10.3%)	33,160 (8.5%)	0.06
HIV	Yes	809 (0.2%)	171 (0.2%)	638 (0.2%)	0
Hypertension	Yes	264,140 (52.8%)	63,988 (57.0%)	200,152 (51.6%)	0.11
Dementia	Yes	16,950 (3.4%)	4,807 (4.3%)	12,143 (3.1%)	0.06
Diabetes	Yes	85,221 (17.0%)	23,222 (20.7%)	61,999 (16.0%)	0.12
Chronic rheumatoid arthritis	Yes	5,410 (1.1%)	1,464 (1.3%)	3,946 (1.0%)	0.03
Osteoarthritis	Yes	86,044 (17.2%)	20,042 (17.9%)	66,002 (17.0%)	0.02
Mood disorder	Yes	62,418 (12.5%)	13,807 (12.3%)	48,611 (12.5%)	0.01
Other mental health disorder	Yes	25,567 (5.1%)	6,629 (5.9%)	18,938 (4.9%)	0.05
Osteoporosis	Yes	7,114 (1.4%)	1,831 (1.6%)	5,283 (1.4%)	0.02
Renal disease	Yes	18,811 (3.8%)	6,303 (5.6%)	12,508 (3.2%)	0.12
Stroke	Yes	9,398 (1.9%)	2,486 (2.2%)	6,912 (1.8%)	0.03
Chronic coronary syndrome	Yes	48,941 (9.8%)	12,131 (10.8%)	36,810 (9.5%)	0.04
Acute myocardial infarction	Yes	11,096 (2.2%)	2,830 (2.5%)	8,266 (2.1%)	0.03
Medication use (age 66+)					
Concurrent medication use					
Number of concurrent medications being used (cont.)	Mean (SD)	4.2 (3.2)	4.6 (3.3)	4.0 (3.1)	0.18
	Median (IQR)	4 (2–6)	4 (2–7)	4 (2–6)	
Number of concurrent medications being used (cat.)	0	29,466 (11.8%)	6,034 (9.7%)	23,432 (12.5%)	0.09
	1 - 4	119,828 (47.9%)	27,434 (44.1%)	92,394 (49.2%)	0.1
	5 - 9	84,338 (33.7%)	23,467 (37.7%)	60,871 (32.4%)	0.11
	10+	16,584 (6.6%)	5,342 (8.6%)	11,242 (6.0%)	0.1
Recent medication use					
Antiplatelet					
Nonsteroidal anti-inflammatory (ASA-based)	Yes	10,696 (4.3%)	2,062 (3.3%)	8,634 (4.6%)	0.07
Nonsteroidal anti-inflammatory (non-ASA-based)	Yes	49,404 (19.7%)	11,481 (18.4%)	37,923 (20.2%)	0.04
Adenosine disphosphonate inhibitor	Yes	16,755 (6.7%)	4,772 (7.7%)	11,983 (6.4%)	0.05
Cardiovascular					
Coronary vasodilator (nitrate)	Yes	21,264 (8.5%)	5,415 (8.7%)	15,849 (8.4%)	0.01
Beta blocker	Yes	69,115 (27.6%)	18,561 (29.8%)	50,554 (26.9%)	0.06
Calcium channel blocker	Yes	73,721 (29.5%)	19,725 (31.7%)	53,996 (28.7%)	0.06
ACE inhibitor	Yes	79,499 (31.8%)	19,838 (31.9%)	59,661 (31.7%)	0
Angiotensin receptor agonist	Yes	53,630 (21.4%)	14,684 (23.6%)	38,946 (20.7%)	0.07
Lipid-lowering					
Statin	Yes	124,361 (49.7%)	33,080 (53.1%)	91,281 (48.6%)	0.09
Psychotropics					

Tricyclic antidepressant	Yes	11,944 (4.8%)	2,975 (4.8%)	8,969 (4.8%)	0
Selective serotonin reuptake inhibitor	Yes	27,277 (10.9%)	7,311 (11.7%)	19,966 (10.6%)	0.04
Incident cancer type (Ontario Cancer Registry)					
	Breast	79,261 (15.8%)	9,857 (8.8%)	69,404 (17.9%)	0.27
	Prostate	77,572 (15.5%)	8,587 (7.7%)	68,985 (17.8%)	0.31
	Lung	66,390 (13.3%)	20,583 (18.3%)	45,807 (11.8%)	0.18
	Colon	63,655 (12.7%)	17,259 (15.4%)	46,396 (12.0%)	0.1
	Other solid tumour	29,286 (5.9%)	8,051 (7.2%)	21,235 (5.5%)	0.07
	Thyroid	24,656 (4.9%)	5,944 (5.3%)	18,712 (4.8%)	0.02
	Bladder	24,631 (4.9%)	6,963 (6.2%)	17,668 (4.6%)	0.07
	Melanoma	23,661 (4.7%)	2,815 (2.5%)	20,846 (5.4%)	0.15
	Endometrium	18,886 (3.8%)	4,500 (4.0%)	14,386 (3.7%)	0.02
	Head and neck	16,485 (3.3%)	3,160 (2.8%)	13,325 (3.4%)	0.04
	Kidney	15,985 (3.2%)	5,272 (4.7%)	10,713 (2.8%)	0.1
	Pancreas	13,151 (2.6%)	5,195 (4.6%)	7,956 (2.0%)	0.14
	Stomach	10,467 (2.1%)	3,365 (3.0%)	7,102 (1.8%)	0.08
	Ovary	9,111 (1.8%)	3,085 (2.7%)	6,026 (1.6%)	0.08
	Brain	7,405 (1.5%)	2,563 (2.3%)	4,842 (1.2%)	0.08
	Other GI	5,921 (1.2%)	1,726 (1.5%)	4,195 (1.1%)	0.04
	Esophagus	5,511 (1.1%)	1,483 (1.3%)	4,028 (1.0%)	0.03
	Cervix	4,933 (1.0%)	833 (0.7%)	4,100 (1.1%)	0.03
	Testis	3,419 (0.7%)	990 (0.9%)	2,429 (0.6%)	0.03
Follow-up period					
Follow-up time	Mean (SD)	4.0 (3.1)	2.6 (2.4)	4.4 (3.2)	0.62
	Median (IQR)	3.3 (1.2–6.5)	1.9 (0.7–3.9)	4.0 (1.5–7.1)	
Deaths in follow-up period					
Any death	Yes	193,504 (38.7%)	51,738 (46.1%)	141,766 (36.5%)	0.2
Any cancer death	Yes	149,730 (29.9%)	41,968 (37.4%)	107,762 (27.8%)	0.21
Cancer-specific death	Yes	141,081 (28.2%)	40,329 (35.9%)	100,752 (26.0%)	0.22

Table S8. Descriptive table of cancer patients by baseline platelet count category.

Description	Value	Total	1 - Low (≤25th pctl)	2 - Medium (>25 to <75th pctl)	3 - High (≥75th pctl)
Overall		112,231	28,307 (25.2%)	55,703 (49.6%)	28,221 (25.1%)
General demographics					
Calendar year (cont.)	Mean (SD)	2013.2 (2.4)	2013.3 (2.4)	2013.2 (2.4)	2013.0 (2.5)
	Median (IQR)	2014 (2011–2015)	2014 (2012–2015)	2014 (2011–2015)	
Calendar year (cat.)	2007–2009	12,679 (11.3%)	2,634 (9.3%)	6,231 (11.2%)	3,814 (13.5%)
	2010–2012	26,849 (23.9%)	6,781 (24.0%)	13,371 (24.0%)	6,697 (23.7%)
	2013–2016	72,703 (64.8%)	18,892 (66.7%)	36,101 (64.8%)	17,710 (62.8%)
Sex	Female	56,524 (50.4%)	14,241 (50.3%)	28,044 (50.3%)	14,239 (50.5%)
	Male	55,707 (49.6%)	14,066 (49.7%)	27,659 (49.7%)	13,982 (49.5%)
Age (cont.)	Mean (SD)	66.9 (14.3)	68.7 (14.3)	66.4 (14.2)	66.0 (14.2)
	Median (IQR)	68.0 (57.8–77.5)	70.2 (60.1–79.4)	67.4 (57.3–76.9)	66.7 (56.8–76.5)
Age (cat.)	18–29	1,474 (1.3%)	369 (1.3%)	767 (1.4%)	338 (1.2%)
	30–39	3,352 (3.0%)	780 (2.8%)	1,685 (3.0%)	887 (3.1%)
	40–49	8,819 (7.9%)	1,761 (6.2%)	4,581 (8.2%)	2,477 (8.8%)
	50–59	19,640 (17.5%)	4,126 (14.6%)	10,097 (18.1%)	5,417 (19.2%)
	60–69	29,287 (26.1%)	6,976 (24.6%)	14,820 (26.6%)	7,491 (26.5%)
	70–79	27,954 (24.9%)	7,626 (26.9%)	13,636 (24.5%)	6,692 (23.7%)
	80–89	18,445 (16.4%)	5,644 (19.9%)	8,650 (15.5%)	4,151 (14.7%)
	90–100	3,260 (2.9%)	1,025 (3.6%)	1,467 (2.6%)	768 (2.7%)
Neighbourhood income quintile	1 - Low	22,374 (19.9%)	5,592 (19.8%)	10,817 (19.4%)	5,965 (21.1%)
	2	23,340 (20.8%)	5,917 (20.9%)	11,544 (20.7%)	5,879 (20.8%)
	3	22,225 (19.8%)	5,540 (19.6%)	11,099 (19.9%)	5,586 (19.8%)
	4	21,812 (19.4%)	5,475 (19.3%)	10,885 (19.5%)	5,452 (19.3%)
	5 - High	22,171 (19.8%)	5,712 (20.2%)	11,207 (20.1%)	5,252 (18.6%)
	Missing	309 (0.3%)	71 (0.3%)	151 (0.3%)	87 (0.3%)
Low-income senior (age 66+)	Yes	10,204 (16.4%)	3,134 (18.0%)	4,714 (15.6%)	2,356 (16.1%)
Residence location	Urban	97,523 (86.9%)	25,005 (88.3%)	48,358 (86.8%)	24,160 (85.6%)
	Rural	14,571 (13.0%)	3,269 (11.5%)	7,276 (13.1%)	4,026 (14.3%)
	Missing	137 (0.1%)	33 (0.1%)	69 (0.1%)	35 (0.1%)
Recently living in Long-Term Care	Yes	2,424 (2.2%)	677 (2.4%)	1,038 (1.9%)	709 (2.5%)
Landed immigrant (cat.)	Non-immigrant	99,372 (88.5%)	24,907 (88.0%)	49,218 (88.4%)	25,247 (89.5%)
	Recent immigrant (≤10 years)	3,135 (2.8%)	833 (2.9%)	1,539 (2.8%)	763 (2.7%)
	Past immigrant (>10 years)	9,724 (8.7%)	2,567 (9.1%)	4,946 (8.9%)	2,211 (7.8%)

Ethnicity	General Population	105,240 (93.8%)	26,342 (93.1%)	52,201 (93.7%)	26,697 (94.6%)
	Chinese	4,879 (4.3%)	1,531 (5.4%)	2,426 (4.4%)	922 (3.3%)
	South Asian	2,112 (1.9%)	434 (1.5%)	1,076 (1.9%)	602 (2.1%)
Time eligible in OHIP (years)	Mean (SD)	21.1 (5.8)	21.2 (5.9)	21.2 (5.7)	21.1 (5.7)
	Median (IQR)	22.9 (19.6–25.0)	23.0 (19.8–25.1)	22.9 (19.6–25.0)	22.8 (19.4–25.0)
Health Services Utilization					
Any OHIP billings in the year prior	Yes	111,616 (99.5%)	28,202 (99.6%)	55,428 (99.5%)	27,986 (99.2%)
Any ODB records on/in the year prior (age 66+)	Yes	60,465 (97.1%)	17,046 (97.7%)	29,263 (97.1%)	14,156 (96.5%)
Physician Billings (2 years prior)					
Any core primary care visits to GP/FP	Yes	107,481 (95.8%)	27,260 (96.3%)	53,514 (96.1%)	26,707 (94.6%)
Core primary care visits to GP/FP (cont.)	Mean (SD)	2.9 (3.5)	3.0 (3.7)	3.0 (3.5)	2.8 (3.3)
	Median (IQR)	2 (1–4)	2 (1–4)	2 (1–4)	1 (1–3)
Core primary care visits to GP/FP (cat.)	0	4,750 (4.2%)	1,047 (3.7%)	2,189 (3.9%)	1,514 (5.4%)
	1 - 2	61,661 (54.9%)	15,279 (54.0%)	30,527 (54.8%)	15,855 (56.2%)
	3 - 4	24,009 (21.4%)	6,172 (21.8%)	12,036 (21.6%)	5,801 (20.6%)
	5 - 9	16,980 (15.1%)	4,494 (15.9%)	8,479 (15.2%)	4,007 (14.2%)
	10+	4,831 (4.3%)	1,315 (4.6%)	2,472 (4.4%)	1,044 (3.7%)
Emergency and inpatient admissions (2 years prior)					
Any unscheduled ED visits	Yes	65,094 (58.0%)	16,583 (58.6%)	31,558 (56.7%)	16,953 (60.1%)
Unscheduled ED visits (cont.)	Mean (SD)	1.5 (2.4)	1.5 (2.7)	1.4 (2.2)	1.5 (2.5)
	Median (IQR)	1 (0–2)	1 (0–2)	1 (0–2)	1 (0–2)
Any inpatient hospitalization episodes	Yes	20,299 (18.1%)	5,578 (19.7%)	9,355 (16.8%)	5,366 (19.0%)
Inpatient hospitalization episodes (cont.)	Mean (SD)	0.3 (0.7)	0.3 (0.8)	0.2 (0.7)	0.3 (0.7)
	Median (IQR)	0 (0–0)	0 (0–0)	0 (0–0)	0 (0–0)
Up to date on cancer screening					
Pap smear (females age 18 to 70)	Yes	20,003 (61.0%)	4,813 (63.2%)	10,520 (63.0%)	4,670 (55.2%)
Mammogram (females age 50 to 70)	Yes	16,220 (67.1%)	3,937 (69.3%)	8,565 (69.4%)	3,718 (60.3%)
FOBT/sigmoidoscopy/colonoscopy (age 50 to 70)	Yes	30,057 (61.4%)	7,348 (66.2%)	15,647 (62.8%)	7,062 (54.7%)
Comorbidities and chronic conditions					
Comorbidities					
Charlson score (cont.)	Mean (SD)	0.4 (1.1)	0.5 (1.2)	0.4 (1.1)	0.4 (1.1)
	Median (IQR)	0 (0–0)	0 (0–0)	0 (0–0)	0 (0–0)
Charlson comorbidities (cat.)	0	92,158 (82.1%)	22,398 (79.1%)	46,492 (83.5%)	23,268 (82.4%)
	1	8,578 (7.6%)	2,351 (8.3%)	4,038 (7.2%)	2,189 (7.8%)
	2	4,830 (4.3%)	1,482 (5.2%)	2,190 (3.9%)	1,158 (4.1%)
	3+	6,665 (5.9%)	2,076 (7.3%)	2,983 (5.4%)	1,606 (5.7%)
Chronic conditions					

Asthma	Yes	10,397 (9.3%)	2,544 (9.0%)	5,272 (9.5%)	2,581 (9.1%)
Congestive heart failure	Yes	9,509 (8.5%)	3,292 (11.6%)	4,329 (7.8%)	1,888 (6.7%)
Inflammatory bowel disease	Yes	435 (0.4%)	96 (0.3%)	172 (0.3%)	167 (0.6%)
Chronic obstructive pulmonary disease	Yes	11,578 (10.3%)	2,994 (10.6%)	5,588 (10.0%)	2,996 (10.6%)
HIV	Yes	171 (0.2%)	53 (0.2%)	79 (0.1%)	39 (0.1%)
Hypertension	Yes	63,988 (57.0%)	17,283 (61.1%)	31,249 (56.1%)	15,456 (54.8%)
Dementia	Yes	4,807 (4.3%)	1,389 (4.9%)	2,200 (3.9%)	1,218 (4.3%)
Diabetes	Yes	23,222 (20.7%)	6,388 (22.6%)	11,128 (20.0%)	5,706 (20.2%)
Chronic rheumatoid arthritis	Yes	1,464 (1.3%)	393 (1.4%)	708 (1.3%)	363 (1.3%)
Osteoarthritis	Yes	20,042 (17.9%)	5,627 (19.9%)	9,878 (17.7%)	4,537 (16.1%)
Mood disorder	Yes	13,807 (12.3%)	3,596 (12.7%)	6,848 (12.3%)	3,363 (11.9%)
Other mental health disorder	Yes	6,629 (5.9%)	1,794 (6.3%)	3,103 (5.6%)	1,732 (6.1%)
Osteoporosis	Yes	1,831 (1.6%)	526 (1.9%)	877 (1.6%)	428 (1.5%)
Renal disease	Yes	6,303 (5.6%)	2,147 (7.6%)	2,772 (5.0%)	1,384 (4.9%)
Stroke	Yes	2,486 (2.2%)	724 (2.6%)	1,147 (2.1%)	615 (2.2%)
Chronic coronary syndrome	Yes	12,131 (10.8%)	4,089 (14.4%)	5,685 (10.2%)	2,357 (8.4%)
Acute myocardial infarction	Yes	2,830 (2.5%)	875 (3.1%)	1,338 (2.4%)	617 (2.2%)
Medication use (age 66+)					
Concurrent medication use					
Number of concurrent medications being used (cont.)	Mean (SD)	4.6 (3.3)	4.8 (3.4)	4.5 (3.3)	4.6 (3.3)
	Median (IQR)	4 (2–7)	4 (2–7)	4 (2–7)	4 (2–7)
Number of concurrent medications being used (cat.)	0	6,034 (9.7%)	1,560 (8.9%)	2,988 (9.9%)	1,486 (10.1%)
	1 - 4	27,434 (44.1%)	7,459 (42.7%)	13,603 (45.1%)	6,372 (43.4%)
	5 - 9	23,467 (37.7%)	6,814 (39.0%)	11,053 (36.7%)	5,600 (38.2%)
	10+	5,342 (8.6%)	1,623 (9.3%)	2,503 (8.3%)	1,216 (8.3%)
Recent medication use					
Antiplatelet					
Nonsteroidal anti-inflammatory (ASA-based)	Yes	2,062 (3.3%)	564 (3.2%)	999 (3.3%)	499 (3.4%)
Nonsteroidal anti-inflammatory (non-ASA-based)	Yes	11,481 (18.4%)	3,149 (18.0%)	5,513 (18.3%)	2,819 (19.2%)
Adenosine disphosphonate inhibitor	Yes	4,772 (7.7%)	1,504 (8.6%)	2,256 (7.5%)	1,012 (6.9%)
Cardiovascular					
Coronary vasodilator (nitrate)	Yes	5,415 (8.7%)	1,845 (10.6%)	2,515 (8.3%)	1,055 (7.2%)
Beta blocker	Yes	18,561 (29.8%)	6,178 (35.4%)	8,620 (28.6%)	3,763 (25.6%)
Calcium channel blocker	Yes	19,725 (31.7%)	5,329 (30.5%)	9,513 (31.6%)	4,883 (33.3%)
ACE inhibitor	Yes	19,838 (31.9%)	5,729 (32.8%)	9,476 (31.4%)	4,633 (31.6%)
Angiotensin receptor agonist	Yes	14,684 (23.6%)	4,363 (25.0%)	7,072 (23.5%)	3,249 (22.1%)
Lipid-lowering					
Statin	Yes	33,080 (53.1%)	10,039 (57.5%)	15,912 (52.8%)	7,129 (48.6%)

Psychotropics					
Tricyclic antidepressant	Yes	2,975 (4.8%)	767 (4.4%)	1,446 (4.8%)	762 (5.2%)
Selective serotonin reuptake inhibitor	Yes	7,311 (11.7%)	1,971 (11.3%)	3,547 (11.8%)	1,793 (12.2%)
Incident cancer type (Ontario Cancer Registry)					
	Lung	20,583 (18.3%)	5,171 (18.3%)	10,256 (18.4%)	5,156 (18.3%)
	Colon	17,259 (15.4%)	4,368 (15.4%)	8,561 (15.4%)	4,330 (15.3%)
	Breast	9,857 (8.8%)	2,483 (8.8%)	4,875 (8.8%)	2,499 (8.9%)
	Prostate	8,587 (7.7%)	2,157 (7.6%)	4,269 (7.7%)	2,161 (7.7%)
	Other solid tumour	8,051 (7.2%)	2,036 (7.2%)	3,995 (7.2%)	2,020 (7.2%)
	Bladder	6,963 (6.2%)	1,768 (6.2%)	3,445 (6.2%)	1,750 (6.2%)
	Thyroid	5,944 (5.3%)	1,497 (5.3%)	2,943 (5.3%)	1,504 (5.3%)
	Kidney	5,272 (4.7%)	1,334 (4.7%)	2,618 (4.7%)	1,320 (4.7%)
	Pancreas	5,195 (4.6%)	1,309 (4.6%)	2,576 (4.6%)	1,310 (4.6%)
	Endometrium	4,500 (4.0%)	1,126 (4.0%)	2,238 (4.0%)	1,136 (4.0%)
	Stomach	3,365 (3.0%)	847 (3.0%)	1,673 (3.0%)	845 (3.0%)
	Head and neck	3,160 (2.8%)	794 (2.8%)	1,575 (2.8%)	791 (2.8%)
	Ovary	3,085 (2.7%)	781 (2.8%)	1,527 (2.7%)	777 (2.8%)
	Melanoma	2,815 (2.5%)	710 (2.5%)	1,397 (2.5%)	708 (2.5%)
	Brain	2,563 (2.3%)	650 (2.3%)	1,267 (2.3%)	646 (2.3%)
	Other GI	1,726 (1.5%)	437 (1.5%)	856 (1.5%)	433 (1.5%)
	Esophagus	1,483 (1.3%)	375 (1.3%)	734 (1.3%)	374 (1.3%)
	Testis	990 (0.9%)	253 (0.9%)	489 (0.9%)	248 (0.9%)
	Cervix	833 (0.7%)	211 (0.7%)	409 (0.7%)	213 (0.8%)
Complete blood count					
Number of baseline CBC observations (cont.)	Mean (SD)	1.5 (1.3)	1.5 (1.5)	1.4 (1.2)	1.5 (1.4)
	Median (IQR)	1 (1–2)	1 (1–1)	1 (1–1)	1 (1–2)
Number of baseline CBC observations (cat.)	1	83,819 (74.7%)	21,324 (75.3%)	42,353 (76.0%)	20,142 (71.4%)
	2	18,559 (16.5%)	4,512 (15.9%)	8,935 (16.0%)	5,112 (18.1%)
	3+	9,853 (8.8%)	2,471 (8.7%)	4,415 (7.9%)	2,967 (10.5%)
Days from baseline CBC to cancer diagnosis	Mean (SD)	12.6 (9.5)	12.4 (9.6)	12.8 (9.5)	12.4 (9.5)
	Median (IQR)	12 (4–21)	12 (4–21)	12 (4–21)	12 (4–21)
Number of CBC observations in the follow-up period (cont.)	Mean (SD)	15.0 (20.3)	15.8 (21.2)	14.8 (19.4)	14.8 (21.0)
	Median (IQR)	8 (3–20)	9 (3–20)	8 (3–19)	8 (2–20)
Number of CBC observations in the follow-up period (cat.)	0	13,109 (11.7%)	2,674 (9.4%)	6,121 (11.0%)	4,314 (15.3%)
	1 - 4	25,385 (22.6%)	6,235 (22.0%)	13,013 (23.4%)	6,137 (21.7%)
	5 - 9	21,958 (19.6%)	5,705 (20.2%)	11,188 (20.1%)	5,065 (17.9%)
	10 - 19	23,405 (20.9%)	6,205 (21.9%)	11,557 (20.7%)	5,643 (20.0%)
	20 - 29	11,679 (10.4%)	3,086 (10.9%)	5,789 (10.4%)	2,804 (9.9%)

	30+	16,695 (14.9%)	4,402 (15.6%)	8,035 (14.4%)	4,258 (15.1%)
Follow-up period					
Follow-up time	Mean (SD)	2.6 (2.4)	2.7 (2.3)	2.7 (2.4)	2.3 (2.5)
	Median (IQR)	1.9 (0.7–3.9)	2.1 (0.9–4.0)	2.0 (0.8–4.1)	1.5 (0.3–3.5)
Deaths in follow-up period					
Any death	Yes	51,738 (46.1%)	11,940 (42.2%)	24,085 (43.2%)	15,713 (55.7%)
Any cancer death	Yes	41,968 (37.4%)	9,048 (32.0%)	19,600 (35.2%)	13,320 (47.2%)
Cancer-specific death	Yes	40,329 (35.9%)	8,666 (30.6%)	18,845 (33.8%)	12,818 (45.4%)

Table S9. Sex- and cancer-specific platelet count reference distributions for exposure definition.

**Sex**	**Cancer Type**	**Frequency**	**Platelet count distribution**						
			Mean	Standard Deviation	P10	P25	P50	P75	P90
Female	All cancers	56,524	291.5	105.2	187	224	272	336	420
	Bladder	1,652	273.0	99.5	176	212	255	309	388
	Brain	1,154	266.2	71.1	180	216	259	309	361
	Breast	9,857	261.3	76.3	181	214	252	297	347
	**Cervix***	**833**	**306.4**	**112.7**	**197**	**235**	**284**	**350**	**437**
	**Colon***	**8,188**	**330.3**	**123.2**	**201**	**246**	**307**	**389**	**489**
	Endometrium	4,500	276.4	87.3	189	221	262	311	381
	**Esophagus***	**358**	**298.3**	**104.8**	**190**	**229**	**276**	**354**	**435**
	Head and neck	788	267.0	78.2	178	218	261	311	366
	Kidney	1,842	284.9	105.6	183	219	263	326	417
	**Lung***	**10,036**	**308.5**	**113.2**	**189**	**232**	**289**	**363**	**451**
	Melanoma	1,224	261.7	75.9	177	214	252.5	297	349
	**Other GI***	**980**	**294.2**	**98.7**	**189**	**226.5**	**279**	**345**	**421**
	**Other solid tumour***	**3,660**	**298.7**	**120.4**	**183**	**224**	**276**	**348**	**449**
	**Ovary***	**3,085**	**340.4**	**122.4**	**210**	**255**	**317**	**403**	**498**
	Pancreas	2,536	270.5	93.8	167	207	257	321	385
	**Stomach***	**1,224**	**312.2**	**125.4**	**191**	**237.5**	**292**	**366**	**454**
	Thyroid	4,607	256.6	63.3	185	213	251	292	336
Male	All cancers	55,707	258.5	99.3	159	194	240	300	380
	Bladder	5,311	229.1	76.1	151	180	218	262	319
	Brain	1,409	235.7	65.4	161	193	231	270	313
	**Colon***	**9,071**	**284.9**	**110.7**	**172**	**210**	**263**	**336**	**425**
	**Esophagus***	**1,125**	**280.8**	**108.2**	**175**	**210**	**264**	**327**	**411**
	Head and neck	2,372	243.8	78.5	161	192	234.5	279.5	340
	Kidney	3,430	250.8	95.5	161	191	231	288	358

**Lung***	**10,547**	**288.5**	**115.3**	**168**	**211**	**268**	**343**	**435**
Melanoma	1,591	227.9	72.6	155	184	217	257	309
**Other GI***	**746**	**267.3**	**111.3**	**154**	**199**	**246.5**	**312**	**392**
**Other solid tumour***	**4,391**	**259.3**	**103.1**	**151**	**190**	**242**	**308**	**387**
Pancreas	2,659	243.9	90.3	148	184	230	286	355
Prostate	8,587	228.8	73.8	152	182	219	263	316
**Stomach***	**2,141**	**283.5**	**107.8**	**171**	**209**	**266**	**335**	**419**
Testis	990	244.5	68.4	172	202	235	278	326
Thyroid	1,337	228.9	63.2	162	187	220	259	305

* Bolded rows have mean values that exceed the overall mean for the respective sex. P10 = 10th percentile, P25 = 25th percentile, P50 = 50th percentile, P75 = 75th percentile, P90 = 90th percentile.

Table S10. Distribution of aggregate cancer stage by cancer site.

Cancer type	Total	Stage I	Stage II	Stage III	Stage IV	Unknown
All cancers	*112,231*	*19,058 (17.0%)*	*16,936 (15.1%)*	*15,098 (13.5%)*	*23,406 (20.9%)*	*37,733 (33.6%)*
Lung	20,583 (18.3%)	3,506 (17.0%)	1,416 (6.9%)	3,627 (17.6%)	10,273 (49.9%)	1,761 (8.6%)
Colon	17,259 (15.4%)	2,939 (17.0%)	4,414 (25.6%)	4,790 (27.8%)	3,771 (21.8%)	1,345 (7.8%)
Breast	9,857 (8.8%)	3,667 (37.2%)	3,488 (35.4%)	1,415 (14.4%)	838 (8.5%)	449 (4.6%)
Prostate	8,587 (7.7%)	1,480 (17.2%)	3,772 (43.9%)	779 (9.1%)	1,738 (20.2%)	818 (9.5%)
Other solid tumour	8,051 (7.2%)	431 (5.4%)	490 (6.1%)	436 (5.4%)	634 (7.9%)	6,060 (75.3%)
Bladder	6,963 (6.2%)	171 (2.5%)	368 (5.3%)	108 (1.6%)	364 (5.2%)	5,952 (85.5%)
Thyroid	5,944 (5.3%)	1,431 (24.1%)	219 (3.7%)	236 (4.0%)	167 (2.8%)	3,891 (65.5%)
Kidney	5,272 (4.7%)	524 (9.9%)	103 (2.0%)	299 (5.7%)	596 (11.3%)	3,750 (71.1%)
Pancreas	5,195 (4.6%)	198 (3.8%)	598 (11.5%)	369 (7.1%)	1,320 (25.4%)	2,710 (52.2%)
Endometrium	4,500 (4.0%)	2,050 (45.6%)	270 (6.0%)	394 (8.8%)	273 (6.1%)	1,513 (33.6%)
Stomach	3,365 (3.0%)	172 (5.1%)	218 (6.5%)	271 (8.1%)	698 (20.7%)	2,006 (59.6%)
Head and neck	3,160 (2.8%)	363 (11.5%)	344 (10.9%)	427 (13.5%)	1,305 (41.3%)	721 (22.8%)
Ovary	3,085 (2.7%)	461 (14.9%)	190 (6.2%)	1,014 (32.9%)	411 (13.3%)	1,009 (32.7%)
Melanoma	2,815 (2.5%)	714 (25.4%)	460 (16.3%)	224 (8.0%)	152 (5.4%)	1,265 (44.9%)
Brain	2,563 (2.3%)	0 (0.0%)	0 (0.0%)	0 (0.0%)	0 (0.0%)	2,563 (100.0%)
Other GI	1,726 (1.5%)	74 (4.3%)	185 (10.7%)	246 (14.3%)	303 (17.6%)	918 (53.2%)
Esophagus	1,483 (1.3%)	49 (3.3%)	142 (9.6%)	218 (14.7%)	409 (27.6%)	665 (44.8%)
Testis	990 (0.9%)	512 (51.7%)	116 (11.7%)	81 (8.2%)	0 (0.0%)	281 (28.4%)
Cervix	833 (0.7%)	316 (37.9%)	143 (17.2%)	164 (19.7%)	154 (18.5%)	56 (6.7%)

Table S11. Cancer-specific mortality rates and hazard ratios by baseline platelet count category (exposure definition 1), for all cancers.

					Unadjusted		Patient variable adjustment*		Patient variable and cancer stage adjustment**	
**Cancer**	**Platelet category**	**Person-years**	**Cancer-specific deaths**	**Rate (% per year)**	**HR (95% CI)**	**P-value**	**HR (95% CI)**	**P-value**	**HR (95% CI)**	**P-value**
All cancers	1 - Low (≤25th pctl)	76,425.8	8,666	11.3	0.91 (0.88–0.93)	<0.0001	0.84 (0.82–0.86)	<0.0001	0.91 (0.88–0.94)	<0.0001
	2 - Medium (>25 to <75th pctl)	153,022.0	18,845	12.3	1.00 (Reference)		1.00 (Reference)		1.00 (Reference)	
	3 - High (≥75th pctl)	65,811.5	12,818	19.5	1.52 (1.48–1.55)	<0.0001	1.55 (1.52–1.59)	<0.0001	1.23 (1.20–1.26)	<0.0001
Colon	1 - Low (≤25th pctl)	13,656.9	926	6.8	0.70 (0.65–0.76)	<0.0001	0.69 (0.64–0.75)	<0.0001	0.92 (0.84–0.99)	0.0334
	2 - Medium (>25 to <75th pctl)	26,021.7	2,510	9.6	1.00 (Reference)		1.00 (Reference)		1.00 (Reference)	
	3 - High (≥75th pctl)	10,913.0	1,884	17.3	1.70 (1.60–1.80)	<0.0001	1.71 (1.61–1.81)	<0.0001	1.32 (1.24–1.40)	<0.0001
Lung	1 - Low (≤25th pctl)	8,415.3	2,798	33.2	0.80 (0.77–0.84)	<0.0001	0.75 (0.72–0.79)	<0.0001	0.93 (0.89–0.97)	0.0024
	2 - Medium (>25 to <75th pctl)	14,959.1	6,504	43.5	1.00 (Reference)		1.00 (Reference)		1.00 (Reference)	
	3 - High (≥75th pctl)	4,977.9	3,872	77.8	1.55 (1.48–1.61)	<0.0001	1.57 (1.51–1.63)	<0.0001	1.35 (1.29–1.40)	<0.0001
Breast	1 - Low (≤25th pctl)	9,436.4	298	3.2	1.19 (1.03–1.37)	0.0193	1.05 (0.91–1.21)	0.5042	1.11 (0.96–1.30)	0.1654
	2 - Medium (>25 to <75th pctl)	19,744.7	516	2.6	1.00 (Reference)		1.00 (Reference)		1.00 (Reference)	
	3 - High (≥75th pctl)	9,377.9	428	4.6	1.73 (1.52–1.97)	<0.0001	1.80 (1.58–2.04)	<0.0001	1.23 (1.07–1.41)	0.0028
Prostate	1 - Low (≤25th pctl)	7,499.3	365	4.9	1.66 (1.45–1.90)	<0.0001	1.39 (1.21–1.60)	<0.0001	1.30 (1.11–1.51)	0.0009
	2 - Medium (>25 to <75th pctl)	16,960.5	474	2.8	1.00 (Reference)		1.00 (Reference)		1.00 (Reference)	
	3 - High (≥75th pctl)	8,034.9	434	5.4	1.91 (1.68–2.17)	<0.0001	1.87 (1.64–2.13)	<0.0001	1.39 (1.21–1.61)	<0.0001
Ovary	1 - Low (≤25th pctl)	2,161.7	226	10.5	0.65 (0.56–0.76)	<0.0001	0.63 (0.54–0.74)	<0.0001	0.78 (0.64–0.95)	0.0143
	2 - Medium (>25 to <75th pctl)	4,017.9	650	16.2	1.00 (Reference)		1.00 (Reference)		1.00 (Reference)	
	3 - High (≥75th pctl)	1,699.2	459	27.0	1.60 (1.42–1.80)	<0.0001	1.60 (1.42–1.81)	<0.0001	1.27 (1.10–1.48)	0.0016
Stomach	1 - Low (≤25th pctl)	1,441.4	478	33.2	0.88 (0.79–0.99)	0.0265	0.87 (0.78–0.97)	0.0111	1.08 (0.91–1.28)	0.3949
	2 - Medium (>25 to <75th pctl)	2,745.4	1,035	37.7	1.00 (Reference)		1.00 (Reference)		1.00 (Reference)	
	3 - High (≥75th pctl)	1,191.1	574	48.2	1.25 (1.13–1.38)	<0.0001	1.23 (1.11–1.36)	0.0001	1.03 (0.88–1.21)	0.6992
Pancreas	1 - Low (≤25th pctl)	1,133.3	1,060	93.5	1.12 (1.04–1.20)	0.0033	1.08 (1.00–1.16)	0.0542	0.94 (0.84–1.05)	0.3046
	2 - Medium (>25 to <75th pctl)	2,581.5	2,028	78.6	1.00 (Reference)		1.00 (Reference)		1.00 (Reference)	

	3 - High (≥75th pctl)	1,084.5	1,104	101.8	1.26 (1.17–1.35)	<0.0001	1.27 (1.18–1.37)	<0.0001	1.23 (1.10–1.37)	0.0002
Kidney	1 - Low (≤25th pctl)	4,253.6	157	3.7	0.89 (0.74–1.08)	0.2417	0.76 (0.63–0.92)	0.005	0.83 (0.63–1.10)	0.1919
	2 - Medium (>25 to <75th pctl)	8,437.8	346	4.1	1.00 (Reference)		1.00 (Reference)		1.00 (Reference)	
	3 - High (≥75th pctl)	3,212.7	533	16.6	3.79 (3.31–4.34)	<0.0001	3.72 (3.25–4.27)	<0.0001	1.64 (1.36–1.98)	<0.0001
Bladder	1 - Low (≤25th pctl)	5,285.2	230	4.4	1.03 (0.88–1.20)	0.7431	0.88 (0.75–1.03)	0.122	0.72 (0.55–0.94)	0.0159
	2 - Medium (>25 to <75th pctl)	10,633.9	446	4.2	1.00 (Reference)		1.00 (Reference)		1.00 (Reference)	
	3 - High (≥75th pctl)	4,677.0	477	10.2	2.41 (2.12–2.75)	<0.0001	2.46 (2.16–2.80)	<0.0001	1.29 (1.05–1.59)	0.0166
Esophagus	1 - Low (≤25th pctl)	478.8	264	55.1	1.02 (0.88–1.19)	0.7678	0.99 (0.85–1.15)	0.8641	0.99 (0.80–1.22)	0.9321
	2 - Medium (>25 to <75th pctl)	962.8	516	53.6	1.00 (Reference)		1.00 (Reference)		1.00 (Reference)	
	3 - High (≥75th pctl)	363.9	292	80.3	1.45 (1.26–1.68)	<0.0001	1.49 (1.29–1.72)	<0.0001	1.09 (0.89–1.32)	0.4196
Other GI	1 - Low (≤25th pctl)	985.7	132	13.4	0.94 (0.76–1.15)	0.5527	0.89 (0.72–1.09)	0.2591	0.75 (0.53–1.06)	0.108
	2 - Medium (>25 to <75th pctl)	2,127.7	291	13.7	1.00 (Reference)		1.00 (Reference)		1.00 (Reference)	
	3 - High (≥75th pctl)	837.9	217	25.9	1.77 (1.48–2.11)	<0.0001	1.84 (1.54–2.19)	<0.0001	1.79 (1.35–2.37)	<0.0001
Thyroid	1 - Low (≤25th pctl)	5,984.2	24	0.4	1.25 (0.75–2.09)	0.3856	1.06 (0.63–1.77)	0.8212	0.50 (0.21–1.18)	0.1135
	2 - Medium (>25 to <75th pctl)	12,113.1	38	0.3	1.00 (Reference)		1.00 (Reference)		1.00 (Reference)	
	3 - High (≥75th pctl)	6,172.7	48	0.8	2.49 (1.63–3.82)	<0.0001	3.31 (2.15–5.10)	<0.0001	1.27 (0.74–2.19)	0.3913
Cervix	1 - Low (≤25th pctl)	748.2	32	4.3	0.58 (0.39–0.86)	0.0065	0.50 (0.33–0.75)	0.0007	0.73 (0.47–1.14)	0.168
	2 - Medium (>25 to <75th pctl)	1,346.7	100	7.4	1.00 (Reference)		1.00 (Reference)		1.00 (Reference)	
	3 - High (≥75th pctl)	535.7	110	20.5	2.61 (1.99–3.42)	<0.0001	2.63 (2.00–3.46)	<0.0001	1.88 (1.40–2.53)	<0.0001
Testis	1 - Low (≤25th pctl)	**SS**	**SS**	0.3	0.97 (0.24–3.86)	0.9607	0.69 (0.17–2.88)	0.6135	1.47 (0.13–16.20)	0.7513
	2 - Medium (>25 to <75th pctl)	**SS**	**SS**	0.3	1.00 (Reference)		1.00 (Reference)		1.00 (Reference)	
	3 - High (≥75th pctl)	**SS**	**SS**	1.5	4.76 (1.83–12.39)	0.0014	4.92 (1.83–13.25)	0.0016	1.52 (0.46–5.04)	0.4895
Endometrium	1 - Low (≤25th pctl)	3,988.2	135	3.4	1.13 (0.91–1.40)	0.2571	0.94 (0.76–1.16)	0.5428	1.18 (0.92–1.52)	0.2011
	2 - Medium (>25 to <75th pctl)	7,996.6	239	3.0	1.00 (Reference)		1.00 (Reference)		1.00 (Reference)	
	3 - High (≥75th pctl)	3,496.7	259	7.4	2.42 (2.03–2.89)	<0.0001	2.69 (2.25–3.21)	<0.0001	1.91 (1.53–2.38)	<0.0001
Brain	1 - Low (≤25th pctl)	868.5	468	53.9	1.11 (0.99–1.24)	0.074	0.91 (0.81–1.02)	0.1236	*NA*	
	2 - Medium (>25 to <75th pctl)	1,910.8	887	46.4	1.00 (Reference)		1.00 (Reference)		*NA*	
	3 - High (≥75th pctl)	1,038.2	461	44.4	1.01 (0.90–1.13)	0.9181	1.05 (0.94–1.18)	0.3669	*NA*	
Melanoma	1 - Low (≤25th pctl)	2,672.7	86	3.2	0.99 (0.76–1.28)	0.9398	0.90 (0.69–1.17)	0.434	0.79 (0.56–1.12)	0.1815

	2 - Medium (>25 to <75th pctl)	5,219.7	170	3.3	1.00 (Reference)		1.00 (Reference)		1.00 (Reference)	
	3 - High (≥75th pctl)	2,421.2	168	6.9	2.16 (1.74–2.67)	<0.0001	2.25 (1.82–2.79)	<0.0001	1.30 (0.99–1.72)	0.0627
Head and neck	1 - Low (≤25th pctl)	2,394.6	129	5.4	0.91 (0.74–1.12)	0.3731	0.79 (0.64–0.98)	0.0303	0.91 (0.72–1.16)	0.4479
	2 - Medium (>25 to <75th pctl)	5,158.0	291	5.6	1.00 (Reference)		1.00 (Reference)		1.00 (Reference)	
	3 - High (≥75th pctl)	2,154.6	222	10.3	1.73 (1.46–2.06)	<0.0001	1.79 (1.50–2.13)	<0.0001	1.52 (1.24–1.86)	<0.0001
Other solid tumour	1 - Low (≤25th pctl)	4,023.6	855	21.2	0.96 (0.88–1.04)	0.2931	0.86 (0.80–0.94)	0.0005	0.92 (0.77–1.09)	0.331
	2 - Medium (>25 to <75th pctl)	8,195.0	1,798	21.9	1.00 (Reference)		1.00 (Reference)		1.00 (Reference)	
	3 - High (≥75th pctl)	2,661.3	1,262	47.4	1.79 (1.67–1.92)	<0.0001	1.78 (1.66–1.92)	<0.0001	1.31 (1.12–1.53)	0.0008

HR = Hazard ratio; pctl = percentile; NA = Not applicable; *SS* = data suppressed due to small number of observations/events. * Adjusted for age at diagnosis (cat.), year of diagnosis (cont.), and common chronic conditions (asthma, congestive heart failure, chronic obstructive pulmonary disease, hypertension, and diabetes). ** Adjusted for age at diagnosis (cat.), year of diagnosis (cont.), common chronic conditions (asthma, congestive heart failure, chronic obstructive pulmonary disease, hypertension, and diabetes), and cancer stage. Only subjects with known cancer stage were included.

Table S12. Cancer-specific mortality rates and hazard ratios by baseline platelet count category (exposure definition 2), for all cancers.

					**Unadjusted**		**Patient variable adjustment***		**Patient variable and cancer stage adjustment****	
**Cancer**	**Platelet category**	**Person-years**	**Cancer-specific deaths**	**Rate (% per year)**	**HR (95% CI)**	**P-value**	**HR (95% CI)**	**P-value**	**HR (95% CI)**	**P-value**
All	1 - Very low (≤10th pctl)	29,043.6	3,624	12.5	0.97 (0.94–1.01)	0.1193	0.86 (0.83–0.90)	<0.0001	0.94 (0.90–0.98)	0.0064
	2 - Low (>10 to ≤25th pctl)	47,382.2	5,042	10.6	0.86 (0.84–0.89)	<0.0001	0.82 (0.79–0.84)	<0.0001	0.90 (0.86–0.93)	<0.0001
	3 - Medium (>25 to <75th pctl)	153,022.0	18,845	12.3	1.00 (Reference)		1.00 (Reference)		1.00 (Reference)	
	4 - High (≥75 to <90th pctl)	41,977.2	7,129	17.0	1.35 (1.31–1.39)	<0.0001	1.38 (1.34–1.42)	<0.0001	1.19 (1.16–1.23)	<0.0001
	5 - Very high (≥90th pctl)	23,834.3	5,689	23.9	1.79 (1.74–1.85)	<0.0001	1.84 (1.79–1.90)	<0.0001	1.28 (1.23–1.33)	<0.0001
Colon	1 - Very low (≤10th pctl)	5,313.0	362	6.8	0.70 (0.63–0.78)	<0.0001	0.67 (0.60–0.75)	<0.0001	0.92 (0.81–1.04)	0.1619
	2 - Low (>10 to ≤25th pctl)	8,343.8	564	6.8	0.70 (0.64–0.77)	<0.0001	0.71 (0.64–0.77)	<0.0001	0.91 (0.83–1.01)	0.0672
	3 - Medium (>25 to <75th pctl)	26,021.7	2,510	9.6	1.00 (Reference)		1.00 (Reference)		1.00 (Reference)	
	4 - High (≥75 to <90th pctl)	6,899.5	1,046	15.2	1.51 (1.41–1.63)	<0.0001	1.51 (1.40–1.62)	<0.0001	1.24 (1.15–1.34)	<0.0001
	5 - Very high (≥90th pctl)	4,013.5	838	20.9	2.01 (1.86–2.17)	<0.0001	2.06 (1.90–2.22)	<0.0001	1.44 (1.32–1.56)	<0.0001
Lung	1 - Very low (≤10th pctl)	3,126.0	1,149	36.8	0.87 (0.82–0.92)	<0.0001	0.79 (0.74–0.84)	<0.0001	1.02 (0.95–1.09)	0.6681
	2 - Low (>10 to ≤25th pctl)	5,289.3	1,649	31.2	0.76 (0.72–0.80)	<0.0001	0.73 (0.69–0.77)	<0.0001	0.88 (0.83–0.93)	<0.0001
	3 - Medium (>25 to <75th pctl)	14,959.1	6,504	43.5	1.00 (Reference)		1.00 (Reference)		1.00 (Reference)	

	4 - High (≥75 to <90th pctl)	3,220.3	2,282	70.9	1.44 (1.37–1.51)	<0.0001	1.45 (1.39–1.53)	<0.0001	1.29 (1.23–1.35)	<0.0001
	5 - Very high (≥90th pctl)	1,757.6	1,590	90.5	1.73 (1.64–1.83)	<0.0001	1.78 (1.68–1.88)	<0.0001	1.44 (1.36–1.53)	<0.0001
Breast	1 - Very low (≤10th pctl)	3,556.0	154	4.3	1.60 (1.34–1.91)	<0.0001	1.39 (1.16–1.67)	0.0003	1.27 (1.04–1.54)	0.018
	2 - Low (>10 to ≤25th pctl)	5,880.4	144	2.4	0.93 (0.77–1.12)	0.4298	0.83 (0.69–1.00)	0.0545	0.99 (0.81–1.21)	0.9388
	3 - Medium (>25 to <75th pctl)	19,744.7	516	2.6	1.00 (Reference)		1.00 (Reference)		1.00 (Reference)	
	4 - High (≥75 to <90th pctl)	5,853.6	207	3.5	1.35 (1.14–1.58)	0.0003	1.41 (1.20–1.66)	<0.0001	1.17 (0.98–1.38)	0.0788
	5 - Very high (≥90th pctl)	3,524.2	221	6.3	2.36 (2.01–2.76)	<0.0001	2.42 (2.06–2.83)	<0.0001	1.30 (1.10–1.54)	0.0021
Prostate	1 - Very low (≤10th pctl)	2,792.8	189	6.8	2.24 (1.89–2.65)	<0.0001	1.76 (1.49–2.09)	<0.0001	1.44 (1.18–1.75)	0.0003
	2 - Low (>10 to ≤25th pctl)	4,706.4	176	3.7	1.30 (1.09–1.54)	0.003	1.14 (0.96–1.36)	0.1392	1.19 (0.98–1.44)	0.073
	3 - Medium (>25 to <75th pctl)	16,960.5	474	2.8	1.00 (Reference)		1.00 (Reference)		1.00 (Reference)	
	4 - High (≥75 to <90th pctl)	5,113.0	213	4.2	1.50 (1.28–1.76)	<0.0001	1.50 (1.28–1.77)	<0.0001	1.25 (1.05–1.49)	0.0141
	5 - Very high (≥90th pctl)	2,921.9	221	7.6	2.59 (2.21–3.04)	<0.0001	2.43 (2.07–2.85)	<0.0001	1.58 (1.32–1.89)	<0.0001
Ovary	1 - Very low (≤10th pctl)	826.6	90	10.9	0.66 (0.53–0.82)	0.0002	0.58 (0.46–0.72)	<0.0001	0.78 (0.57–1.06)	0.1063
	2 - Low (>10 to ≤25th pctl)	1,335.1	136	10.2	0.65 (0.54–0.78)	<0.0001	0.68 (0.56–0.82)	<0.0001	0.78 (0.61–0.99)	0.0407
	3 - Medium (>25 to <75th pctl)	4,017.9	650	16.2	1.00 (Reference)		1.00 (Reference)		1.00 (Reference)	
	4 - High (≥75 to <90th pctl)	1,035.6	271	26.2	1.55 (1.34–1.79)	<0.0001	1.55 (1.34–1.79)	<0.0001	1.29 (1.08–1.53)	0.0053
	5 - Very high (≥90th pctl)	663.6	188	28.3	1.67 (1.42–1.97)	<0.0001	1.69 (1.44–2.00)	<0.0001	1.25 (1.03–1.53)	0.0268
Stomach	1 - Very low (≤10th pctl)	554.7	188	33.9	0.89 (0.76–1.04)	0.1543	0.87 (0.74–1.01)	0.0736	1.02 (0.79–1.33)	0.8614
	2 - Low (>10 to ≤25th pctl)	886.7	290	32.7	0.88 (0.77–1.00)	0.0522	0.87 (0.76–0.99)	0.0353	1.11 (0.91–1.37)	0.3003
	3 - Medium (>25 to <75th pctl)	2,745.4	1,035	37.7	1.00 (Reference)		1.00 (Reference)		1.00 (Reference)	
	4 - High (≥75 to <90th pctl)	734.9	338	46.0	1.19 (1.05–1.34)	0.0061	1.16 (1.02–1.31)	0.0199	1.11 (0.92–1.35)	0.2836
	5 - Very high (≥90th pctl)	456.2	236	51.7	1.35 (1.17–1.55)	<0.0001	1.34 (1.16–1.54)	<0.0001	0.93 (0.75–1.17)	0.5454
Pancreas	1 - Very low (≤10th pctl)	445.9	424	95.1	1.15 (1.04–1.28)	0.009	1.07 (0.97–1.19)	0.1868	0.89 (0.76–1.05)	0.1731
	2 - Low (>10 to ≤25th pctl)	687.3	636	92.5	1.10 (1.00–1.20)	0.0398	1.08 (0.99–1.18)	0.1019	0.97 (0.85–1.11)	0.7063
	3 - Medium (>25 to <75th pctl)	2,581.5	2,028	78.6	1.00 (Reference)		1.00 (Reference)		1.00 (Reference)	
	4 - High (≥75 to <90th pctl)	710.0	642	90.4	1.16 (1.06–1.26)	0.0013	1.16 (1.06–1.26)	0.0013	1.15 (1.01–1.31)	0.0408
	5 - Very high (≥90th pctl)	374.5	462	123.4	1.43 (1.29–1.58)	<0.0001	1.47 (1.33–1.63)	<0.0001	1.40 (1.19–1.64)	<0.0001
Kidney	1 - Very low (≤10th pctl)	1,685.5	72	4.3	1.03 (0.80–1.32)	0.834	0.84 (0.65–1.08)	0.1777	1.03 (0.69–1.55)	0.8689
	2 - Low (>10 to ≤25th pctl)	2,568.0	85	3.3	0.80 (0.63–1.02)	0.0725	0.71 (0.56–0.90)	0.0046	0.74 (0.53–1.03)	0.0753
	3 - Medium (>25 to <75th pctl)	8,437.8	346	4.1	1.00 (Reference)		1.00 (Reference)		1.00 (Reference)	
	4 - High (≥75 to <90th pctl)	2,202.3	233	10.6	2.51 (2.13–2.97)	<0.0001	2.51 (2.13–2.97)	<0.0001	1.39 (1.11–1.76)	0.0046
	5 - Very high (≥90th pctl)	1,010.5	300	29.7	6.30 (5.40–7.36)	<0.0001	6.03 (5.16–7.05)	<0.0001	1.89 (1.53–2.35)	<0.0001

Bladder	1 - Very low (≤10th pctl)	2,049.9	98	4.8	1.11 (0.89–1.38)	0.3672	0.90 (0.72–1.12)	0.3265	0.64 (0.42–0.98)	0.0409
	2 - Low (>10 to ≤25th pctl)	3,235.3	132	4.1	0.98 (0.80–1.18)	0.8016	0.87 (0.72–1.06)	0.1676	0.76 (0.55–1.04)	0.0831
	3 - Medium (>25 to <75th pctl)	10,633.9	446	4.2	1.00 (Reference)		1.00 (Reference)		1.00 (Reference)	
	4 - High (≥75 to <90th pctl)	3,046.2	231	7.6	1.83 (1.56–2.14)	<0.0001	1.90 (1.62–2.23)	<0.0001	1.13 (0.87–1.46)	0.3501
	5 - Very high (≥90th pctl)	1,630.8	246	15.1	3.46 (2.96–4.04)	<0.0001	3.41 (2.91–3.99)	<0.0001	1.47 (1.15–1.88)	0.0022
Esophagus	1 - Very low (≤10th pctl)	173.3	102	58.9	1.03 (0.83–1.28)	0.7762	0.98 (0.79–1.21)	0.8322	0.96 (0.70–1.32)	0.783
	2 - Low (>10 to ≤25th pctl)	305.5	162	53.0	1.02 (0.85–1.21)	0.85	0.99 (0.83–1.19)	0.9338	1.01 (0.79–1.29)	0.9368
	3 - Medium (>25 to <75th pctl)	962.8	516	53.6	1.00 (Reference)		1.00 (Reference)		1.00 (Reference)	
	4 - High (≥75 to <90th pctl)	220.3	167	75.8	1.35 (1.13–1.60)	0.0009	1.36 (1.14–1.62)	0.0007	1.09 (0.86–1.37)	0.4916
	5 - Very high (≥90th pctl)	143.5	125	87.1	1.63 (1.34–1.98)	<0.0001	1.71 (1.40–2.08)	<0.0001	1.09 (0.81–1.45)	0.5746
Other GI	1 - Very low (≤10th pctl)	358.5	58	16.2	1.10 (0.83–1.45)	0.5182	1.05 (0.79–1.39)	0.7385	0.96 (0.60–1.53)	0.8502
	2 - Low (>10 to ≤25th pctl)	627.2	74	11.8	0.84 (0.65–1.09)	0.1944	0.79 (0.61–1.02)	0.0745	0.63 (0.40–0.98)	0.0425
	3 - Medium (>25 to <75th pctl)	2,127.7	291	13.7	1.00 (Reference)		1.00 (Reference)		1.00 (Reference)	
	4 - High (≥75 to <90th pctl)	543.7	114	21.0	1.46 (1.18–1.82)	0.0006	1.53 (1.23–1.90)	0.0001	1.58 (1.13–2.22)	0.0077
	5 - Very high (≥90th pctl)	294.2	103	35.0	2.31 (1.84–2.89)	<0.0001	2.37 (1.89–2.97)	<0.0001	2.15 (1.49–3.12)	<0.0001
Thyroid	1 - Very low (≤10th pctl)	2,307.3	13	0.6	1.71 (0.91–3.21)	0.0959	1.27 (0.68–2.40)	0.455	0.39 (0.14–1.09)	0.0733
	2 - Low (>10 to ≤25th pctl)	3,676.9	11	0.3	0.95 (0.49–1.87)	0.8914	0.88 (0.45–1.72)	0.7062	0.74 (0.24–2.34)	0.6129
	3 - Medium (>25 to <75th pctl)	12,113.1	38	0.3	1.00 (Reference)		1.00 (Reference)		1.00 (Reference)	
	4 - High (≥75 to <90th pctl)	3,818.5	15	0.4	1.28 (0.71–2.33)	0.4138	1.80 (0.99–3.29)	0.056	1.09 (0.50–2.36)	0.8242
	5 - Very high (≥90th pctl)	2,354.2	33	1.4	4.37 (2.74–6.97)	<0.0001	5.37 (3.33–8.68)	<0.0001	1.38 (0.75–2.54)	0.2969
Cervix	1 - Very low (≤10th pctl)	299.6	14	4.7	0.63 (0.36–1.10)	0.1028	0.49 (0.28–0.87)	0.0155	0.68 (0.37–1.26)	0.219
	2 - Low (>10 to ≤25th pctl)	448.6	18	4.0	0.54 (0.33–0.89)	0.0163	0.50 (0.30–0.83)	0.0069	0.78 (0.45–1.36)	0.3781
	3 - Medium (>25 to <75th pctl)	1,346.7	100	7.4	1.00 (Reference)		1.00 (Reference)		1.00 (Reference)	
	4 - High (≥75 to <90th pctl)	358.0	64	17.9	2.37 (1.73–3.24)	<0.0001	2.28 (1.66–3.13)	<0.0001	1.81 (1.29–2.55)	0.0006
	5 - Very high (≥90th pctl)	177.7	46	25.9	3.04 (2.14–4.32)	<0.0001	3.38 (2.36–4.85)	<0.0001	1.99 (1.35–2.94)	0.0006
Testis	1 - Very low (≤10th pctl)	*SS*	*SS*	0.6	1.66 (0.33–8.21)	0.5361	0.95 (0.17–5.41)	0.9547	2.40 (0.23–25.37)	0.4675
	2 - Low (>10 to ≤25th pctl)	*SS*	*SS*	0.2	0.53 (0.06–4.37)	0.5515	0.39 (0.05–3.35)	0.3933	*NA*	
	3 - Medium (>25 to <75th pctl)	1,889.0	6	0.3	1.00 (Reference)		1.00 (Reference)		1.00 (Reference)	
	4 - High (≥75 to <90th pctl)	*SS*	*SS*	0.6	2.19 (0.62–7.76)	0.2248	1.93 (0.51–7.23)	0.3315	0.55 (0.09–3.18)	0.5006
	5 - Very high (≥90th pctl)	327.4	10	3.1	8.97 (3.26–24.69)	<0.0001	11.23 (3.84–32.88)	<0.0001	2.74 (0.79–9.43)	0.1105
Endometrium	1 - Very low (≤10th pctl)	1,595.4	62	3.9	1.27 (0.96–1.68)	0.0964	0.96 (0.72–1.27)	0.7744	1.31 (0.94–1.84)	0.1138
	2 - Low (>10 to ≤25th pctl)	2,392.7	73	3.1	1.03 (0.80–1.34)	0.8005	0.91 (0.70–1.19)	0.4891	1.09 (0.80–1.49)	0.5826

	3 - Medium (>25 to <75th pctl)	7,996.6	239	3.0	1.00 (Reference)		1.00 (Reference)		1.00 (Reference)	
	4 - High (≥75 to <90th pctl)	2,272.1	117	5.1	1.71 (1.37–2.13)	<0.0001	1.80 (1.44–2.25)	<0.0001	1.79 (1.37–2.34)	<0.0001
	5 - Very high (≥90th pctl)	1,224.6	142	11.6	3.71 (3.02–4.57)	<0.0001	4.62 (3.74–5.71)	<0.0001	2.04 (1.56–2.68)	<0.0001
Brain	1 - Very low (≤10th pctl)	331.1	188	56.8	1.15 (0.98–1.35)	0.0803	0.83 (0.70–0.97)	0.0204	*NA*	
	2 - Low (>10 to ≤25th pctl)	537.3	280	52.1	1.08 (0.94–1.24)	0.2603	0.98 (0.86–1.12)	0.767	*NA*	
	3 - Medium (>25 to <75th pctl)	1,910.8	887	46.4	1.00 (Reference)		1.00 (Reference)		*NA*	
	4 - High (≥75 to <90th pctl)	630.0	276	43.8	0.99 (0.87–1.14)	0.9201	1.05 (0.91–1.20)	0.4974	*NA*	
	5 - Very high (≥90th pctl)	408.1	185	45.3	1.03 (0.88–1.20)	0.754	1.06 (0.91–1.25)	0.4571	*NA*	
Melanoma	1 - Very low (≤10th pctl)	989.7	45	4.5	1.33 (0.96–1.85)	0.0874	1.12 (0.80–1.57)	0.4969	1.06 (0.69–1.62)	0.7834
	2 - Low (>10 to ≤25th pctl)	1,683.0	41	2.4	0.77 (0.55–1.09)	0.1379	0.75 (0.53–1.05)	0.0965	0.58 (0.36–0.94)	0.0267
	3 - Medium (>25 to <75th pctl)	5,219.7	170	3.3	1.00 (Reference)		1.00 (Reference)		1.00 (Reference)	
	4 - High (≥75 to <90th pctl)	1,521.5	82	5.4	1.67 (1.29–2.18)	0.0001	1.79 (1.37–2.33)	<0.0001	1.45 (1.04–2.02)	0.029
	5 - Very high (≥90th pctl)	899.8	86	9.6	2.97 (2.29–3.85)	<0.0001	2.99 (2.30–3.88)	<0.0001	1.15 (0.81–1.63)	0.4196
Head and neck	1 - Very low (≤10th pctl)	904.8	60	6.6	1.11 (0.84–1.46)	0.4656	0.91 (0.69–1.21)	0.5207	1.04 (0.74–1.47)	0.8045
	2 - Low (>10 to ≤25th pctl)	1,489.8	69	4.6	0.79 (0.61–1.02)	0.0746	0.72 (0.55–0.93)	0.0129	0.84 (0.63–1.12)	0.2408
	3 - Medium (>25 to <75th pctl)	5,158.0	291	5.6	1.00 (Reference)		1.00 (Reference)		1.00 (Reference)	
	4 - High (≥75 to <90th pctl)	1,437.1	112	7.8	1.35 (1.09–1.68)	0.0065	1.41 (1.13–1.75)	0.0023	1.20 (0.94–1.55)	0.145
	5 - Very high (≥90th pctl)	717.5	110	15.3	2.43 (1.95–3.03)	<0.0001	2.49 (2.00–3.10)	<0.0001	2.13 (1.64–2.76)	<0.0001
Other solid tumour	1 - Very low (≤10th pctl)	1,370.5	354	25.8	1.10 (0.98–1.23)	0.1036	0.98 (0.87–1.10)	0.7386	1.04 (0.79–1.37)	0.7902
	2 - Low (>10 to ≤25th pctl)	2,653.1	501	18.9	0.88 (0.79–0.97)	0.0095	0.80 (0.72–0.88)	<0.0001	0.86 (0.70–1.06)	0.1678
	3 - Medium (>25 to <75th pctl)	8,195.0	1,798	21.9	1.00 (Reference)		1.00 (Reference)		1.00 (Reference)	
	4 - High (≥75 to <90th pctl)	1,726.9	715	41.4	1.61 (1.48–1.76)	<0.0001	1.58 (1.45–1.72)	<0.0001	1.34 (1.11–1.62)	0.0021
	5 - Very high (≥90th pctl)	934.4	547	58.5	2.10 (1.91–2.31)	<0.0001	2.15 (1.95–2.36)	<0.0001	1.26 (1.01–1.58)	0.0408

HR = Hazard ratio; pctl = percentile; NA = Not applicable; *SS* = data suppressed due to small number of observations/events. * Adjusted for age at diagnosis (cat.), year of diagnosis (cont.), and common chronic conditions (asthma, congestive heart failure, chronic obstructive pulmonary disease, hypertension, and diabetes). ** Adjusted for age at diagnosis (cat.), year of diagnosis (cont.), common chronic conditions (asthma, congestive heart failure, chronic obstructive pulmonary disease, hypertension, and diabetes), and cancer stage. Only subjects with known cancer stage were included.

Table S13. Cancer-specific mortality rates and hazard ratios by baseline platelet count category, by cancer site and stage.

						Unadjusted		Patient variable adjustment*	
Cancer	Stage	Platelet category	Person-years	Cancer-specific deaths	Rate (% per year)	HR (95% CI)	P-value	HR (95% CI)	P-value
Colon	Stage I/II	1 - Low (≤25th pctl)	7,903.0	174	2.2	0.84 (0.70–1.00)	0.0525	0.83 (0.69–1.00)	0.0481
		2 - Medium (>25 to <75th pctl)	13,884.1	359	2.6	1.00 (Reference)		1.00 (Reference)	
		3 - High (≥75th pctl)	5,339.5	235	4.4	1.69 (1.43–1.99)	<0.0001	1.64 (1.39–1.93)	<0.0001
	Stage III/IV	1 - Low (≤25th pctl)	4,713.3	628	13.3	0.76 (0.69–0.83)	<0.0001	0.75 (0.68–0.82)	<0.0001
		2 - Medium (>25 to <75th pctl)	10,837.4	1,890	17.4	1.00 (Reference)		1.00 (Reference)	
		3 - High (≥75th pctl)	5,211.6	1,457	28.0	1.52 (1.42–1.63)	<0.0001	1.55 (1.45–1.66)	<0.0001
Lung	Stage I/II	1 - Low (≤25th pctl)	5,134.4	367	7.1	0.85 (0.74–0.96)	0.0113	0.77 (0.68–0.88)	0.0001
		2 - Medium (>25 to <75th pctl)	7,166.3	598	8.3	1.00 (Reference)		1.00 (Reference)	
		3 - High (≥75th pctl)	1,615.8	217	13.4	1.61 (1.38–1.88)	<0.0001	1.58 (1.35–1.84)	<0.0001
	Stage III/IV	1 - Low (≤25th pctl)	2,855.5	2,105	73.7	0.98 (0.93–1.03)	0.5141	0.92 (0.87–0.97)	0.0013
		2 - Medium (>25 to <75th pctl)	7,040.7	5,319	75.5	1.00 (Reference)		1.00 (Reference)	
		3 - High (≥75th pctl)	3,072.5	3,298	107.3	1.31 (1.26–1.37)	<0.0001	1.33 (1.28–1.39)	<0.0001
Breast	Stage I/II	1 - Low (≤25th pctl)	7,604.1	91	1.2	1.54 (1.17–2.02)	0.002	1.35 (1.03–1.78)	0.0297
		2 - Medium (>25 to <75th pctl)	15,684.7	122	0.8	1.00 (Reference)		1.00 (Reference)	
		3 - High (≥75th pctl)	6,960.8	80	1.1	1.48 (1.12–1.96)	0.0063	1.54 (1.16–2.05)	0.0027
	Stage III/IV	1 - Low (≤25th pctl)	1,452.0	166	11.4	1.10 (0.92–1.33)	0.2955	1.02 (0.85–1.23)	0.8266
		2 - Medium (>25 to <75th pctl)	3,434.2	348	10.1	1.00 (Reference)		1.00 (Reference)	
		3 - High (≥75th pctl)	2,073.0	306	14.8	1.43 (1.22–1.67)	<0.0001	1.43 (1.23–1.67)	<0.0001
Prostate	Stage I/II	1 - Low (≤25th pctl)	5,334.1	48	0.9	1.41 (0.99–2.03)	0.0603	1.26 (0.88–1.82)	0.205
		2 - Medium (>25 to <75th pctl)	11,967.9	77	0.6	1.00 (Reference)		1.00 (Reference)	
		3 - High (≥75th pctl)	5,276.6	58	1.1	1.70 (1.21–2.39)	0.0023	1.77 (1.26–2.49)	0.0011
	Stage III/IV	1 - Low (≤25th pctl)	1,586.4	243	15.3	1.68 (1.42–1.99)	<0.0001	1.33 (1.12–1.58)	0.0009
		2 - Medium (>25 to <75th pctl)	3,675.6	318	8.7	1.00 (Reference)		1.00 (Reference)	
		3 - High (≥75th pctl)	2,019.5	305	15.1	1.68 (1.44–1.97)	<0.0001	1.57 (1.34–1.84)	<0.0001
Ovary	Stage I/II	1 - Low (≤25th pctl)	864.7	14	1.6	0.73 (0.38–1.38)	0.3267	0.70 (0.37–1.34)	0.2818
		2 - Medium (>25 to <75th pctl)	1,315.1	29	2.2	1.00 (Reference)		1.00 (Reference)	
		3 - High (≥75th pctl)	314.4	21	6.7	3.01 (1.71–5.30)	0.0001	3.10 (1.74–5.52)	0.0001
	Stage III/IV	1 - Low (≤25th pctl)	634.0	116	18.3	0.82 (0.67–1.01)	0.0617	0.76 (0.62–0.94)	0.0124
		2 - Medium (>25 to <75th pctl)	1,751.5	392	22.4	1.00 (Reference)		1.00 (Reference)	
		3 - High (≥75th pctl)	1,028.4	289	28.1	1.25 (1.08–1.46)	0.0034	1.30 (1.12–1.52)	0.0007
Stomach	Stage I/II	1 - Low (≤25th pctl)	321.7	37	11.5	1.07 (0.71–1.60)	0.7404	0.92 (0.60–1.41)	0.7021
		2 - Medium (>25 to <75th pctl)	649.7	65	10.0	1.00 (Reference)		1.00 (Reference)	

	3 - High (≥75th pctl)	249.2	21	8.4	0.92 (0.56–1.50)	0.7321	0.73 (0.44–1.21)	0.2161
Stage III/IV	1 - Low (≤25th pctl)	259.6	150	57.8	1.03 (0.86–1.25)	0.7246	1.03 (0.85–1.25)	0.7462
	2 - Medium (>25 to <75th pctl)	668.1	382	57.2	1.00 (Reference)		1.00 (Reference)	
	3 - High (≥75th pctl)	329.9	218	66.1	1.14 (0.96–1.34)	0.1316	1.16 (0.98–1.37)	0.0881

* Adjusted for age at diagnosis (cat.), year of diagnosis (cont.), and common chronic conditions (asthma, congestive heart failure, chronic obstructive pulmonary disease, hypertension, and diabetes).

Table S14. Cancer-specific mortality rates and hazard ratios by time-dependent platelet count category, for select cancers.

					Unadjusted		Patient variable adjustment *		Patient variable, cancer stage, and cancer treatment adjustment**	
**Cancer**	**Platelet category**	**Person-years**	**Cancer-specific deaths**	**Rate (% per year)**	**HR (95% CI)**	**P-value**	**HR (95% CI)**	**P-value**	**HR (95% CI)**	**P-value**
All cancers	1 - Low (≤25th pctl)	102,159.7	10,826	10.6	1.01 (0.98–1.03)	0.5642	0.95 (0.92–0.97)	<0.0001	*NA*	
	2 - Medium (>25 to <75th pctl)	141,620.0	16,661	11.8	1.00 (Reference)		1.00 (Reference)		*NA*	
	3 - High (≥75th pctl)	51,479.6	12,842	24.9	1.79 (1.75–1.83)	<0.0001	1.83 (1.79–1.88)	<0.0001	*NA*	
Colon	1 - Low (≤25th pctl)	24,233.9	1,330	5.5	0.61 (0.57–0.65)	<0.0001	0.61 (0.57–0.65)	<0.0001	0.57 (0.52–0.62)	<0.0001
	2 - Medium (>25 to <75th pctl)	21,480.1	2,312	10.8	1.00 (Reference)		1.00 (Reference)		1.00 (Reference)	
	3 - High (≥75th pctl)	4,877.6	1,678	34.4	2.30 (2.16–2.45)	<0.0001	2.32 (2.18–2.48)	<0.0001	1.76 (1.62–1.92)	<0.0001
Lung	1 - Low (≤25th pctl)	9,957.4	3,252	32.7	0.86 (0.83–0.90)	<0.0001	0.83 (0.79–0.87)	<0.0001	0.90 (0.86–0.95)	<0.0001
	2 - Medium (>25 to <75th pctl)	14,057.4	5,943	42.3	1.00 (Reference)		1.00 (Reference)		1.00 (Reference)	
	3 - High (≥75th pctl)	4,337.5	3,979	91.7	1.72 (1.65–1.79)	<0.0001	1.75 (1.68–1.83)	<0.0001	1.48 (1.41–1.54)	<0.0001
Breast	1 - Low (≤25th pctl)	12,565.1	405	3.2	1.46 (1.27–1.67)	<0.0001	1.36 (1.18–1.55)	<0.0001	1.30 (1.12–1.51)	0.0006
	2 - Medium (>25 to <75th pctl)	18,594.0	426	2.3	1.00 (Reference)		1.00 (Reference)		1.00 (Reference)	
	3 - High (≥75th pctl)	7,399.9	411	5.6	2.29 (2.00–2.63)	<0.0001	2.35 (2.05–2.69)	<0.0001	1.85 (1.59–2.15)	<0.0001
Prostate	1 - Low (≤25th pctl)	8,582.4	396	4.6	1.84 (1.60–2.11)	<0.0001	1.60 (1.39–1.84)	<0.0001	1.69 (1.43–2.00)	<0.0001
	2 - Medium (>25 to <75th pctl)	16,723.2	418	2.5	1.00 (Reference)		1.00 (Reference)		1.00 (Reference)	
	3 - High (≥75th pctl)	7,189.0	459	6.4	2.45 (2.15–2.80)	<0.0001	2.40 (2.10–2.74)	<0.0001	1.93 (1.65–2.27)	<0.0001
Ovary	1 - Low (≤25th pctl)	4,054.0	416	10.3	0.63 (0.55–0.71)	<0.0001	0.60 (0.52–0.68)	<0.0001	0.58 (0.48–0.70)	<0.0001
	2 - Medium (>25 to <75th pctl)	3,046.8	548	18.0	1.00 (Reference)		1.00 (Reference)		1.00 (Reference)	
	3 - High (≥75th pctl)	778.0	371	47.7	2.22 (1.94–2.54)	<0.0001	2.29 (2.00–2.62)	<0.0001	2.39 (1.93–2.95)	<0.0001
Stomach	1 - Low (≤25th pctl)	2,266.9	603	26.6	0.85 (0.77–0.94)	0.0022	0.84 (0.76–0.93)	0.0012	0.87 (0.73–1.04)	0.1174

2 - Medium (>25 to <75th pctl)	2,416.1	908	37.6	1.00 (Reference)		1.00 (Reference)		1.00 (Reference)	
3 - High (≥75th pctl)	694.9	576	82.9	1.62 (1.46–1.80)	<0.0001	1.61 (1.45–1.79)	<0.0001	1.28 (1.07–1.53)	0.0077

HR = Hazard ratio; pctl = percentile. * Adjusted for age at diagnosis (cat.), year of diagnosis (cont.), and common chronic conditions (asthma, congestive heart failure, chronic obstructive pulmonary disease, hypertension, and diabetes). ** Adjusted for age at diagnosis (cat.), year of diagnosis (cont.), common chronic conditions (asthma, congestive heart failure, chronic obstructive pulmonary disease, hypertension, and diabetes), cancer stage, chemotherapy, and radiotherapy. Time-dependent confounding adjustment using marginal structural models. Only subjects with known cancer stage were included.

Table S15. Cancer-specific mortality rates and hazard ratios by time-dependent platelet count category (exposure definition 1).

		**Unadjusted**		**Patient variable adjustment***		**Patient variable and cancer stage adjustment****		**Patient variable, cancer stage, and cancer treatment adjustment*****	
**Cancer**	**Platelet category**	**HR (95% CI)**	**P-value**	**HR (95% CI)**	**P-value**	**HR (95% CI)**	**P-value**	**HR (95% CI)**	**P-value**
All cancers	1 - Low (≤25th pctl)	1.01 (0.98–1.03)	0.5642	0.95 (0.92–0.97)	<0.0001	0.89 (0.86–0.92)	<0.0001	*NA*	
	2 - Medium (>25 to <75th pctl)	1.00 (Reference)		1.00 (Reference)		1.00 (Reference)		*NA*	
	3 - High (≥75th pctl)	1.79 (1.75–1.83)	<0.0001	1.83 (1.79–1.88)	<0.0001	1.51 (1.47–1.56)	<0.0001	*NA*	
Colon	1 - Low (≤25th pctl)	0.61 (0.57–0.65)	<0.0001	0.61 (0.57–0.65)	<0.0001	0.63 (0.59–0.68)	<0.0001	0.57 (0.52–0.62)	<0.0001
	2 - Medium (>25 to <75th pctl)	1.00 (Reference)		1.00 (Reference)		1.00 (Reference)		1.00 (Reference)	
	3 - High (≥75th pctl)	2.30 (2.16–2.45)	<0.0001	2.32 (2.18–2.48)	<0.0001	1.72 (1.61–1.84)	<0.0001	1.76 (1.62–1.92)	<0.0001
Lung	1 - Low (≤25th pctl)	0.86 (0.83–0.90)	<0.0001	0.83 (0.79–0.87)	<0.0001	0.91 (0.87–0.95)	<0.0001	0.90 (0.86–0.95)	<0.0001
	2 - Medium (>25 to <75th pctl)	1.00 (Reference)		1.00 (Reference)		1.00 (Reference)		1.00 (Reference)	
	3 - High (≥75th pctl)	1.72 (1.65–1.79)	<0.0001	1.75 (1.68–1.83)	<0.0001	1.48 (1.42–1.55)	<0.0001	1.48 (1.41–1.54)	<0.0001
Breast	1 - Low (≤25th pctl)	1.46 (1.27–1.67)	<0.0001	1.36 (1.18–1.55)	<0.0001	1.32 (1.14–1.52)	0.0002	1.30 (1.12–1.51)	0.0006
	2 - Medium (>25 to <75th pctl)	1.00 (Reference)		1.00 (Reference)		1.00 (Reference)		1.00 (Reference)	
	3 - High (≥75th pctl)	2.29 (2.00–2.63)	<0.0001	2.35 (2.05–2.69)	<0.0001	1.87 (1.62–2.16)	<0.0001	1.85 (1.59–2.15)	<0.0001
Prostate	1 - Low (≤25th pctl)	1.84 (1.60–2.11)	<0.0001	1.60 (1.39–1.84)	<0.0001	1.69 (1.45–1.97)	<0.0001	1.69 (1.43–2.00)	<0.0001
	2 - Medium (>25 to <75th pctl)	1.00 (Reference)		1.00 (Reference)		1.00 (Reference)		1.00 (Reference)	
	3 - High (≥75th pctl)	2.45 (2.15–2.80)	<0.0001	2.40 (2.10–2.74)	<0.0001	1.98 (1.70–2.29)	<0.0001	1.93 (1.65–2.27)	<0.0001
Ovary	1 - Low (≤25th pctl)	0.63 (0.55–0.71)	<0.0001	0.60 (0.52–0.68)	<0.0001	0.61 (0.52–0.72)	<0.0001	0.58 (0.48–0.70)	<0.0001
	2 - Medium (>25 to <75th pctl)	1.00 (Reference)		1.00 (Reference)		1.00 (Reference)		1.00 (Reference)	
	3 - High (≥75th pctl)	2.22 (1.94–2.54)	<0.0001	2.29 (2.00–2.62)	<0.0001	2.18 (1.83–2.59)	<0.0001	2.39 (1.93–2.95)	<0.0001
Stomach	1 - Low (≤25th pctl)	0.85 (0.77–0.94)	0.0022	0.84 (0.76–0.93)	0.0012	0.89 (0.76–1.05)	0.1696	0.87 (0.73–1.04)	0.1174
	2 - Medium (>25 to <75th pctl)	1.00 (Reference)		1.00 (Reference)		1.00 (Reference)		1.00 (Reference)	
	3 - High (≥75th pctl)	1.62 (1.46–1.80)	<0.0001	1.61 (1.45–1.79)	<0.0001	1.29 (1.09–1.53)	0.0026	1.28 (1.07–1.53)	0.0077
Pancreas	1 - Low (≤25th pctl)	1.08 (1.00–1.16)	0.0474	1.08 (1.00–1.16)	0.0418	0.91 (0.82–1.02)	0.1014	0.87 (0.77–0.99)	0.0335
	2 - Medium (>25 to <75th pctl)	1.00 (Reference)		1.00 (Reference)		1.00 (Reference)		1.00 (Reference)	
	3 - High (≥75th pctl)	1.15 (1.07–1.24)	0.0002	1.20 (1.11–1.29)	<0.0001	1.21 (1.08–1.35)	0.0008	1.17 (1.04–1.32)	0.0112
Kidney	1 - Low (≤25th pctl)	1.11 (0.93–1.31)	0.2464	0.98 (0.82–1.16)	0.8091	0.99 (0.78–1.25)	0.9354	0.95 (0.73–1.25)	0.7255
	2 - Medium (>25 to <75th pctl)	1.00 (Reference)		1.00 (Reference)		1.00 (Reference)		1.00 (Reference)	
	3 - High (≥75th pctl)	3.94 (3.42–4.54)	<0.0001	3.82 (3.31–4.40)	<0.0001	2.31 (1.89–2.82)	<0.0001	2.42 (1.94–3.02)	<0.0001

Bladder	1 - Low (≤25th pctl)	1.01 (0.85–1.20)	0.9057	0.87 (0.73–1.04)	0.1177	0.79 (0.60–1.06)	0.1125	0.78 (0.57–1.08)	0.1308
	2 - Medium (>25 to <75th pctl)	1.00 (Reference)		1.00 (Reference)		1.00 (Reference)		1.00 (Reference)	
	3 - High (≥75th pctl)	3.45 (3.03–3.93)	<0.0001	3.49 (3.06–3.98)	<0.0001	2.29 (1.84–2.85)	<0.0001	2.21 (1.76–2.78)	<0.0001
Esophagus	1 - Low (≤25th pctl)	0.99 (0.85–1.14)	0.865	0.97 (0.84–1.12)	0.6723	0.85 (0.69–1.03)	0.0998	0.87 (0.70–1.08)	0.1979
	2 - Medium (>25 to <75th pctl)	1.00 (Reference)		1.00 (Reference)		1.00 (Reference)		1.00 (Reference)	
	3 - High (≥75th pctl)	1.74 (1.50–2.01)	<0.0001	1.79 (1.55–2.07)	<0.0001	1.38 (1.13–1.69)	0.0014	1.42 (1.13–1.78)	0.0025
Other GI	1 - Low (≤25th pctl)	0.97 (0.80–1.18)	0.7651	0.95 (0.78–1.15)	0.5731	0.85 (0.62–1.16)	0.2995	0.71 (0.51–1.00)	0.0505
	2 - Medium (>25 to <75th pctl)	1.00 (Reference)		1.00 (Reference)		1.00 (Reference)		1.00 (Reference)	
	3 - High (≥75th pctl)	2.13 (1.77–2.56)	<0.0001	2.17 (1.81–2.61)	<0.0001	1.86 (1.38–2.51)	<0.0001	1.76 (1.26–2.45)	0.0009
Thyroid	1 - Low (≤25th pctl)	1.47 (0.89–2.41)	0.13	1.22 (0.74–2.01)	0.4353	0.62 (0.30–1.26)	0.1842	0.58 (0.24–1.41)	0.2319
	2 - Medium (>25 to <75th pctl)	1.00 (Reference)		1.00 (Reference)		1.00 (Reference)		1.00 (Reference)	
	3 - High (≥75th pctl)	2.36 (1.53–3.64)	<0.0001	2.82 (1.82–4.36)	<0.0001	1.43 (0.82–2.47)	0.2074	1.41 (0.76–2.62)	0.2804
Cervix	1 - Low (≤25th pctl)	0.70 (0.50–0.99)	0.0418	0.64 (0.45–0.91)	0.0119	0.67 (0.47–0.96)	0.0313	0.62 (0.33–1.16)	0.1353
	2 - Medium (>25 to <75th pctl)	1.00 (Reference)		1.00 (Reference)		1.00 (Reference)		1.00 (Reference)	
	3 - High (≥75th pctl)	3.41 (2.55–4.56)	<0.0001	3.43 (2.56–4.58)	<0.0001	2.15 (1.57–2.95)	<0.0001	1.90 (1.20–3.02)	0.0062
Testis	1 - Low (≤25th pctl)	1.10 (0.38–3.17)	0.861	0.94 (0.32–2.77)	0.9119	0.41 (0.11–1.57)	0.1925	1.56 (0.61–4.01)	0.3574
	2 - Medium (>25 to <75th pctl)	1.00 (Reference)		1.00 (Reference)		1.00 (Reference)		1.00 (Reference)	
	3 - High (≥75th pctl)	2.72 (1.05–7.06)	0.0396	2.53 (0.95–6.71)	0.0623	0.46 (0.13–1.69)	0.245	0.30 (0.05–1.74)	0.1801
Endometrium	1 - Low (≤25th pctl)	1.52 (1.22–1.88)	0.0002	1.31 (1.05–1.63)	0.0155	1.24 (0.96–1.60)	0.1048	1.21 (0.86–1.69)	0.2725
	2 - Medium (>25 to <75th pctl)	1.00 (Reference)		1.00 (Reference)		1.00 (Reference)		1.00 (Reference)	
	3 - High (≥75th pctl)	4.56 (3.77–5.52)	<0.0001	4.89 (4.04–5.92)	<0.0001	3.45 (2.73–4.37)	<0.0001	3.96 (2.90–5.41)	<0.0001
Brain	1 - Low (≤25th pctl)	1.44 (1.30–1.60)	<0.0001	1.29 (1.16–1.43)	<0.0001	*NA*		*NA*	
	2 - Medium (>25 to <75th pctl)	1.00 (Reference)		1.00 (Reference)		*NA*		*NA*	
	3 - High (≥75th pctl)	1.16 (1.02–1.32)	0.0227	1.20 (1.05–1.36)	0.0071	*NA*		*NA*	
Melanoma	1 - Low (≤25th pctl)	1.07 (0.82–1.41)	0.6191	0.97 (0.74–1.28)	0.8508	0.84 (0.58–1.20)	0.337	0.78 (0.53–1.16)	0.222
	2 - Medium (>25 to <75th pctl)	1.00 (Reference)		1.00 (Reference)		1.00 (Reference)		1.00 (Reference)	
	3 - High (≥75th pctl)	3.10 (2.50–3.84)	<0.0001	3.22 (2.60–4.00)	<0.0001	2.34 (1.79–3.06)	<0.0001	2.28 (1.68–3.08)	<0.0001
Head and neck	1 - Low (≤25th pctl)	0.84 (0.68–1.03)	0.0991	0.76 (0.61–0.94)	0.0106	0.74 (0.58–0.94)	0.0146	0.70 (0.54–0.91)	0.0071
	2 - Medium (>25 to <75th pctl)	1.00 (Reference)		1.00 (Reference)		1.00 (Reference)		1.00 (Reference)	
	3 - High (≥75th pctl)	2.32 (1.95–2.76)	<0.0001	2.36 (1.98–2.81)	<0.0001	2.18 (1.78–2.66)	<0.0001	2.12 (1.71–2.62)	<0.0001
Other solid tumour	1 - Low (≤25th pctl)	1.05 (0.97–1.13)	0.2735	0.97 (0.89–1.05)	0.3829	0.96 (0.81–1.14)	0.6603	0.95 (0.79–1.14)	0.5781
	2 - Medium (>25 to <75th pctl)	1.00 (Reference)		1.00 (Reference)		1.00 (Reference)		1.00 (Reference)	
	3 - High (≥75th pctl)	2.09 (1.94–2.25)	<0.0001	2.10 (1.95–2.26)	<0.0001	1.90 (1.62–2.22)	<0.0001	1.88 (1.57–2.24)	<0.0001

HR = Hazard ratio; pctl = percentile; NA = Not applicable; *SS* = data suppressed due to small number of observations/events. * Adjusted for age at diagnosis (cat.), year of diagnosis (cont.), and common chronic conditions (asthma, congestive heart failure, chronic obstructive pulmonary disease, hypertension, and diabetes). ** Adjusted for age at diagnosis (cat.), year of diagnosis (cont.), common chronic conditions (asthma, congestive heart failure, chronic obstructive pulmonary disease, hypertension, and diabetes), and cancer stage. Only subjects with known cancer stage were included in the analysis. *** Adjusted for age at diagnosis (cat.), year of diagnosis (cont.), common chronic conditions (asthma, congestive heart failure, chronic obstructive pulmonary disease, hypertension, and diabetes), cancer stage, chemotherapy, and radiotherapy. Only subjects with known cancer stage were included in the analysis. Time-dependent confounding adjustment using marginal structural models.

Table S16. Cancer-specific mortality rates and hazard ratios by time-dependent platelet count category (exposure definition 2).

		Unadjusted		Patient variable adjustment*		Patient variable and cancer stage adjustment**		Patient variable, cancer stage, and cancer treatment adjustment***	
**Cancer**	**Platelet category**	**HR (95% CI)**	**P-value**	**HR (95% CI)**	**P-value**	**HR (95% CI)**	**P-value**	**HR (95% CI)**	**P-value**
All cancers	1 - Very low (≤10th pctl)	1.20 (1.17–1.24)	<0.0001	1.11 (1.08–1.15)	<0.0001	0.99 (0.95–1.02)	0.4335	*NA*	
	2 - Low (>10 to ≤25th pctl)	0.85 (0.82–0.88)	<0.0001	0.81 (0.78–0.84)	<0.0001	0.80 (0.77–0.84)	<0.0001	*NA*	
	3 - Medium (>25 to <75th pctl)	1.00 (Reference)		1.00 (Reference)		1.00 (Reference)		*NA*	
	4 - High (≥75 to <90th pctl)	1.51 (1.46–1.55)	<0.0001	1.54 (1.50–1.58)	<0.0001	1.37 (1.32–1.41)	<0.0001	*NA*	
	5 - Very high (≥90th pctl)	2.23 (2.17–2.30)	<0.0001	2.31 (2.24–2.38)	<0.0001	1.71 (1.65–1.77)	<0.0001	*NA*	
Colon	1 - Very low (≤10th pctl)	0.67 (0.62–0.73)	<0.0001	0.67 (0.61–0.72)	<0.0001	0.65 (0.59–0.71)	<0.0001	0.63 (0.57–0.70)	<0.0001
	2 - Low (>10 to ≤25th pctl)	0.55 (0.51–0.61)	<0.0001	0.55 (0.50–0.60)	<0.0001	0.61 (0.56–0.67)	<0.0001	0.61 (0.55–0.67)	<0.0001
	3 - Medium (>25 to <75th pctl)	1.00 (Reference)		1.00 (Reference)		1.00 (Reference)		1.00 (Reference)	
	4 - High (≥75 to <90th pctl)	2.01 (1.86–2.17)	<0.0001	2.00 (1.85–2.16)	<0.0001	1.60 (1.47–1.74)	<0.0001	1.59 (1.46–1.74)	<0.0001
	5 - Very high (≥90th pctl)	2.80 (2.57–3.04)	<0.0001	2.90 (2.67–3.15)	<0.0001	1.90 (1.74–2.08)	<0.0001	1.89 (1.70–2.11)	<0.0001
Lung	1 - Very low (≤10th pctl)	1.00 (0.95–1.06)	0.9609	0.95 (0.89–1.00)	0.0491	1.02 (0.96–1.08)	0.5627	1.01 (0.95–1.08)	0.6733
	2 - Low (>10 to ≤25th pctl)	0.76 (0.72–0.81)	<0.0001	0.74 (0.70–0.78)	<0.0001	0.82 (0.78–0.87)	<0.0001	0.82 (0.77–0.87)	<0.0001
	3 - Medium (>25 to <75th pctl)	1.00 (Reference)		1.00 (Reference)		1.00 (Reference)		1.00 (Reference)	
	4 - High (≥75 to <90th pctl)	1.56 (1.49–1.64)	<0.0001	1.58 (1.50–1.66)	<0.0001	1.37 (1.30–1.44)	<0.0001	1.37 (1.29–1.44)	<0.0001
	5 - Very high (≥90th pctl)	1.96 (1.86–2.07)	<0.0001	2.03 (1.93–2.15)	<0.0001	1.65 (1.56–1.75)	<0.0001	1.65 (1.55–1.75)	<0.0001
Breast	1 - Very low (≤10th pctl)	2.07 (1.77–2.42)	<0.0001	1.88 (1.60–2.20)	<0.0001	1.66 (1.40–1.96)	<0.0001	1.62 (1.37–1.93)	<0.0001
	2 - Low (>10 to ≤25th pctl)	1.01 (0.84–1.21)	0.8956	0.96 (0.80–1.15)	0.6489	1.01 (0.83–1.22)	0.9447	1.00 (0.82–1.21)	0.967
	3 - Medium (>25 to <75th pctl)	1.00 (Reference)		1.00 (Reference)		1.00 (Reference)		1.00 (Reference)	
	4 - High (≥75 to <90th pctl)	1.47 (1.23–1.77)	<0.0001	1.52 (1.27–1.82)	<0.0001	1.41 (1.16–1.71)	0.0005	1.38 (1.13–1.68)	0.0013
	5 - Very high (≥90th pctl)	3.62 (3.09–4.24)	<0.0001	3.65 (3.12–4.28)	<0.0001	2.40 (2.03–2.84)	<0.0001	2.35 (1.94–2.84)	<0.0001
Prostate	1 - Very low (≤10th pctl)	2.99 (2.55–3.50)	<0.0001	2.46 (2.10–2.88)	<0.0001	2.48 (2.08–2.96)	<0.0001	2.37 (1.94–2.90)	<0.0001
	2 - Low (>10 to ≤25th pctl)	1.12 (0.93–1.35)	0.2304	1.02 (0.85–1.23)	0.8208	1.15 (0.94–1.41)	0.1768	1.17 (0.95–1.45)	0.1431
	3 - Medium (>25 to <75th pctl)	1.00 (Reference)		1.00 (Reference)		1.00 (Reference)		1.00 (Reference)	
	4 - High (≥75 to <90th pctl)	1.75 (1.48–2.07)	<0.0001	1.74 (1.48–2.06)	<0.0001	1.51 (1.26–1.82)	<0.0001	1.45 (1.19–1.75)	0.0002
	5 - Very high (≥90th pctl)	3.73 (3.19–4.36)	<0.0001	3.53 (3.02–4.14)	<0.0001	2.71 (2.28–3.23)	<0.0001	2.68 (2.21–3.25)	<0.0001
Ovary	1 - Very low (≤10th pctl)	0.66 (0.57–0.77)	<0.0001	0.61 (0.52–0.71)	<0.0001	0.58 (0.48–0.71)	<0.0001	0.57 (0.46–0.71)	<0.0001
	2 - Low (>10 to ≤25th pctl)	0.58 (0.49–0.69)	<0.0001	0.58 (0.49–0.69)	<0.0001	0.65 (0.52–0.80)	<0.0001	0.64 (0.52–0.80)	0.0001
	3 - Medium (>25 to <75th pctl)	1.00 (Reference)		1.00 (Reference)		1.00 (Reference)		1.00 (Reference)	
	4 - High (≥75 to <90th pctl)	2.13 (1.81–2.50)	<0.0001	2.13 (1.81–2.51)	<0.0001	2.12 (1.72–2.60)	<0.0001	2.09 (1.66–2.63)	<0.0001
	5 - Very high (≥90th pctl)	2.36 (1.98–2.82)	<0.0001	2.53 (2.12–3.03)	<0.0001	2.27 (1.81–2.86)	<0.0001	2.28 (1.75–2.96)	<0.0001
Stomach	1 - Very low (≤10th pctl)	0.88 (0.77–1.01)	0.0627	0.86 (0.76–0.99)	0.0301	0.93 (0.76–1.14)	0.4691	0.92 (0.75–1.12)	0.3972
	2 - Low (>10 to ≤25th pctl)	0.82 (0.72–0.94)	0.0031	0.82 (0.72–0.93)	0.003	0.86 (0.70–1.06)	0.1488	0.86 (0.70–1.06)	0.1462
	3 - Medium (>25 to <75th pctl)	1.00 (Reference)		1.00 (Reference)		1.00 (Reference)		1.00 (Reference)	

	4 - High (≥75 to <90th pctl)	1.57 (1.39–1.78)	<0.0001	1.55 (1.37–1.77)	<0.0001	1.42 (1.16–1.73)	0.0006	1.40 (1.13–1.74)	0.0018
	5 - Very high (≥90th pctl)	1.69 (1.47–1.95)	<0.0001	1.69 (1.47–1.95)	<0.0001	1.15 (0.91–1.44)	0.234	1.14 (0.88–1.47)	0.3184
Pancreas	1 - Very low (≤10th pctl)	1.16 (1.06–1.27)	0.0009	1.17 (1.07–1.28)	0.0008	0.93 (0.81–1.06)	0.2527	0.88 (0.74–1.05)	0.1465
	2 - Low (>10 to ≤25th pctl)	0.99 (0.91–1.09)	0.9044	1.00 (0.91–1.09)	0.9333	0.90 (0.78–1.03)	0.1378	0.92 (0.79–1.08)	0.3202
	3 - Medium (>25 to <75th pctl)	1.00 (Reference)		1.00 (Reference)		1.00 (Reference)		1.00 (Reference)	
	4 - High (≥75 to <90th pctl)	1.11 (1.02–1.22)	0.0225	1.15 (1.04–1.26)	0.004	1.18 (1.03–1.35)	0.0198	1.15 (0.99–1.33)	0.0716
	5 - Very high (≥90th pctl)	1.20 (1.09–1.32)	0.0002	1.26 (1.15–1.39)	<0.0001	1.25 (1.08–1.44)	0.0025	1.21 (1.04–1.41)	0.0158
Kidney	1 - Very low (≤10th pctl)	1.49 (1.22–1.82)	0.0001	1.29 (1.05–1.57)	0.0148	1.29 (0.98–1.69)	0.0733	1.24 (0.89–1.71)	0.1998
	2 - Low (>10 to ≤25th pctl)	0.79 (0.63–1.00)	0.0542	0.72 (0.56–0.91)	0.0057	0.72 (0.52–1.00)	0.0492	0.73 (0.51–1.06)	0.0956
	3 - Medium (>25 to <75th pctl)	1.00 (Reference)		1.00 (Reference)		1.00 (Reference)		1.00 (Reference)	
	4 - High (≥75 to <90th pctl)	2.66 (2.24–3.17)	<0.0001	2.61 (2.20–3.11)	<0.0001	2.01 (1.58–2.55)	<0.0001	2.15 (1.65–2.79)	<0.0001
	5 - Very high (≥90th pctl)	6.42 (5.45–7.56)	<0.0001	6.08 (5.16–7.17)	<0.0001	2.65 (2.10–3.34)	<0.0001	2.68 (2.06–3.50)	<0.0001
Bladder	1 - Very low (≤10th pctl)	1.30 (1.05–1.61)	0.0166	1.07 (0.86–1.32)	0.5586	0.76 (0.51–1.11)	0.1579	0.82 (0.49–1.37)	0.4421
	2 - Low (>10 to ≤25th pctl)	0.81 (0.64–1.01)	0.0622	0.72 (0.58–0.90)	0.0047	0.83 (0.58–1.16)	0.2746	0.83 (0.57–1.23)	0.3582
	3 - Medium (>25 to <75th pctl)	1.00 (Reference)		1.00 (Reference)		1.00 (Reference)		1.00 (Reference)	
	4 - High (≥75 to <90th pctl)	2.22 (1.88–2.61)	<0.0001	2.27 (1.92–2.67)	<0.0001	1.84 (1.40–2.41)	<0.0001	1.84 (1.37–2.46)	<0.0001
	5 - Very high (≥90th pctl)	5.58 (4.82–6.47)	<0.0001	5.56 (4.80–6.45)	<0.0001	2.71 (2.13–3.45)	<0.0001	2.59 (1.99–3.36)	<0.0001
Esophagus	1 - Very low (≤10th pctl)	0.94 (0.78–1.13)	0.5354	0.91 (0.75–1.09)	0.3039	0.78 (0.61–1.00)	0.054	0.82 (0.63–1.07)	0.1406
	2 - Low (>10 to ≤25th pctl)	1.03 (0.86–1.24)	0.719	1.04 (0.86–1.24)	0.7127	0.93 (0.72–1.19)	0.5518	0.98 (0.74–1.31)	0.9019
	3 - Medium (>25 to <75th pctl)	1.00 (Reference)		1.00 (Reference)		1.00 (Reference)		1.00 (Reference)	
	4 - High (≥75 to <90th pctl)	1.52 (1.27–1.82)	<0.0001	1.57 (1.31–1.88)	<0.0001	1.22 (0.95–1.58)	0.1161	1.30 (0.98–1.71)	0.0645
	5 - Very high (≥90th pctl)	2.05 (1.71–2.47)	<0.0001	2.12 (1.76–2.55)	<0.0001	1.60 (1.24–2.06)	0.0003	1.57 (1.15–2.15)	0.0046
Other GI	1 - Very low (≤10th pctl)	1.22 (0.96–1.55)	0.0959	1.20 (0.94–1.52)	0.1408	1.10 (0.76–1.59)	0.6226	1.07 (0.73–1.57)	0.7339
	2 - Low (>10 to ≤25th pctl)	0.78 (0.60–1.00)	0.0528	0.75 (0.58–0.97)	0.0312	0.63 (0.41–0.97)	0.0342	0.63 (0.41–0.96)	0.0325
	3 - Medium (>25 to <75th pctl)	1.00 (Reference)		1.00 (Reference)		1.00 (Reference)		1.00 (Reference)	
	4 - High (≥75 to <90th pctl)	1.89 (1.51–2.37)	<0.0001	1.95 (1.55–2.44)	<0.0001	1.97 (1.38–2.81)	0.0002	1.95 (1.32–2.88)	0.0009
	5 - Very high (≥90th pctl)	2.46 (1.96–3.09)	<0.0001	2.47 (1.96–3.10)	<0.0001	1.72 (1.16–2.54)	0.0066	1.70 (1.14–2.55)	0.0099
Thyroid	1 - Very low (≤10th pctl)	1.96 (1.07–3.57)	0.0281	1.46 (0.80–2.67)	0.2203	0.61 (0.26–1.41)	0.2469	0.54 (0.19–1.50)	0.2354
	2 - Low (>10 to ≤25th pctl)	1.12 (0.58–2.14)	0.7395	1.01 (0.53–1.94)	0.9799	0.66 (0.25–1.79)	0.4152	0.73 (0.23–2.28)	0.5897
	3 - Medium (>25 to <75th pctl)	1.00 (Reference)		1.00 (Reference)		1.00 (Reference)		1.00 (Reference)	
	4 - High (≥75 to <90th pctl)	0.76 (0.37–1.58)	0.469	0.96 (0.46–1.99)	0.9061	0.56 (0.23–1.38)	0.2089	0.60 (0.23–1.53)	0.2802
	5 - Very high (≥90th pctl)	4.82 (3.05–7.60)	<0.0001	5.51 (3.46–8.79)	<0.0001	2.40 (1.33–4.33)	0.0037	2.35 (1.21–4.56)	0.0117
Cervix	1 - Very low (≤10th pctl)	0.77 (0.51–1.16)	0.2134	0.70 (0.46–1.06)	0.0926	0.69 (0.45–1.07)	0.0939	0.65 (0.40–1.07)	0.092
	2 - Low (>10 to ≤25th pctl)	0.62 (0.39–0.99)	0.0453	0.58 (0.36–0.92)	0.0203	0.65 (0.40–1.05)	0.078	0.65 (0.39–1.08)	0.0954
	3 - Medium (>25 to <75th pctl)	1.00 (Reference)		1.00 (Reference)		1.00 (Reference)		1.00 (Reference)	
	4 - High (≥75 to <90th pctl)	2.73 (1.94–3.85)	<0.0001	2.73 (1.94–3.86)	<0.0001	1.84 (1.27–2.67)	0.0014	1.80 (1.16–2.77)	0.0081
	5 - Very high (≥90th pctl)	4.73 (3.31–6.74)	<0.0001	4.79 (3.35–6.87)	<0.0001	2.66 (1.80–3.92)	<0.0001	2.64 (1.68–4.13)	<0.0001
Testis	1 - Very low (≤10th pctl)	1.59 (0.48–5.27)	0.4515	1.24 (0.35–4.41)	0.7409	0.41 (0.09–1.79)	0.2362	*NA*	

	2 - Low (>10 to ≤25th pctl)	0.68 (0.14–3.20)	0.6244	0.55 (0.12–2.65)	0.4593	0.34 (0.04–3.15)	0.3398	*NA*	
	3 - Medium (>25 to <75th pctl)	1.00 (Reference)		1.00 (Reference)		1.00 (Reference)		*NA*	
	4 - High (≥75 to <90th pctl)	0.48 (0.06–3.84)	0.4889	0.37 (0.05–3.10)	0.3623	-		*NA*	
	5 - Very high (≥90th pctl)	6.61 (2.47–17.71)	0.0002	7.51 (2.74–20.59)	<0.0001	0.95 (0.25–3.66)	0.9399	*NA*	
Endometrium	1 - Very low (≤10th pctl)	1.82 (1.41–2.35)	<0.0001	1.49 (1.15–1.92)	0.0026	1.29 (0.95–1.76)	0.0985	1.30 (0.95–1.77)	0.1027
	2 - Low (>10 to ≤25th pctl)	1.25 (0.95–1.65)	0.1067	1.14 (0.86–1.50)	0.3552	1.17 (0.85–1.61)	0.3274	1.17 (0.83–1.65)	0.3724
	3 - Medium (>25 to <75th pctl)	1.00 (Reference)		1.00 (Reference)		1.00 (Reference)		1.00 (Reference)	
	4 - High (≥75 to <90th pctl)	2.90 (2.30–3.66)	<0.0001	3.05 (2.41–3.85)	<0.0001	2.44 (1.83–3.23)	<0.0001	2.45 (1.83–3.28)	<0.0001
	5 - Very high (≥90th pctl)	7.80 (6.30–9.66)	<0.0001	8.73 (7.04–10.82)	<0.0001	5.21 (3.97–6.83)	<0.0001	5.20 (3.88–6.97)	<0.0001
Brain	1 - Very low (≤10th pctl)	1.69 (1.50–1.91)	<0.0001	1.43 (1.27–1.62)	<0.0001	*NA*		*NA*	
	2 - Low (>10 to ≤25th pctl)	1.20 (1.05–1.37)	0.0081	1.13 (0.99–1.30)	0.0635	*NA*		*NA*	
	3 - Medium (>25 to <75th pctl)	1.00 (Reference)		1.00 (Reference)		*NA*		*NA*	
	4 - High (≥75 to <90th pctl)	1.10 (0.94–1.29)	0.2457	1.15 (0.97–1.35)	0.0984	*NA*		*NA*	
	5 - Very high (≥90th pctl)	1.24 (1.05–1.48)	0.0135	1.26 (1.06–1.49)	0.0095	*NA*		*NA*	
Melanoma	1 - Very low (≤10th pctl)	1.32 (0.93–1.87)	0.1221	1.14 (0.80–1.62)	0.4773	0.99 (0.63–1.57)	0.9707	0.92 (0.53–1.58)	0.7537
	2 - Low (>10 to ≤25th pctl)	0.91 (0.64–1.28)	0.5878	0.86 (0.61–1.22)	0.404	0.70 (0.43–1.14)	0.1517	0.69 (0.42–1.14)	0.1515
	3 - Medium (>25 to <75th pctl)	1.00 (Reference)		1.00 (Reference)		1.00 (Reference)		1.00 (Reference)	
	4 - High (≥75 to <90th pctl)	1.84 (1.39–2.43)	<0.0001	1.95 (1.47–2.58)	<0.0001	1.52 (1.06–2.18)	0.0221	1.53 (1.03–2.26)	0.0334
	5 - Very high (≥90th pctl)	5.36 (4.21–6.82)	<0.0001	5.38 (4.22–6.85)	<0.0001	3.35 (2.47–4.54)	<0.0001	3.19 (2.23–4.56)	<0.0001
Head and neck	1 - Very low (≤10th pctl)	1.08 (0.83–1.41)	0.5494	0.95 (0.73–1.24)	0.7299	0.93 (0.68–1.26)	0.6262	0.97 (0.69–1.37)	0.8701
	2 - Low (>10 to ≤25th pctl)	0.66 (0.50–0.87)	0.0037	0.61 (0.46–0.81)	0.0006	0.61 (0.44–0.83)	0.0021	0.57 (0.41–0.80)	0.001
	3 - Medium (>25 to <75th pctl)	1.00 (Reference)		1.00 (Reference)		1.00 (Reference)		1.00 (Reference)	
	4 - High (≥75 to <90th pctl)	1.61 (1.28–2.02)	<0.0001	1.64 (1.31–2.06)	<0.0001	1.58 (1.22–2.06)	0.0005	1.54 (1.17–2.02)	0.0018
	5 - Very high (≥90th pctl)	3.34 (2.73–4.08)	<0.0001	3.42 (2.79–4.19)	<0.0001	2.97 (2.35–3.75)	<0.0001	2.76 (2.14–3.56)	<0.0001
Other solid tumour	1 - Very low (≤10th pctl)	1.31 (1.18–1.45)	<0.0001	1.21 (1.09–1.34)	0.0002	1.13 (0.91–1.39)	0.2687	1.13 (0.90–1.43)	0.2911
	2 - Low (>10 to ≤25th pctl)	0.86 (0.78–0.95)	0.0044	0.80 (0.72–0.88)	<0.0001	0.83 (0.67–1.03)	0.0936	0.83 (0.66–1.05)	0.1201
	3 - Medium (>25 to <75th pctl)	1.00 (Reference)		1.00 (Reference)		1.00 (Reference)		1.00 (Reference)	
	4 - High (≥75 to <90th pctl)	1.80 (1.65–1.97)	<0.0001	1.78 (1.63–1.94)	<0.0001	1.59 (1.30–1.94)	<0.0001	1.58 (1.28–1.96)	<0.0001
	5 - Very high (≥90th pctl)	2.54 (2.32–2.79)	<0.0001	2.62 (2.39–2.88)	<0.0001	2.34 (1.92–2.85)	<0.0001	2.34 (1.86–2.94)	<0.0001

HR = Hazard ratio; pctl = percentile; NA = Not applicable; *SS* = data suppressed due to small number of observations/events. * Adjusted for age at diagnosis (cat.), year of diagnosis (cont.), and common chronic conditions (asthma, congestive heart failure, chronic obstructive pulmonary disease, hypertension, and diabetes). ** Adjusted for age at diagnosis (cat.), year of diagnosis (cont.), common chronic conditions (asthma, congestive heart failure, chronic obstructive pulmonary disease, hypertension, and diabetes), and cancer stage. Only subjects with known cancer stage were included in the analysis. *** Adjusted for age at diagnosis (cat.), year of diagnosis (cont.), common chronic conditions (asthma, congestive heart failure, chronic obstructive pulmonary disease, hypertension, and diabetes), cancer stage, chemotherapy, and radiotherapy. Only subjects with known cancer stage were included in the analysis. Time-dependent confounding adjustment using marginal structural models.

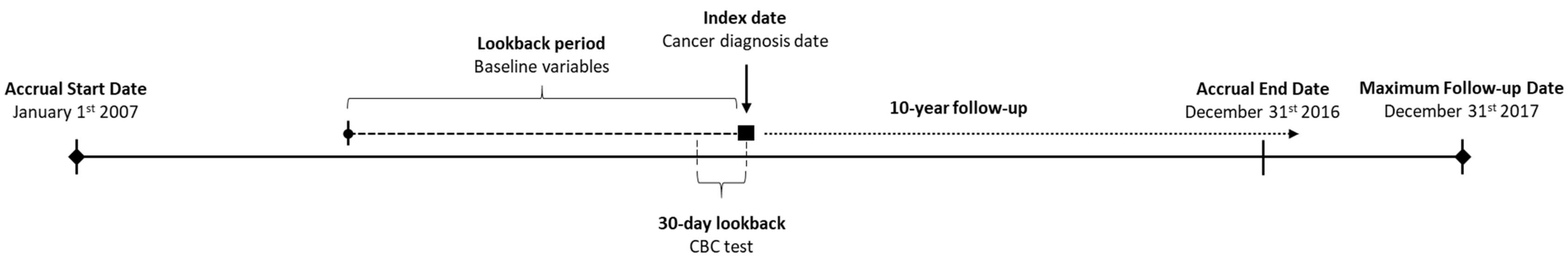

Figure S1. Study design and inclusion criteria among cancer patients.

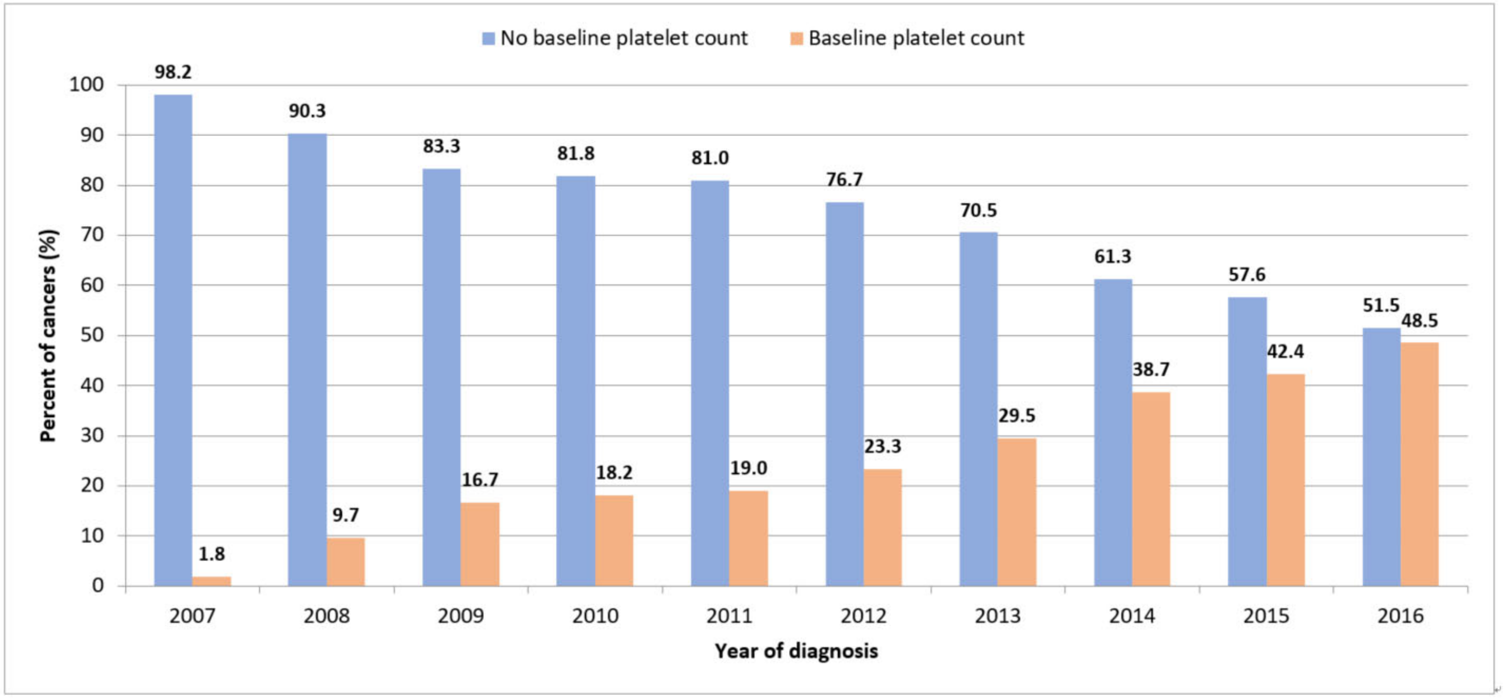

Figure S2. Proportion of cancer patients with a baseline platelet count (within 30 days of cancer diagnosis), by calendar year.

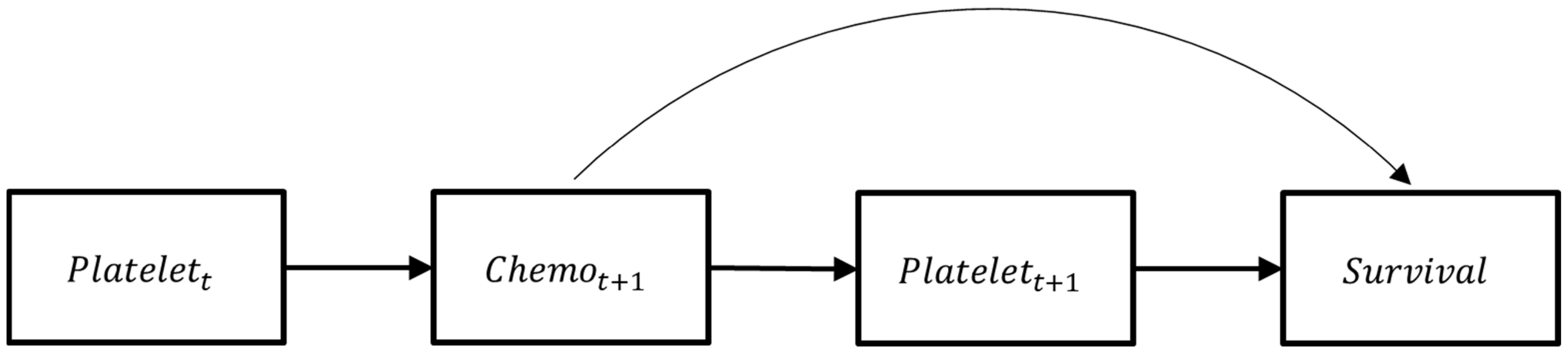

Figure S3. Schematic of time-dependent confounding that could occur between platelet count and cancer treatment.

(1) Platelet count at time *t* may influence treatment decision for chemotherapy at time *t+1.*

(2) Chemotherapy at *t+1* is causally associated with a higher cancer-specific survival.

(3) Chemotherapy at time *t+1* influences platelet count at time *t+1.*

(4) Platelet count at time *t+1* may be associated with survival.

Exposure weight models:

Numerator model

$$logit\left(P\left(Y_i = y \middle| \beta_0 + \sum \beta_j x_j\right)\right) = \beta_0 + \sum \beta_j x_j$$

Outcome: platelet count category (at time t)

Predictors: prior platelet count category (t-1), age at diagnosis, year of diagnosis, asthma, CHF, COPD, hypertension, diabetes, and cancer stage.

Denominator model

$$logit\left(P\left(Y_i = y \middle| \beta_0 + \sum \beta_j x_j\right)\right) = \beta_0 + \sum \beta_j x_j$$

Outcome: platelet count category (at time t)

Predictors: prior platelet count category (t-1), chemotherapy use (at time t), radiotherapy use (at time t), age at diagnosis, year of diagnosis, asthma, CHF, COPD, hypertension, diabetes, and cancer stage.

Censor weight models:

Numerator model

$$logit\left(P\left(Y = 1 \middle| \beta_0 + \sum \beta_j x_j\right)\right) = \beta_0 + \sum \beta_j x_j$$

Outcome: Censoring event

Predictors: prior platelet count category (t-1), age at diagnosis, year of diagnosis, asthma, CHF, COPD, hypertension, diabetes, and cancer stage.

Denominator model

$$logit\left(P\left(Y = 1 \middle| \beta_0 + \sum \beta_j x_j\right)\right) = \beta_0 + \sum \beta_j x_j$$

Outcome: Censoring event

Predictors: prior platelet count category (t-1), chemotherapy use (at time t), radiotherapy use (at time t), age at diagnosis, year of diagnosis, asthma, CHF, COPD, hypertension, diabetes, and cancer stage.

Standardized weights calculations:

$$SW_t = \frac{\prod_{i=1}^{t} P_{treat}(Y_i = y)_{num} / P_{treat}(Y_i = y)_{denom}}{\prod_{i=1}^{t} [((Y_{cens} = 1) \times P_{cens}(Y = 1)_{num} + (Y_{cens} = 0) \times (1 - P_{cens}(Y = 1)_{num}) / ((Y_{cens} = 1) \times P_{cens}(Y = 1)_{denom} + (Y_{cens} = 0) \times (1 - P_{cens}(Y = 1)_{denom})]}$$

$P_{treat}(Y_i = y)_{num}$ Probability of being in treatment class y at time t in the numerator model

$P_{treat}(Y_i = y)_{denom}$ Probability of being in the treatment class y at time t in the denominator model

$P_{cens}(Y = 1)_{num}$ Probability of being censored at time t in the numerator model

$P_{cens}(Y = 1)_{denom}$ Probability of being censored at time t in the denominator model

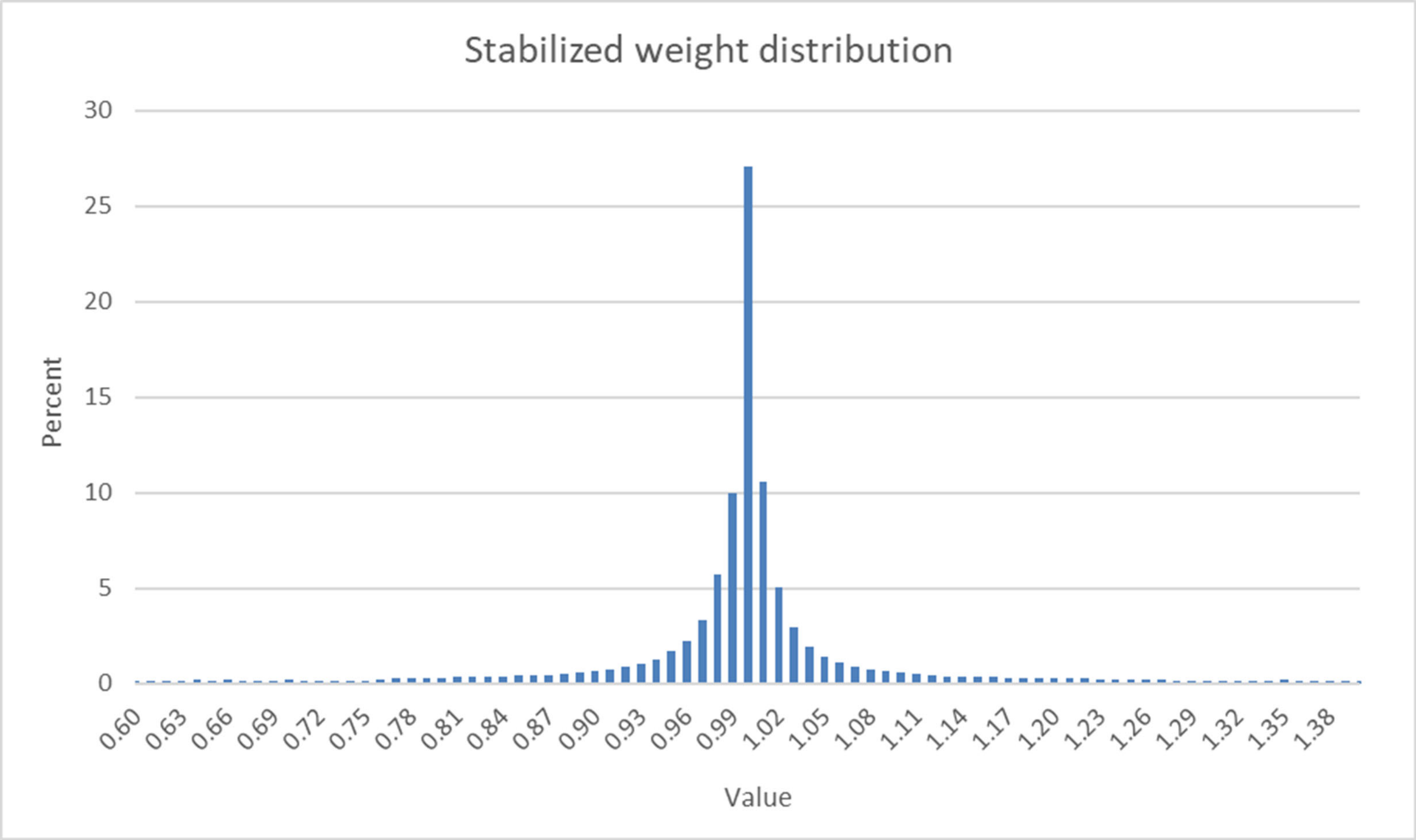

Figure S4. Distribution of stabilized weights among observations included in the marginal structural models (exposure definition 1).

N	10,881,327
Mean	1.0099
Standard Deviation	0.3291
Minimum	0.0614
Maximum	14.1718

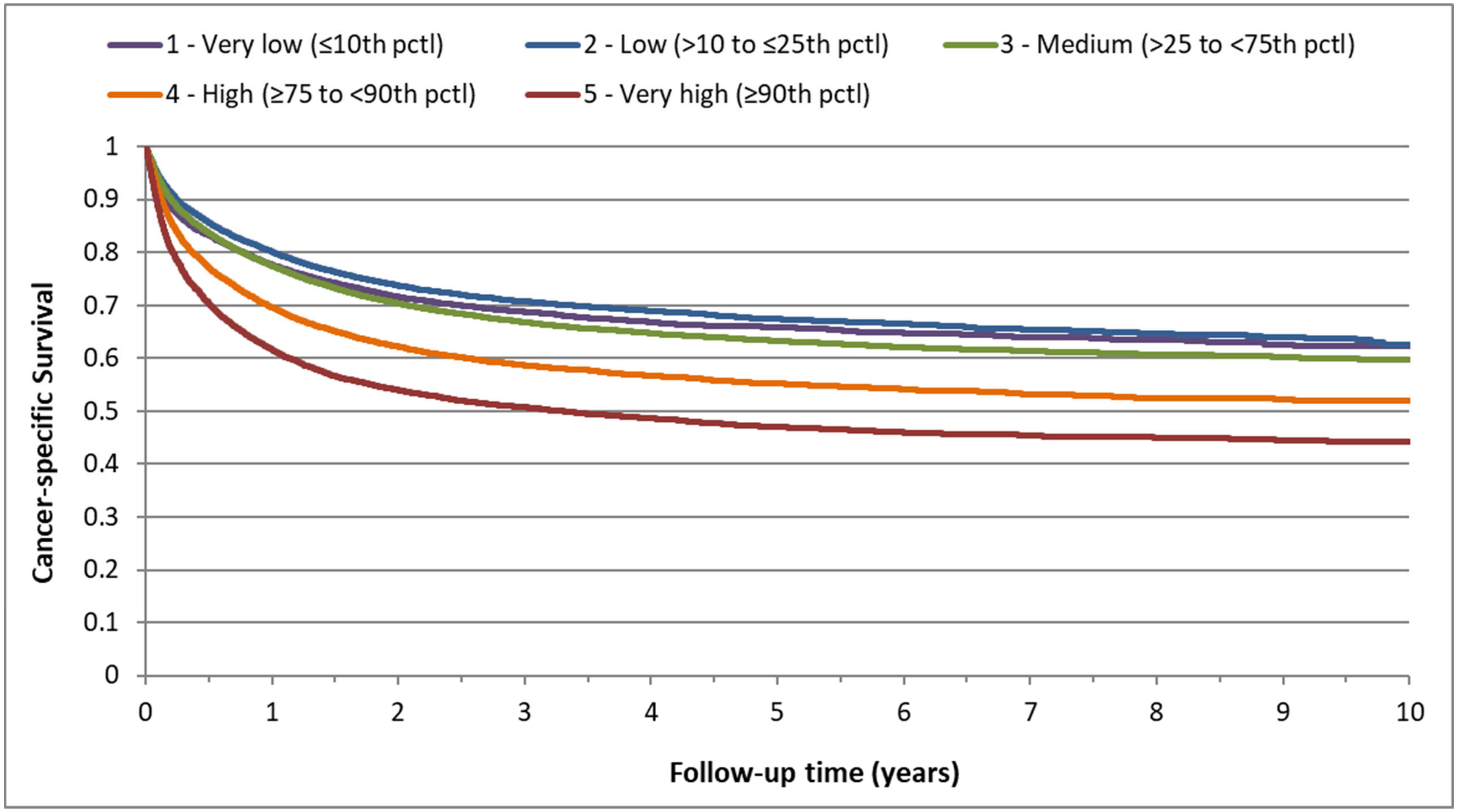

(A) All cancers (n = 112,231)

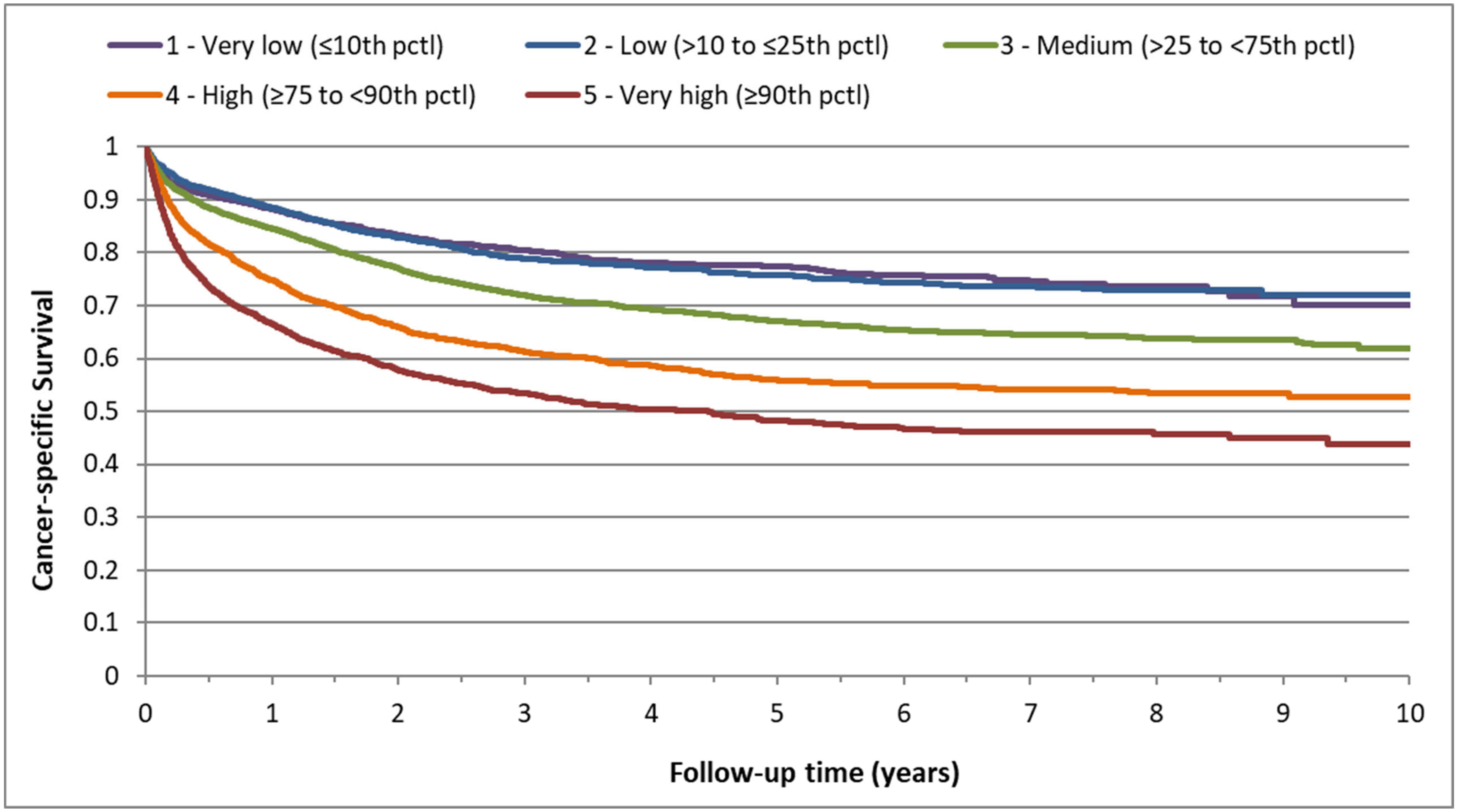

(B) Colon (n = 17,259)

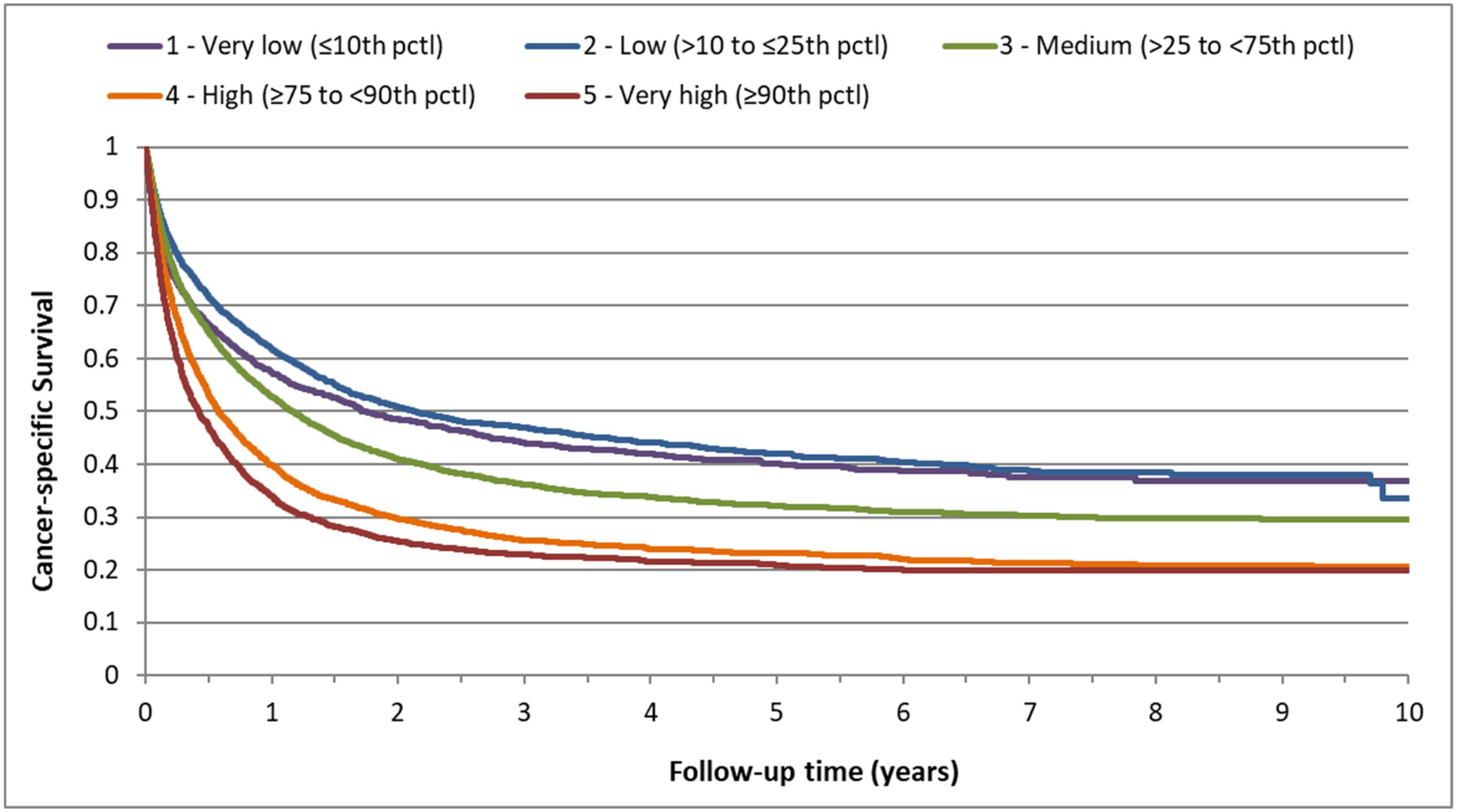

(C) Lung (n = 20,583)

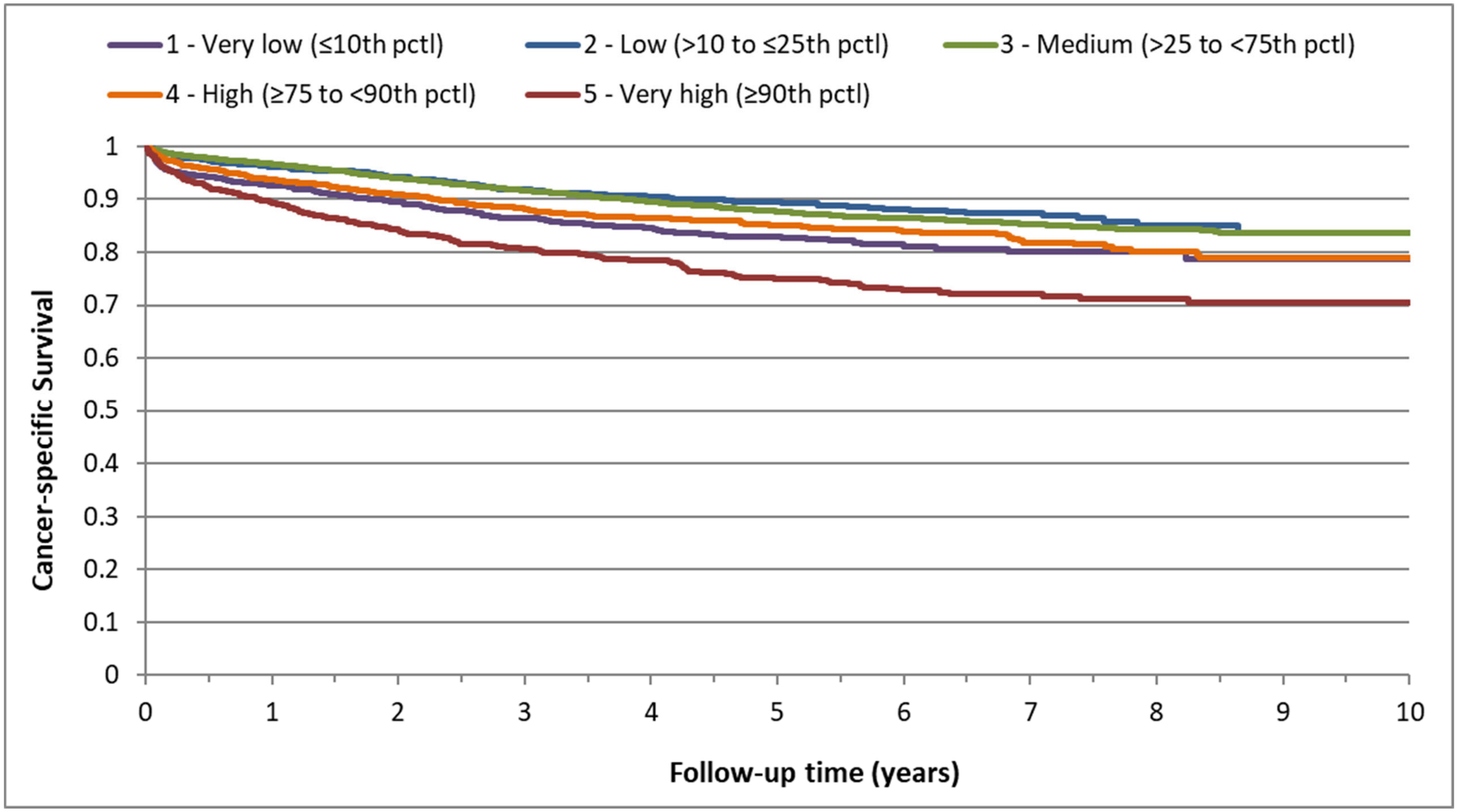

(D) Breast (n = 9,857)

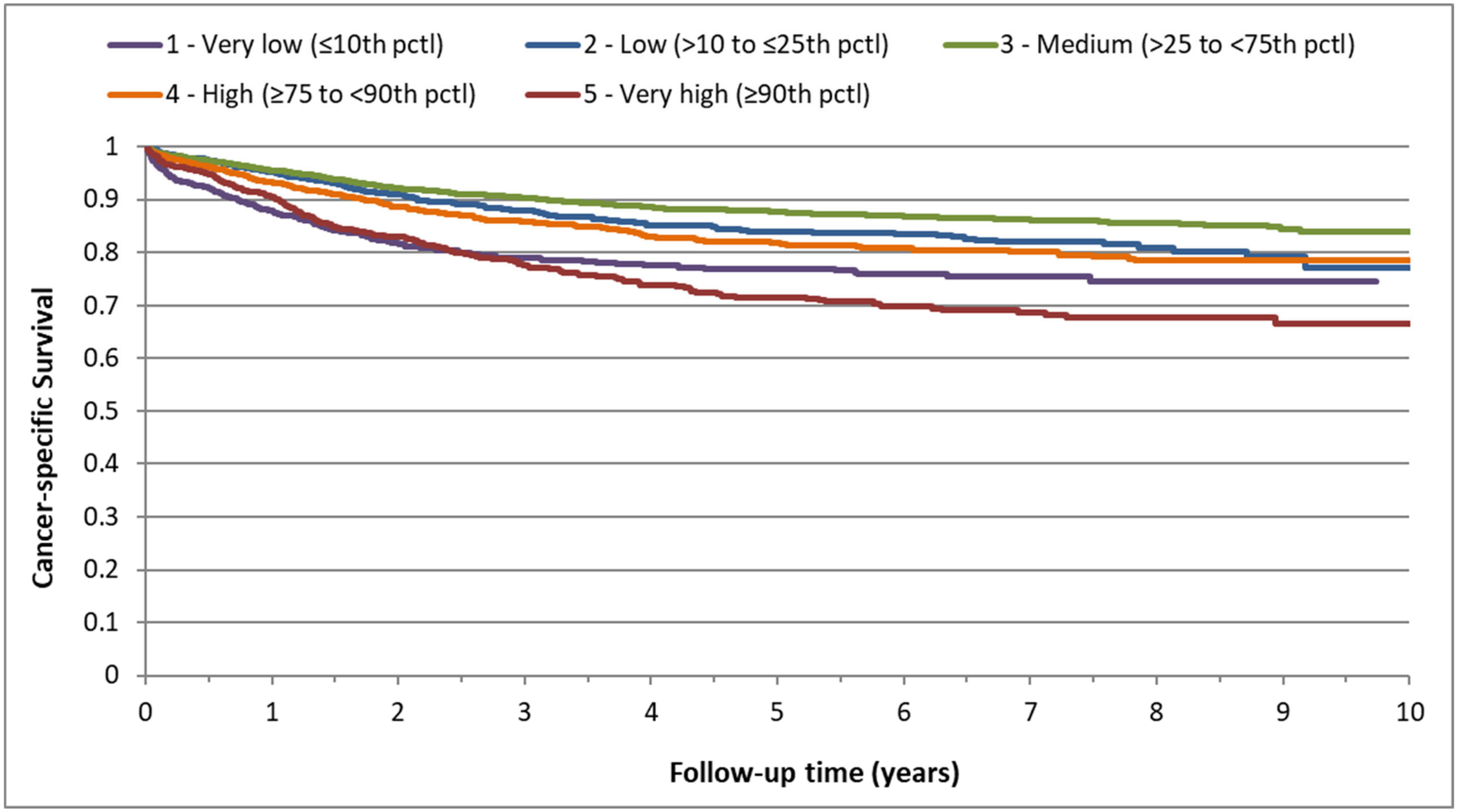

(E) Prostate (n = 8,587)

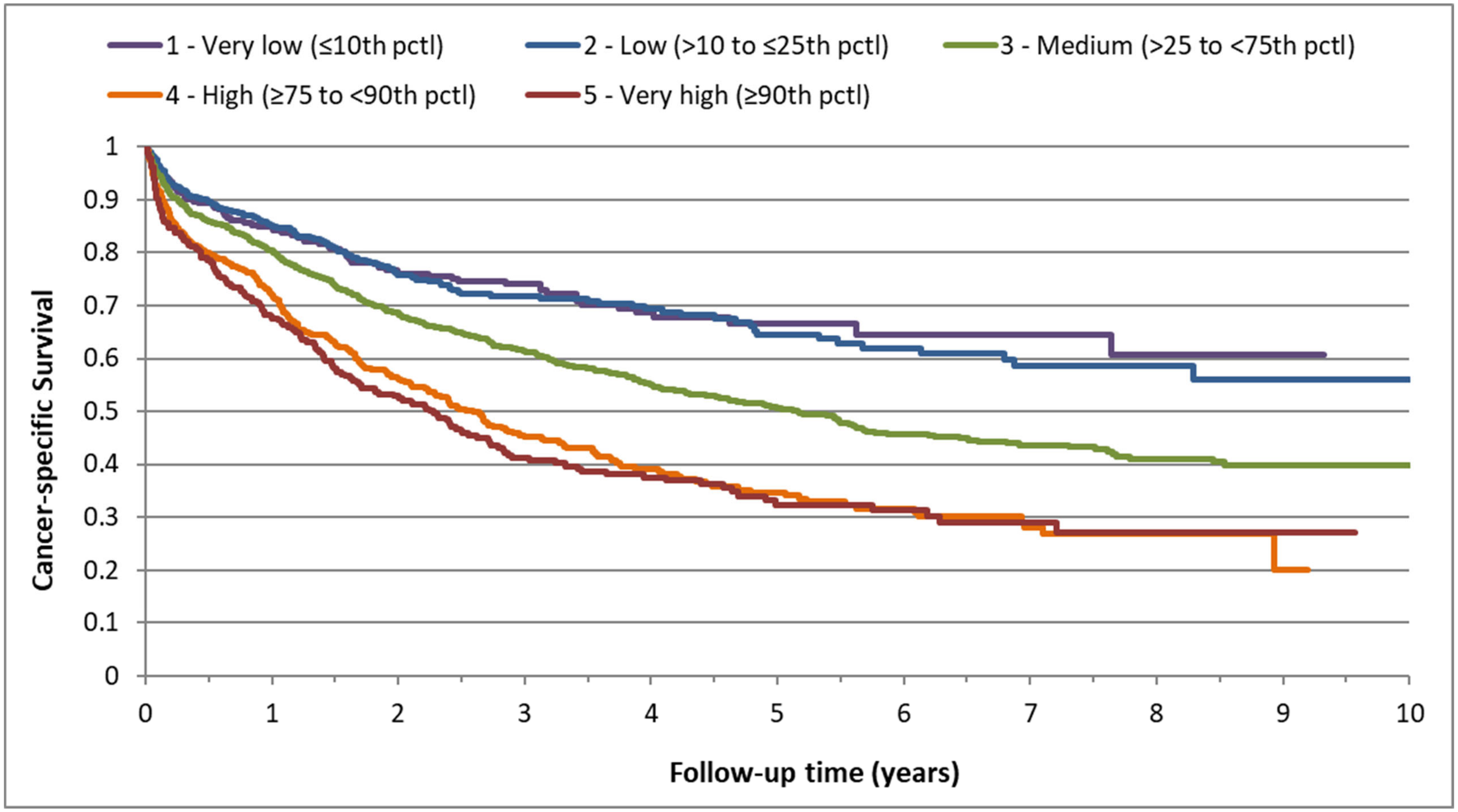

(F) Ovary (n = 3,085)

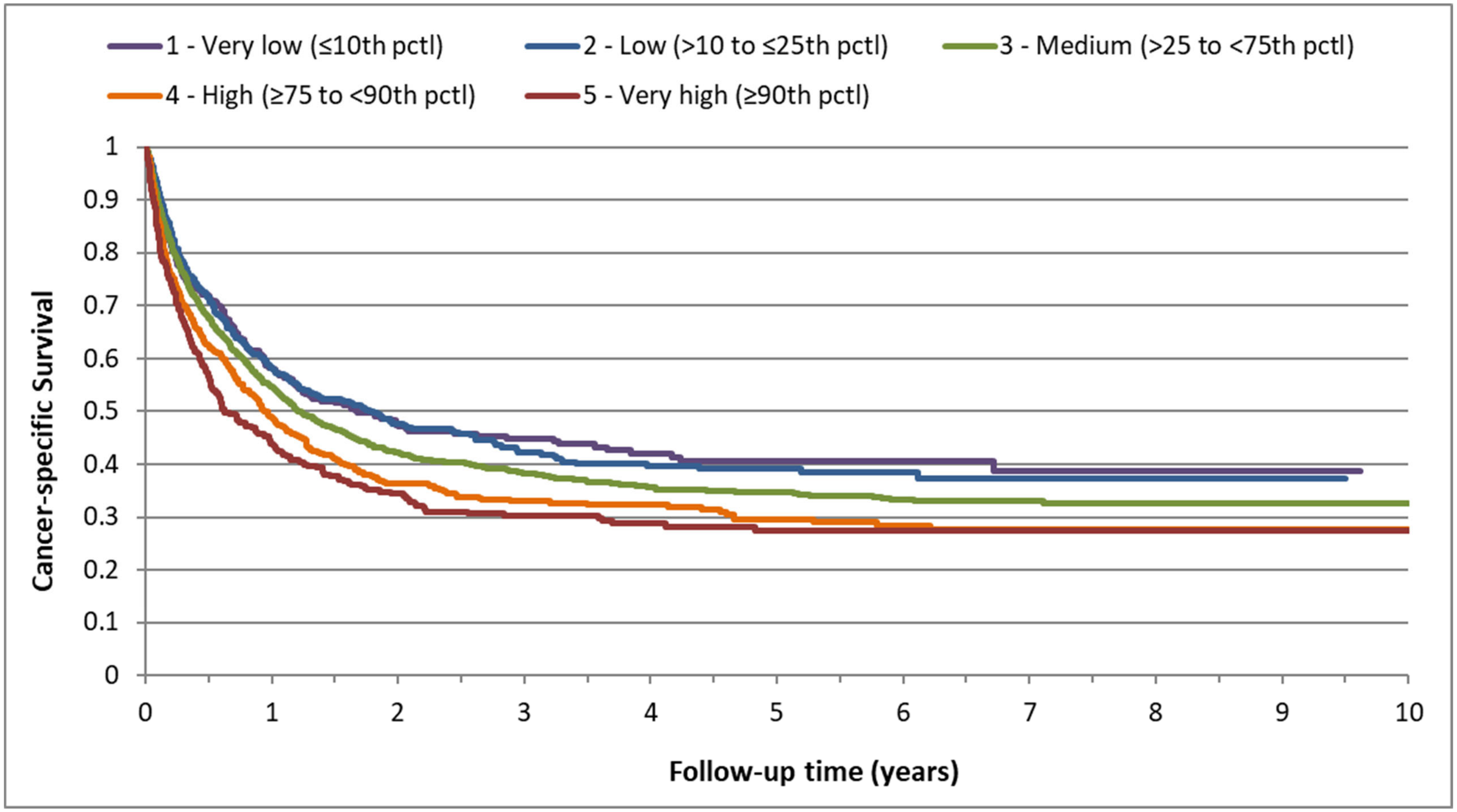

(G) Stomach (n = 3,365)

Figure S5. Cancer-specific survival by baseline platelet count category (exposure definition 2), for select cancers.

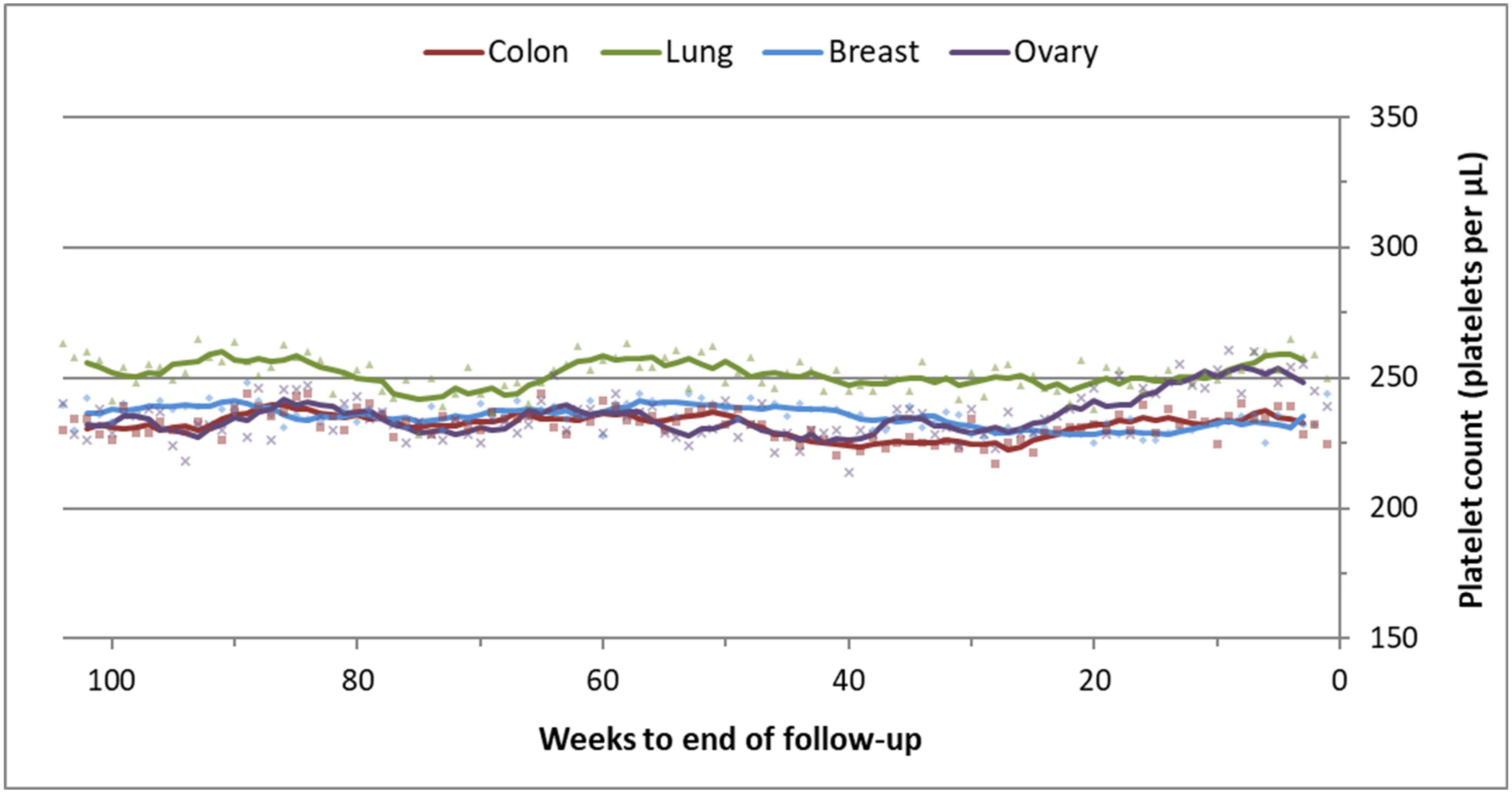

(A) Female patients

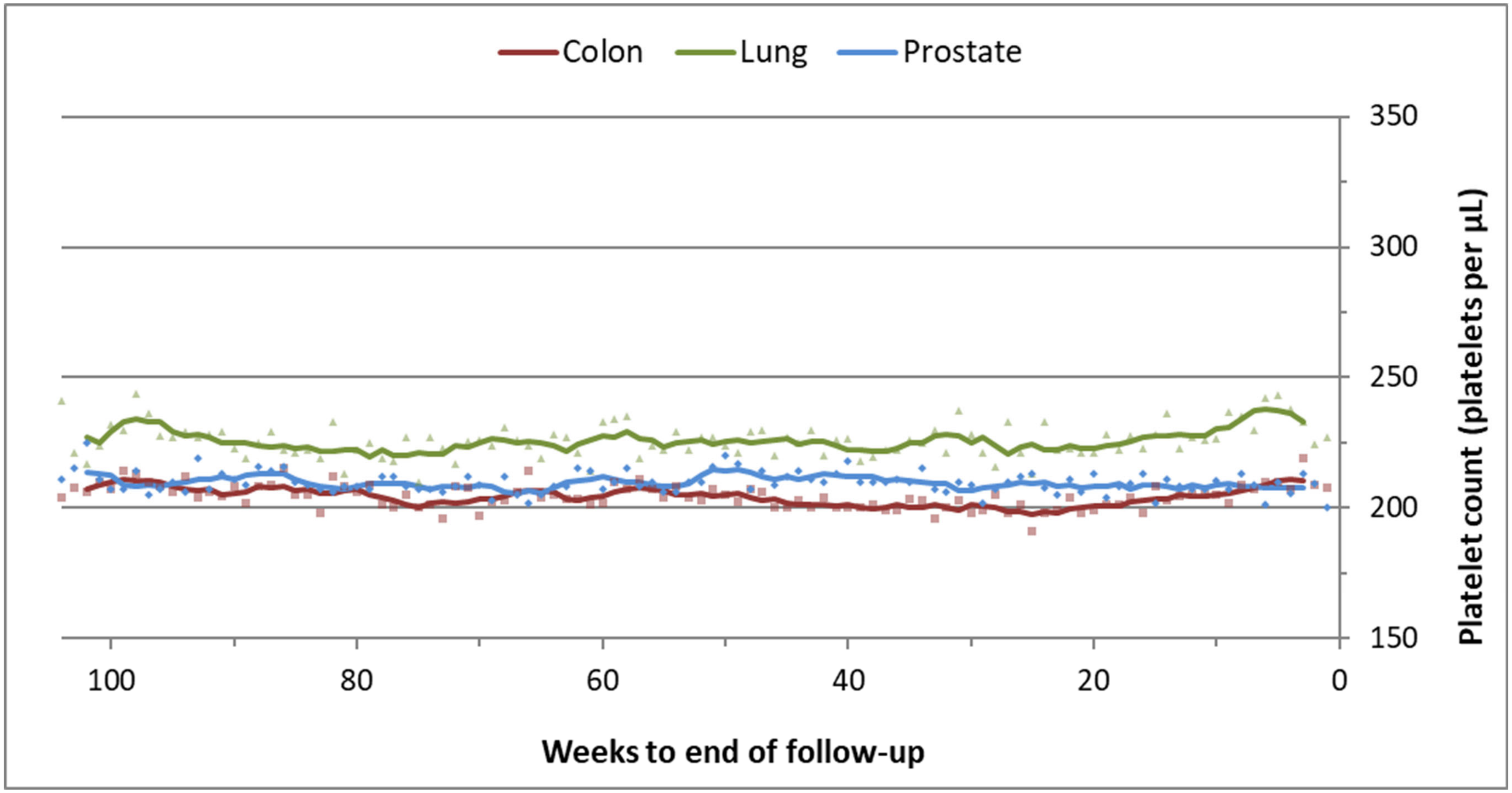

(B) Male patients

Figure S6. Median platelet count moving average (period of 5) in the 104 weeks (2 years) preceding end of follow-up, among patients that did not die from their index cancer.

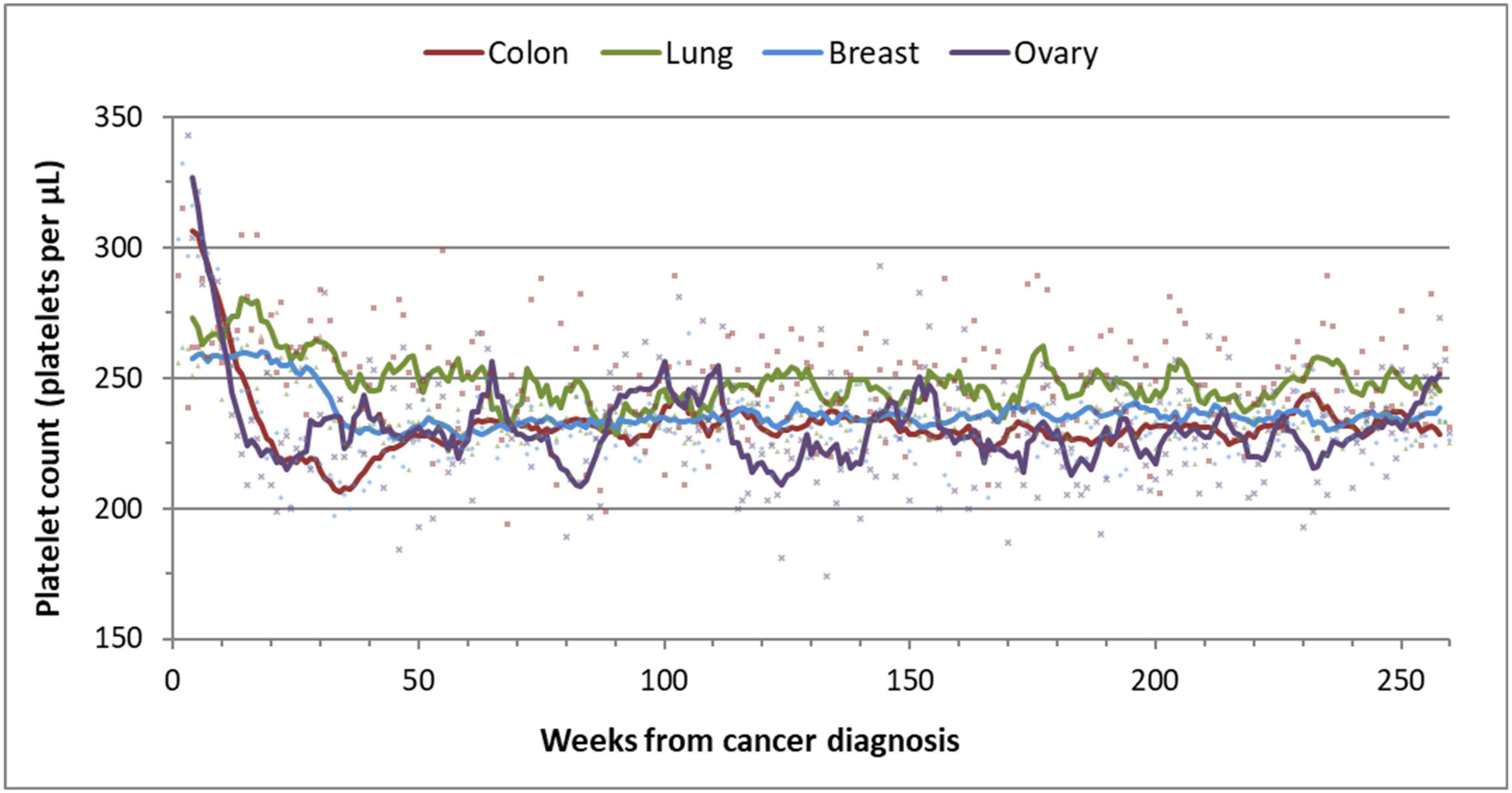

(A) Female patients

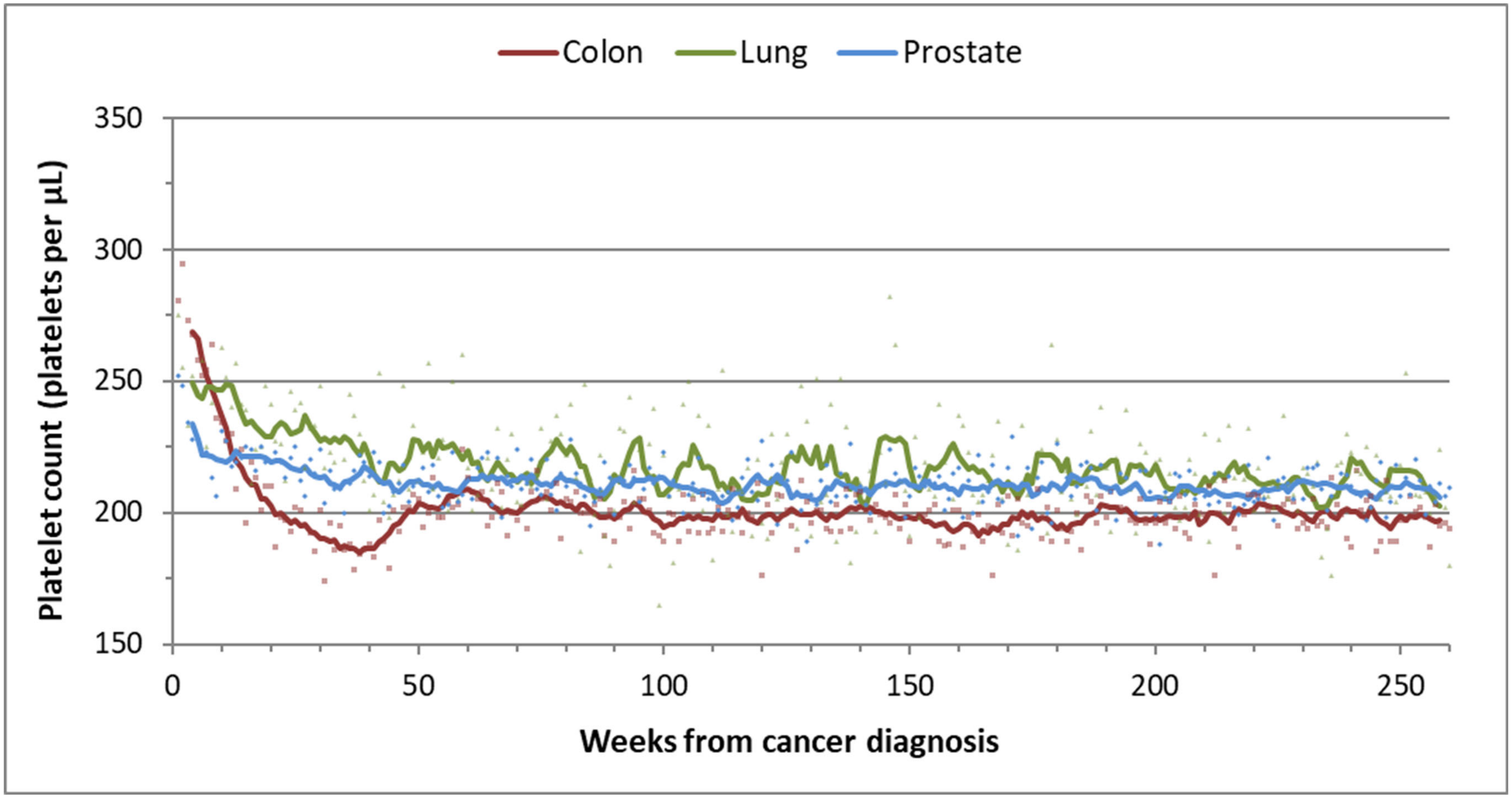

(B) Male patients

Figure S7. Median platelet count moving average (period of 7) in the 260 weeks (5 years) following a cancer diagnosis, among patients with at least 5 years of follow-up and did not die from their index cancer.

References

1. Shah, B.R.; Chiu, M.; Amin, S.; Ramani, M.; Sadry, S.; Tu, J.V. Surname lists to identify South Asian and Chinese ethnicity from secondary data in Ontario, Canada: A validation study. *BMC Med. Res. Methodol*. **2010**, *10*, 42.
2. Robles, S.C.; Marrett, L.D.; Clarke, E.A.; Risch, H.A. An application of capture-recapture methods to the estimation of completeness of cancer registration. *J. Clin Epidemiol*. **1988**, *41*, 495–501.
3. Gershon, A.S.; Guan, J.; Wang, C.; To, T. Trends in asthma prevalence and incidence in Ontario, Canada, 1996–2005: A population study. *Am. J. Epidemiol* **2010**, *172*, 728–36.
4. Schultz, S.E.; Rothwell, D.M.; Chen, Z.; Tu, K. Identifying cases of congestive heart failure from administrative data: A validation study using primary care patient records. *Chronic Dis Inj Can.* **2013**, *33*, 160–6.
5. Gershon, A.S.; Wang, C.; Guan, J.; Vasilevska-Ristovska, J.; Cicutto, L.; To, T. Identifying individuals with physcian diagnosed COPD in health administrative databases. *COPD* **2009**, *6*, 388–94.
6. Jaakkimainen, R.L.; Bronskill, S.E.; Tierney, M.C.; et, al. Identification of Physician-Diagnosed Alzheimer's Disease and Related Dementias in Population-Based Administrative Data: A Validation Study Using Family Physicians' Electronic Medical Records. *J. Alzheimers Dis* **2016**, *54*, 337–49.
7. Antoniou, T.; Zagorski, B.; Loutfy, M.R.; Strike, C.; Glazier, R.H. Validation of case-finding algorithms derived from administrative data for identifying adults living with human immunodeficiency virus infection. *PLoS ONE* **2011**, *6*, e21748.
8. Tu, K.; Campbell, N.R.; Chen, Z.L.; Cauch-Dudek, K.J.; McAlister, F.A. Accuracy of administrative databases in identifying patients with hypertension. *Open Med.* **2007**, *1*, e18–26.
9. Benchimol, E.I.; Guttmann, A.; Mack, D.R.; et al. Validation of international algorithms to identify adults with inflammatory bowel disease in health administrative data from Ontario, Canada. *J. Clin Epidemiol* **2014**, *67*, 887–96.
10. Lipscombe, L.L.; Hwee, J.; Webster, L.; Shah, B.R.; Booth, G.L.; Tu, K. Identifying diabetes cases from administrative data: A population-based validation study. *BMC Health Serv* Res **2018**, *18*, 316.
11. Widdifield, J.; Bombardier, C.; Bernatsky, S.; et al. An administrative data validation study of the accuracy of algorithms for identifying rheumatoid arthritis: The influence of the reference standard on algorithm performance. *BMC Musculoskelet Disord* **2014**, *15*, 216.
12. Mondor, L.; Maxwell, C.J.; Hogan, D.B.; et al. Multimorbidity and healthcare utilization among home care clients with dementia in Ontario, Canada: A retrospective analysis of a population-based cohort. *PLoS Med.* **2017**, *14*, e1002249.
